# A Systematic Review on Comparative Analysis, Toxicology, and Pharmacology of Medicinal Plants Against *Haemonchus contortus*

**DOI:** 10.3389/fphar.2021.644027

**Published:** 2021-05-10

**Authors:** Rehman Ali, Muhammad Rooman, Sakina Mussarat, Sadia Norin, Shandana Ali, Muhammad Adnan, Shahid Niaz Khan

**Affiliations:** ^1^ Department of Zoology, Faculty of Biological Sciences, Kohat University of Science and Technology, Kohat, Pakistan; ^2^ Department of Zoology, Hazara University Mansehra, Kohat, Pakistan; ^3^ Department of Botanical and Environmental Sciences, Faculty of Biological Sciences, Kohat University of Science and Technology, Kohat, Pakistan

**Keywords:** medicinal plants, pharmacology, nematicidal activity, *Haemonchus contortus*, toxicology, ethnoveterinary, antiparasitic, anthelmintic

## Abstract

**Background:** *Haemonchus contortus* is an important pathogenic nematode parasite and major economic constraint of small ruminants in tropics and subtropics regions. This review is an attempt to systematically address the; (a) efficacy of different plants against *H. contortus* by *in vitro* and *in vivo* proof; (b) toxicology, mechanism of action, and active phyto-compounds involve in anti-haemonchiasis activity; (c) and comparative analysis of plant species evaluated both *in vitro* and *in vivo*.

**Methods:** Online databases (Google Scholar, PubMed, Scopus, and ScienceDirect) were searched and published research articles (1980–2020) were gathered and reviewed.

**Results:** A total of 187 plant species were reported belonging to 59 families and 145 genera with Asteraceae and Fabaceae being frequently used. Out of the total plant species, 171 species were found to be evaluated *in vitro* and only 40 species *in vivo*. Twenty-four species were commonly evaluated for *in vitro* and *in vivo* anti-haemonchiasis activity. Among the reported assays, egg hatching test (EHT) and fecal egg count reduction (FECR) were the most widely used assays *in vitro* and *in vivo*, respectively. Moreover, sheep were the frequently used experimental model *in vivo*. After comparative analysis, *Lachesiodendron viridiflorum*, *Corymbia citriodora*, *Calotropis procera*, and *Artemisia herba-alba* were found highly effective both *in vitro* and *in vivo*. *L. viridiflorum* inhibited enzymatic activities and metabolic processes of the parasite and was found to be safe without toxic effects. *C. citriodora* was moderately toxic *in vivo*, however, the plant extract produced promising nematicidal effects by causing muscular disorganization and changes in the mitochondrial profile. Additionally, *C. procera* and *A. herba* *-alba* despite of their high anti-haemonchiasis activity were found to be highly toxic at the tested concentrations. *C. procera* caused perforation and tegumental disorganization along with adult worm paralysis. Nineteen compounds were reported, among which anethole and carvone completely inhibited egg hatching *in vitro* and significantly reduced fecal egg count, decreased male length, and reproductive capacity of female *in vivo*.

**Conclusion:** This review summarized different medicinal plants owing to nematicidal activities against *H. contortus* eggs, larvae, and adult worms. Plants like *L. viridiflorum, C. citriodora, C. procera,* and *A. herba-alba*, while compounds anethole and carvone having promising nematicidal activities and could be an alternative source for developing novel drugs after further investigation.

## Introduction

*Haemonchus contortus* is the causative agent of haemonchiasis usually known as “twisted barber” pole worm or stomach worm (Saminathan et al., 2015), and a common blood feeder of small ruminants. The parasite is present throughout the tropical and subtropical regions of the world where it is a major constraint for profitable production of sheep and goats (Zenebe et al., 2017). Haemonchiasis is characterized by severe anemia, leading to a serious impairment of the animal, severe economic losses, and acute disease outbreaks with high death rate particularly in young animals (Selemon, 2018). Among the parasitic diseases, gastrointestinal nematode infections remain one of the main causes of impaired production in small ruminants (Hounzangbe-Adote et al., 2005). According to the pharmaceutical companies the annual cost of antiparasitic compounds is proposed to be tens of billions of dollars worldwide (Wolstenholme et al., 2004). However, the annual treatment cost of *H. contortus* has been estimated to be 26 million USD in Kenya, 46 million USD in South Africa, and 103 million USD in India (Peter and Chandrawathani, 2005).

To overcome the major economic losses in agriculture, it is essential to enhance the control of key parasitic diseases (Gilleard, 2006). For this purpose, various approaches are being in use to control parasitism, including biological control, pasture management, dietary management, vaccination, and the use of anthelmintic chemicals. Most widely used practice being followed nowadays is the use of anthelmintic chemicals. Unfortunately, regular and indiscriminate administration has posed a variety of problems including emergence of resistance in nematode parasites, e.g. multi resistant *H. contortus* has been reported. Furthermore, the commercially available anthelmintics drugs are somewhat costly and smallholder farmers are unable to expend meager income for purchasing of drugs to carry on regular treatment (Irum et al., 2015).

As a result, there is a dire need to develop alternative anthelmintic approaches from natural flora which can be less toxic, biodegradable, environmental friendly, and to cover the most challenging problem of parasite resistance issue (Carvalho et al., 2012). Worldwide, different plant species have been reported and evaluated for natural bio-products to control the parasitic infections and reduce the dependency on conventional chemotherapy. Testing for biological activity *in vitro* and *in vivo* has to be done after a purification process in order to exclude interference with accompanying compounds and reference standards for quality control of herbal medicines largely depend on isolated compounds with documented purity (Bucar et al., 2013). The compounds from plants have also been found to be synergistic enhancers in that though they may not possess any anthelmintic properties alone, but when used concurrently with standard drugs they enhance the activity of the drug. The synergistic effect of the association of an anthelmintic drug and plant extracts against resistant pathogens leads to new choices for the treatment of infectious diseases. Also synergy between bioactive plant product and antiparasitic will confront problems of toxicity and overdose since lesser concentrations of two agents in combination are required, due to these reasons, there is need for continuous exploration of multidrug resistance modulating principles from plants sources (Aiyegoro and Okoh, 2009). The herbal medicines however, suffer from lack of standardization parameters. The main limitation is the lack of standardization of raw materials, processing methods and the final products, dosage formulation, and the non-existence of criteria for quality control (Sachan et al., 2016).

Recently, the interest of researchers in exploring the antiparasitic properties of ethnoveterinary medicinal plants is increasing and this field of research is inundated with ethnopharmacological studies. This review is aimed to gather fragmented literature about the; (a) efficacy of different plants against *H. contortus* by *in vitro* and *in vivo* proof; (b) toxicology, mechanism of action, and active phyto-compounds involve in anti-haemonchiasis activity; (c) and comparative analysis of plant species evaluated both *in vitro* and *in vivo*. Moreover, the study also highlights existing knowledge gaps in the present research and provides future recommendations to fulfill those gaps.

## Methodology

The systematic review was conducted according to the Preferred Reporting Items for Systematic Reviews and Meta-Analyses (PRISMA) guidelines (Moher et al., 2009). No protocol was followed for conducting this systematic review. The PRISMA check list is provided in the supporting information section (Supplementary Table S1).

### Databases and Searching Criteria

To find the published literature, a systematic search was performed using different databases, including Google Scholar, PubMed, Scopus, and ScienceDirect. Research articles published in English language from 1980 to 2020 were gathered for this systematic review. Key words such as: anthelmintic activity, nematicidal activity of plants, medicinal plants used for *H. contortus*, *in vitro*/*in vivo* efficacy of plants against *H. contortus*, active compounds in plants, mechanism of plant extract inhibition and toxicity of plants. “Anthelmintic AND *Hemonchus contortus*”, “Natural nematicidal OR anti-haemonchiasis NOT synthetic”, “Natural *in vitro* OR *in vivo* anthelmintic”. Bibliographies of research articles were also searched and relevant references were extracted and downloaded. Moreover, to support the findings of the review further literature search was conducted and relevant articles were included.

### Inclusion/Exclusion Criteria

Research articles describing (a) *in vitro*/*in vivo* efficacy of medicinal plants against *H. contortus*, (b) containing full information regarding plant name, country name, extract, concentration, inhibition, time exposure, and assay type, (c) original research articles, (d) and published in English language were included in this systematic review. While articles with (a) epidemiological and molecular dataset of *H. contortus*, (b) antiparasitic activities other than *H. contortus*, (c) synthetic drugs/chemicals tested against *H. contortus*, and (d) language other than English were excluded.

### Data Extraction

Endnote (Thomson Reuters, San Francisco, CA, United States) was used to compile the articles. Researchers very carefully extracted all the data from the selected articles including author (s) name, country name, plant name, family name, plant part used, plants’ life form, extract used, concentration, time exposure, inhibition, and year of publication. Data were arranged into tables and figures. Chemical structures of the compounds were drawn using MarvinSketch (18.24.0) (https://chemaxon.com/products/marvinn) and Inkscape (0.92) (https://inkscape.org/) was used to further refine and improve the resolution of each chemical structure. PubChem (https://pubchem.ncbi.nlm.nih.gov) was also used to attain the IUPAC name (s) of pure compounds reported in this review.

### Taxonomic Clarification

Plant scientific names, synonyms, and families were searched and corrected using “the plant list” (http://www.theplantlist.org), “tropicos” (http://www.tropicos.org), “world flora online” (http://www.worldfloraonline.org), and “Medicinal Plant Name Services-KEW” (https://mpns.science.kew.org/mpns-portal).

### Quantitative Analysis

#### Jaccard Similarity Index (JI)

Jaccard similarity index was calculated to determine the similarity between the two sets of studies reported in this review. One set of study is the “*in vitro* pharmacological validation of medicinal plants” and the other one is the “*in vivo* pharmacological validation of medicinal plants”. JI was calculated by using the formula (Kayani et al., 2015):

JI=c multiply 100/a+b−c

Where “a” is the total number of plant species used *in vitro*, “b” is the total number of plant species used *in vivo* as anthelmintic against *H. contortus*, and “c” is the number of plant species common to both *in vitro* and *in vivo* studies.

## Results

**FIGURE 1:**
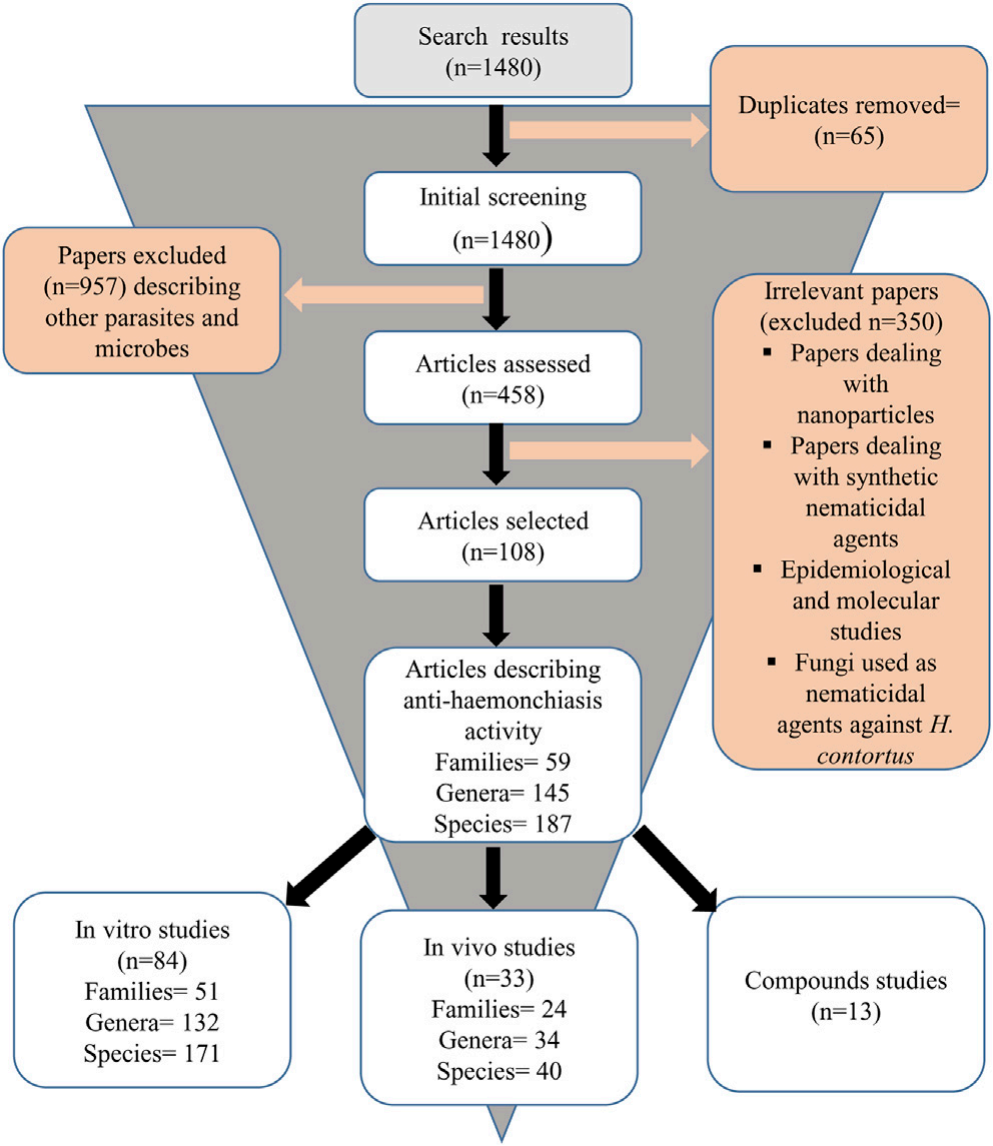
Flow chart of screening process of articles.

We identified a total of 1,480 published articles through literature search. After removing duplicates, and irrelevant articles, a total of 108 articles were selected for this review (Figure 1). Quality assessment of the selected articles was performed and summarized as author name, species/compound(s) stated in the article, plant source, species authentication, quality control as well as chemical analysis reported (Table 1).

**TABLE 1:** Quality assessment of the articles selected for this systematic review.

Study	Species/compound stated in the article	Plant source	Authenticated species	Quality control reported?	Chemical analysis reported?
Cala et al. (2012)	*Melia azedaracha* L	Not stated	−	Yes	Yes-liquid-liquid chromatography
	*Trichilia claussenii* C				
Irum et al. (2015)	*Artemisia vestita* Wall ex Besser	Collected from northern areas of Pakistan	+	No	No
	*Seriphidium maritimum* (L.) Poljakov (=*Artemisia maritima* L.)				
Jaheed et al. (2019)	*Balanites egyptiaca* (L.) Delile	Purchased from local market, upper Egypt	−	Yes	Yes-gas chromatography–mass spectrometry (GC–MS) analysis
Katiki et al. (2019)	Anethole and carvone	Appalachian farming systems research left	−	Yes	No
Lone et al. (2012)	*Euphorbia helioscopia* L	Kashmir valley, India	+	No	No
Lopes et al. (2018)	*Anacardium occidentale* L	NatVita in eusébio, ceará	−	Yes	Yes-liquid Chromatography-Mass spectroscopy (LC-MS) analysis
Maphosa et al. (2010)	*Elephantorrhiza elephantina* (Burch.) Skeels	Ntselamanzi, nkonkobe muncipality, eastern cape province, South Africa	+	Yes	No
	*Aloe ferox* Mill.				
	*Leonotis leonurus* (L.) R. Br				
Marie-Magdeleine et al. (2014)	*Musa x paradisiaca* L	Guadeloupe French west indies	−	No	No
Minho et al. (2008)	*Acacia decurrens* (J.C.Wendl.) Willd. (=*Acacia molissima* Willd.)	Not stated	−	Yes	No
Monteiro et al. (2011)	*Jatropha curcas* L	Obtained from embrapa eastern amazon, don eliseu	+	Yes	No
Njoku and Asuzu (1998)	*Ocimum gratissimum* L	Collected from nsukka in enugu state Nigeria	+	Yes	No
Nsereko et al. (2019)	*Senna occidentalis* (L.) Link. (=*Cassia occidentalis* L.)	Uganda	+	Yes	No
	*Euphorbia hirta* L				
Oliveira et al. (2009)	*Cocos nucifera* L	Agroindustria tropical located in fortaleza, ceara state university	-	Yes	No
Ademola and Eloff (2011)	*Senna alata* (L.) Roxb. (=*Cassia alata*)	Collected from zaria, Nigeria	+	No	No
Martínez-Ortiz-de-Montellano et al. (2019)	*Lysiloma latisiliquum* (L.) Benth.	Harvested from tropical forest in merida, yucatan, Mexico	−	Yes	No
	*Onobrychis viciifolia* Scop				
Pessoa et al. (2002)	*Ocimum gratissimum* L	Fortaleza, ceará, northeast of Brazil	+	Yes	Yes-method not stated
Iqbal et al. (2006b)	*Nicotiana tabacum* L	Purchased from local market in faisalabad, Pakistan	+	Yes	No
Sirama et al. (2015)	*Gymnanthemum amygdalinum* (Delile) Sch.Bip. (=*Vernonia amygdalina* Del.)	Not stated	−	Yes	No
Squires et al. (2010)	*Artemisia annua* L	Appalachian farming systems research left	−	No	No
	*Artemisia absinthium* L				
Maphosa and Masika (2012)	*Elephantorrhiza elephantina* (Burch.) Skeels	Matatiele district, eastern cape province, South Africa	−	Yes	No
Maciel et al. (2006)	*Azadirachta indica* A. Juss. (=*Melia azedarach* L.)	Not stated	−	Yes	No
Macedo et al. (2010)	*Eucalyptus staigeriana* F. Muell. Ex F.M. Bailey	Dierberguer óleos essenciais ltda (barra bonita, são paulo state, Brazil	−	Yes	Yes-GC-MS analysis
Lone et al. (2013)	*Euphorbia helioscopia* L	Kashmir valley, India	+	No	No
Kamaraj et al. (2010)	*Azadirachta indica* A. Juss. (=*Melia azedarach* L.)	Amman nagar, dharamapuri district, Tamil nadu, India	+	Yes	No
Iqbal et al. (2006c)	*Swertia chirayita* (Roxb.) H.Karst. (=*Swertia chirata* Buch-Ham)	Procured from local market faisalabad, Pakistan	+	Yes	No
Zhu et al. (2013b)	*Artemisia lancea* Vaniot	Collected from hunan, China	+	Yes	Yes-GC-MS analysis
Idris et al. (1982)	*Artemisia herba-alba* Asso (=*Seriphidium herba-alba* (Asso) Soják)	Not stated	−	Yes	No
Silva et al. (2019)	*Parkia platycephala* Benth	Chapadinha, maranhao, Brazil	−	Yes	No
Qi et al. (2015)	*Zanthoxylum bungeanum* Maxim. (=*Zanthoxylum simulans* Hance)	Collected from hunan, China	+	No	Yes-GC–MS analysis
Katiki et al. (2017b)	Essential oils and other compounds	Grasp Ind. Com. Ltda (curitiba, parana, Brazil)	−	No	Yes-gas chromatography (GC) analysis
Katiki et al. (2017a)	*Terminalia catappa* L	Instituto de zootecnia-nova odessa, sao polo, Brazil	+	Yes	Yes-method not stated
Ferreira et al. (2013)	*Annona muricata* L	Collected from terra de ismael grange located in the municipality of jurucê	−	No	Yes-high performance liquid chromatography and (HPLC) thin-layer chromatography (TLC) analysis
		São paulo, Brazil			
Váradyová et al. (2018)	*Althaea officinalis* L	Commercial sources (AGROKARPATY, plavnica, Slovak republic and BYLINY mikeš s.r.o., cíčenice, Czech republic)	−	No	Yes-liquid chromatography-Mass spectrometry assay
	*Petasites hybridus* (L.) G.Gaertn., B.Mey. and Scherb				
	*Inula helenium* L				
	*Malva sylvestris* L				
	*Foeniculum vulgare* Mill.				
	*Solidago virgaurea* L				
	*Fumaria officinalis* L				
	*Hyssopus officinalis* L				
	*Melisa officinalis* L				
	*Artemisia absinthium* L				
Vargas-Magaña et al. (2014)	*Lysiloma latisiliqum* (L.) Benth.	Faculty of veterinary Medicine-UADY, in mérida, méxico collected from nearby coastal area of mérida, méxico	−	No	No
	*Laguncularia racemose* (L.) C.F. Gaertn				
	*Rhizophora mangle* L				
	*Avicenna germinans* (L.) L				
Mondal et al. (2015)	*Alternanthera sessilis* (L.) R.Br. ex DC.	Gopalgonj, Bangladesh	+	No	Yes-HPLC
Al-Shaibani et al. (2008)	*Justicia adhatoda* L. (=*Adhatoda vasica* Nees)	Harvested from sindh agriculture university (SAU), tandojam, Pakistan	+	Yes	No
Hussien et al. (2011)	*Corriandrum sativum* L	Collected from jimma town and also purchased from local market	+	No	No
	*Thymus schimperi* Ronniger				
	*Ocimum gratissimum* L				
	*Ocimum lamiifolium* Hochst. ex Benth				
	*Ruta chalepensis* L				
	*Echinops kebericho* Mesfin				
Monglo et al. (2006)	*Annona senegalensis* Pers	Northern region of Cameroon	+	Yes	No
	*Terminalia leiocarpa* (DC.) Baill. (=*Anogeissus leiocarpus* (DC) Guil and Perrot.)				
	*Lippia rugosa* A.Chev				
	*Stereospermum kunthianum* Cham.				
	*Vernonia noveboracensis* (L.) Michx. (=*Vernonia tonoteana* L.)				
Ahmed et al. (2020)	*Artemisia herba-alba* Asso (=*Seriphidium herba-alba* (Asso) Soják)	Midaga-tola district	+	No	Yes-method not stated
	*Punica granatum* L				
Acharya et al. (2014)	*Rhus aromatica* Aiton	Collected from South Dakota, North Dakota, Wyoming Montana	+	No	No
	*Ericameria nauseosa* (Pursh) G.L. Nesom and G.I. Baird				
	*Perideridia gairdneri* (Hook. and Arn.) Mathias				
	*Chrysothamnus viscidiflorus* Nutt.				
	*Geranium viscosissimum* Fisch. and C.A. Mey				
	*Melilotus albus* Medik				
	*Liatris punctata* Hook.				
	*Melilotus officinalis* (L.) Lam.				
	*Sanguinaria canadensis* L				
	*Lotus corniculatus* L				
	*Arctostaphylos uva-ursi* (L.) Spreng				
	*Rhus glabra* L				
	*Wyethia sagittata* (Pursh) Mabb				
	(=*Balsamorhiza sagittata* (Pursh) Nutt.)				
	*Enothera* sp				
	*Caltha palustris* L				
	*Cynoglossum officinale* L				
	*Solidago canadensis* L. (=*Solidago mollis* Bartl.)				
	*Centaurea stoebe* L				
	*Glycyrrhiza lepidota* Pursh				
	*Lycopus americanus* Muhl. Ex W.P.C. Barton				
	*Pedicularis racemose* Douglas ex Benth.				
	*Stachys palustris* L				
	*Agastache foeniculum* (Pursh) Kuntze				
	*Pediomelum argophyllum* (Pursh.) J.W.Grimes				
	*Monarda fistulosa* L				
	*Clematis ligusticifolia* Nutt.				
	*Allium cernuum* Roth				
	*Erigeron canadensis* L. (=*Conyza canadensis* (L.) Cronquist)				
	*Cornus Sericea* L				
	*Rubus idaeus* L				
	*Actaea rubra* (Aiton) Willd.				
	*Symphoricarpos occidentalis* (R.Br.) Hook				
	*Artemisia ludoviciana* Nutt.				
	*Artemisia frigida* Willd.				
	*Tanacetum vulgare* L				
	*Cleomella serrulata* (Pursh) Roalson and J.C.Hall				
	(=*Cleome serrulata* Pursh)				
	*Epilobium angustifolium* L				
	*Quercus macrocarpa* Michx.				
	*Salix interior* Rowlee (=*Salix exigua* Nutt.)				
	*Lithospermum molle* (Michx.) Muhl				
	(=*Onosmodium molle* Michx.)				
Cavalcante et al. (2016)	*Calotropis procera* (Aiton.) W.T.Aiton	Universidade estadual do ceará	+	No	Yes-method not stated
Ferreira et al. (2018)	*Citrus × aurantiifolia* (Christm.) Swingle *Anthemis nobilis* L. *Lavandula angustifolia* subsp. *angustifolia* (=*Lavandula officinalis* Chaix)	Purchased from kampo de ervas Ind. and com. Ltda-ME (ribeirão preto, SP, Brazil)	+	Yes	Yes-GC-MS
Fouche et al. (2016)	*Aloe rupestris* Baker	Collected from different locations in South Africa	+	No	No
	*Antizoma angustifolia* (Burch.) Miers ex Harv.				
	*Calpurnia aurea* (Aiton) Benth.				
	*Senna italica* Mill.				
	*Cissus quadrangularis* L				
	*Clematis brachiata* Thunb.				
	*Cleome gynandra* L				
	*Ficus sycomorus* L				
	*Hypoxis rigidula* Baker				
	*Maerua angolensis* DC.				
	*Monsonia angustifolia* E. Mey. Ex A.Rich				
	*Pelargonium luridum* (Andrews) Sweet				
	*Schkuhria pinnata* (lam.) Kuntze ex Thell.				
	*Sclerocarya birrea* (A.Rich.) Hochst				
	*Tabernaemontana elegans* Stapf				
Ahmed et al. (2013)	*Allium sativum* L	Collected from the university of KwaZulu-Natal (UKZN) botanical garden, pietermaritzburg campus, UKZN research farm (ukulinga). *Ficus* spp. were from a private garden (pietermaritzburg) and garlic and ginger samples were purchased from a commercial supermarket.	+	No	No
	*Aloe ferox* Mill				
	*Ananas comosus* (L.) Merr.				
	*Carica papaya* L				
	*Ficus benjamina* L				
	*Ficus ingens* (miq.) Miq				
	*Ficus carica* L				
	*Ficus benghalensis* L. (=*Ficus indica* L.)				
	*Ficus lutea* Vahl				
	*Ficus elastica* Roxb. Ex Hornem				
	*Ficus natalensis* Hochst				
	*Ficus sur* Forssk				
	*Ficus sycomorus* L				
	*Leonotis leonurus* (L.) R.Br				
	*Azadirachta indica* A. Juss. (=*Melia azedarach* L.)				
	*Peltophorum africanum* Sond				
	*Scadoxus puniceus* (L.) Friis and Nordal				
	*Lespedeza cuneata* (Dum. Cours.) G. Don				
	*Tephrosia inandensis* H.M.L. Forbes				
	*Warburgia ugandensis* v				
	*Warburgia salutaris* (G. Bertol.) Chiov.				
	*Cucumis myriocarpus* Naudin				
	*Zingiber officinale* Roscoe				
Iqbal et al. (2001b)	*Allium sativum* L	Not stated	-	No	No
	*Zingiber officinale* Roscoe				
	*Cucurbita ficifolia* Bouché (=*Curcurbita mexicana* Dammann)				
	*Ficus religiosa* L				
Getachew et al. (2012)	*Foeniculum vulgare* Mill	Collected from addis ababa	+	No	No
	*Acokanthera schimperi* (A.DC.) Schweinf				
	*Searsia pyroides* (Burch.) Moffett				
	(=*Rhus vulgaris* Meikle)				
	*Rhus glabra* L				
	*Jasminum abyssinicum* Hochst. ex DC. *Myrsine africana* L				
Zamilpa et al. (2019)	*Dysphania ambrosioides* (L.) *Mosyakin and Clemants* (=*Chenopodium ambrosioides* L.) *Castela tortuosa* Liebm	Acquired at a local market in the town left of cuernavaca city, morelos, Mexico	+	Yes	No
Iqbal et al. (2005)	*Calotropis procera* (Aiton) W.T.Aiton	Cholistan rangeland, district bahawalpur (Pakistan)	+	Yes	No
Jabbar et al. (2007)	*Chenopodium album* L	Procured from local market in faisalabad (Pakistan)	+	No	No
	*Caesalpinia crista* L				
Kamaraj and Rahuman (2011)	*Annona squamosa* L	Collected from the tropical region hills, Tamil nadu, India	+	No	Yes-lieberman–Burchard test
	*Eclipta prostrata* (L.) L				
	*Solanum torvum* Sw				
	*Terminalia chebula* Retz				
	*Catharanthus roseus* (L.) G. Don				
Kamaraj et al. (2011)	*Andrographis paniculata* (Burm.f.) Nees	Collected from javadhu hills, tiruvannamalai district and dharmapuri district Tamil nadu, India	-	Yes	No
	*Anisomeles malabarica* (L.) Kuntze (=*Anisomeles malabarica* (L.) R.Br.)				
	*Annona squamosa* L				
	*Datura metel* L				
	*Solanum torvum* Sw				
Marie-Magdeleine et al. (2010)	*Tabernaemontana citrifolia* L	Collected in Guadeloupe, French west indies	-	No	Yes-TLC
Nery et al. (2010)	*Anacardium humile* A.St.-Hil	Collected in the cerrado of a rural region of montes claros city, Brazil	+	No	Yes-phytochemical analysis
Veerakumari and Chitra (2016)	*Allium sativum* L	Not stated	-	Yes	No
de Oliveira et al. (2011)	*Myracrodruon urundeuva* Allemao	Collected in December 2009 in fortaleza, Ceará,Brazil	+	Yes	Yes-method not stated
Malik et al. (2019)	*Artemisia vulgaris* L	Collected from botanical garden at the federal university of maranhão, sao luís, maranhão, Brazil	+	No	Yes-GC-MS analysis
Soares et al. (2018)	*Myracrodruon urundeuva* Allemao	Purchased from arboleft seed trade (birigui, sao paulo, Brazil	+	No	Yes-proteomic analysis (LC-ESI-MS/MS)
Tariq et al. (2009)	*Artemisia absinthium* L	Collected from the aharbal area of southern kashmir valley	+	No	No
Zhu et al. (2013a)	*Arisaema franchetianum* Engl.	Collected from yunnan province, China	+	No	Yes-GC–MS analysis
	*Arisaema lobatum* Engl.				
Carvalho et al. (2012)	*Lippia origanoides* Kunth (=*Lippia sidoides* Cham.)	Institute of chemistry of paulista state	-	No	Yes- GC-MS analysis
	*Mentha × piperita* L	Embrapa western. Amazon research station, Brazil			
	*Hura crepitans* L	Acquired in the local market of porto velho			
	*Couroupita guianensis* Aubl.				
Katiki et al. (2012)	*Cymbopogon schoenanthus* (L.) Spreng	Purchased from WNF Ind. and com. Ltda (sao Paulo-SP, Brazil)	-	No	Yes- GC analysis
Macedo et al. (2012)	*Lantana camara* L	Collected in the horto of medicinal plants of the universidade federal do ceará in plots, state of ceará, Brazil	+	No	Yes-GC analysis
	*Alpinia zerumbet* (Pers.) B.L.Burtt and R.M.Sm				
	*Mentha arvensis* L. (=*Mentha villosa* Becker)				
	*Tagetes minuta* L				
Ribeiro et al. (2014)	*Corymbia citriodora* (Hook.) K.D.Hill and L.A.S.Johnson (=*Eucalyptus citriodora* Hook.)	Purchased from FERQUIMA (vargem grande paulista, são paulo, Brazil)	-	No	Yes- infrared spectroscopy (FTIR)
Andre et al. (2016)	Carvacrol	Obtained via the acetylation of carvacrol (Sigma–Aldrich^®^, st. Louis, United States	-	No	Yes-FTIR
Morais-Costa et al. (2016)	*Lachesiodendron viridiflorum* (Kunth) P.G.Ribeiro, L.P.Queiroz and Luckow (=*Piptadenia viridiflora* (Kunth) Benth.)	Cerrado vegetationnear montes claros city in north minas gerais state, Brazil	-	Yes	Yes-HPLC-DAD
Féboli et al. (2016)	*Opuntia ficus-indica* (L.) Mill.	Collected in the municipality of ilha solteirain the state of são paulo, Brazil in September 2014	-	No	No
Tadesse et al. (2009)	*Maesa lanceolate* Forssk	Collected along the addis ababa-butajira road and *Maesa laceolata*	+	No	No
	*Coleus maculosus* subsp. Maculosus (=*Plectranthus punctatus* (L.f.) L’Her.)				
Herath et al. (2019)	*Cryptocarya massoy* (Oken) Kosterm. (=*Cryptocarya ovoguineensis* Teschner)	Not stated	-	Yes	Yes-HPLC
	*Piper methysticum* G.Forst				
Gaínza et al. (2015)	*Citrus × aurantium* L. (=*Citrus × sinensis* (L.) Osbeck)	Not stated	−	Yes	Yes-GC–MS analysis
	*Melaleuca quinquenervia* (Cav.) S.T.Blake				
de Araújo-Filho et al. (2018)	*Corymbia citriodora* (Hook.) K.D.Hill and L.A.S.Johnson (=*Eucalyptus citriodora* hook.)	Purchased from ferquima (são paulo, Brazil)	−	Yes	Yes-GC–MS analysis
Hördegen et al. (2006)	*Azadirachta indica* A. Juss	Obtained from alfred galke GmbH, gittelde (Germany). Purchased from S.V.S. Medicinal crops dealers pvt. Ltd., guntur (India)	−	No	No
	*Caesalpinia crista* L				
	*Fumaria parviflora* Lam				
	*Embelia ribes* Burm. f				
	*Baccharoides anthelmintica* (L.) Moench (=*Vernonia anthelmintica* (L.) Willd.)				
	*Ananas comosus* (L.) Merr.				
Camurça-Vasconcelos et al. (2007)	*Croton* grewioides Baill. (=*Croton zehntneri* Pax and K.Hoffm.)	Collected in vicosa, ceara state, Brazil	+	No	Yes-GC–MS analysis
	*Lippia origanoides* Kunth. (=*Lippia sidoides* Cham.)	Purchased from PRONAT (produtos naturais) in the state of ceara			
Hernández-Villegas et al. (2011)	*Phytolacca icosandra* L	Collected in yaxcabá, yucatan, Mexico	+	No	Yes-method not stated
Eguale et al. (2011)	*Croton macrostachyus* Hochst. ex Delile	Collected from their natural habitat	+	No	Yes-method not stated
	*Ekebergia capensis* Sparrm.				
	*Vachellia nilotica* (L.) P.J.H.Hurter and Mabb. (=*Acacia nilotica* (L.) willd. Ex. Delile)				
	*Terminalia schimperiana* Hochst. (=*Terminalia glaucescens* Planch. ex Benth.)				
Hamad et al. (2014)	*Nicotiana tabacum* L	Purchased from the local market of faisalabad, Pakistan	−	Yes	No
	*Azadirachta indica* A. Juss				
De Jesús-Gabino et al. (2010)	*Prosopis laevigata* (Humb. and Bonpl. Ex Willd.) M.C.Johnst	Collected from the sierra de huautla, ecological reserve of the biosphere, in morelos state, Mexico	−	No	No
Tariq et al. (2008)	*Achillea millefolium* L	Collected from the aharbal area of southern kashmir valley	+	No	No
Piza et al. (2019)	*Psidium cattleyanum* Sabine	Glaucilândia, Brazil	+	Yes	No
André et al. (2020)	Carvacryl acetate	Not stated	−	Yes	Yes-GC- MS analysis
Macedo et al. (2019)	*Cymbopogon citratus* (DC.) Stapf	Not stated	−	Yes	Yes-GC-MS analysis
Araújo-Filho et al. (2019)	*Corymbia citriodora* (Hook.) K.D.Hill and L.A.S.Johnson (=*Eucalyptus citriodora* Hook.)	Not stated	−	Yes	Yes-GC-MS analysis
Iqbal et al. (2001a)	*Sorghum bicolor* (L.) Moench	Not stated	−	Yes	No
Lara TF et al. (2009)	*Eucalyptus globulus* Labill.	Not stated	−	Yes	Yes-GC-MS analysis
Ademola et al. (2004)	*Khaya senegalensis* (Desv.) A. Juss	Ibadan, Nigeria	+	Yes	No
Ademola et al. (2007)	*Spigelia anthelmia* L	Ibadan, Nigeria	+	Yes	No
Alowanou et al. (2019)	*Bridelia ferruginea* Benth	Abomey-calavi, kandi and comé, Benin	+	Yes	No
	*Combretum glutinosum* Perr. ExDC.				
	*Mitragyna inermis* (Willd.) Kuntz				
Al-Qarawi et al. (2001)	*Calotropis procera* (Aiton.) W.T.Aiton	Purchased from local market in almoznib, king saud university at buraydah	−	Yes	No
Iqbal et al. (2006a)	*Baccharoides anthelmintica* (L.) Moench	Faisalabad, Punjab	+	Yes	No
	(=*Vernonia anthelmintica* (L.) Willd.)				
Ademola and Idowu (2006)	*Leucaena leococephala* (Lam.) de Wit	Ibadan, Nigeria	+	Yes	No
Barone et al. (2018)	*Vaccinium macrocarpon* Aiton	Not stated	−	Yes	Yes-Mass spectra method
Castillo-Mitre et al. (2017)	*Vachellia campeachiana* (Mill.) Seigler and Ebinger (=*Acacia cochliacantha* humb. and bonpl. Ex willd.)	Salitre palmarillos village, Mexico	+	Yes	Yes-Mass spectrometry and HPLC analysis
Domingues et al. (2013)	*Ananas comosus* (L.) Merr.	São paulo state, Brazil	+	Yes	No
Dixit et al. (2019)	*Annona squamosa* L	Local market, jabalpur India	−	Yes	No
	*Azadirachta indica* A. Juss				
	*Nicotiana tabacum* L				
Ferreira et al. (2016)	*Thymus vulgaris* L	Ferquima Ind e com ltda (Brazil)	+	Yes	Yes-GC-MS analysis
Githiori et al. (2004)	*Hagenia abyssinica* (Bruce) J.F.Gmel	Kenya and east africa	−	Yes	No
	*Dodonaea viscosa* subsp. *angustifolia* (L.f.) J.G.West (=*Dodonaea angustifolia* L.f.)				
	*Olea europaea* L				
	*Annona squamosa* L				
	*Hildebrandtia sepalosa* Rendle				
	*Azadirachta indica* A. Juss				
	*Ananas comosus* (L.) Merr.				
Heckendorn et al. (2006)	*Onobrychis viciifolia* Scop	Not stated	−	Yes	No
Palacios-Landín et al. (2015)	*Allium sativum* L	Local market of the city of cuernavaca and rural area of tixtla in the state of morelos, Mexico	−	Yes	No
	*Tagetes erecta* L				
Hassan et al. (2019)	*Abutilon theophrasti* Medik	“Lower-munda” District, Qazigund,India	+	Yes	No
Iqbal et al. (2004)	*Seriphidium brevifolium* (Wall. ex DC.) Ling and Y.R.Ling (=*Artemisia brevifolia* Wall. ex. DC.)	Faisalabad, Pakistan	+	Yes	No
Cabardo and Portugaliza (2017)	*Moringa oleifera* Lam	Collected from brgy. Sto. Rosario, baybay city, leyte	-	No	No
Assis et al. (2003)	*Spigelia anthelmia* L	Not stated	−	No	No
Hajaji et al. (2018)	*Matricaria recutita* L	Obtained from beja, north-west of tunitia	−	Yes	Yes-method not stated
Costa et al. (2008)	*Azadirachta indica* A. Juss	Collected in eusebio, ceara, located in northeast Brazil	+	No	Yes-method not stated
Cortes-Morales et al. (2019)	*Baccharis conferta* Kunth	Collected from the iztaccíhuatl- popocatépetl national park	+	Yes	Yes-TLC
Eguale et al. (2007a)	*Hedera helix* L	Collected from addis ababa, Ethiopia	+	Yes	Yes-method not stated
Eguale et al. (2007b)	*Coriandrum sativum* L	Purchased from debre birhan, Ethiopia	+	Yes	Yes-method not stated
Oliveira et al. (2014)	*Piper aduncum* L	Galley forest of the angico river, bocaiúva site, minas gerais state, Brazil	+	Yes	Yes-GC analysis
Karim et al. (2019)	*Artemisia vulgaris* L	BAU campus	−	Yes	No

### Taxonomic Clarification

According to the modern botanical nomenclature, 53 reported plant species have a synonym issue. Plant accepted names are mentioned in table (Table 1; Supplementary Tables S2, S3), while the synonym mentioned in the original articles were put into brackets. In addition, taxonomic corrections regarding the author of those plants and their family names were also revised.

### Pharmacological Validation of Medicinal Plants Against *H. contortus*

Total of 187 plant species belonging to 59 families and 145 genera were tested against different life stages of *H. contortus* (Supplementary Tables S2, S3). Major contributed families with their species were Asteraceae (*n* = 29), Fabaceae (*n* = 19), Lamiaceae (*n* = 12), and Euphorbiaceae (*n* = 6). Different life forms of plants reported were herbs, trees, and shrubs 40, 31.4, and 27% in accordance of their order. Leaves were the most frequently used part (50%) followed by seeds (11.3%), roots (8%), and whole plants (7%). Among other plant parts stems, flowers, barks, shoots, fruits, bulbs, peel, fibers, pulp, and latex were included (Supplementary Table S2).

**FIGURE 2:**
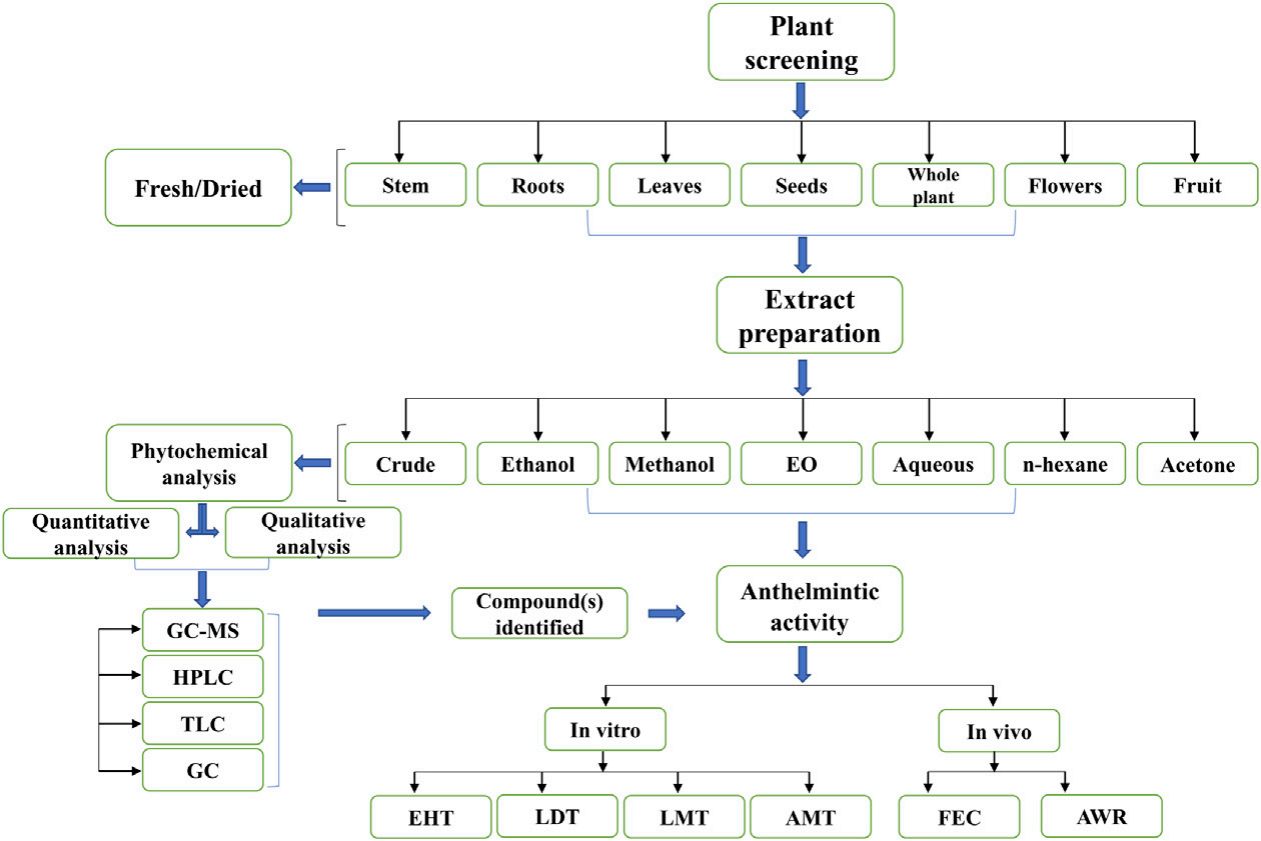
Selection and processing of plant material(s) for anthelmintic evaluation.

Different solvents including n-hexane, aqueous, ethanolic, hydro-alcoholic, methanolic, and others were used in extracts preparation (Figure 2). Among all, the methanol extract was the predominant one. Bioassays reported in this review included, egg hatching test (EHT), larval development test (LDT), larval motility test (LMT), adult worm motility test (AWMT), adult parasite mortality test (APMT), and larval artificial exsheathment assay (LAEA) in *in vitro* studies, while fecal egg count reduction (FECR), egg count per gram of feces (EPG), total warm count reduction (TWC/WCR) in *in vivo* studies. Furthermore, among the above mentioned assays, EHT was the most widely used assay for evaluation of medicinal plants against *H. contortus* *in vitro*, while FECR was common *in vivo*. Sheep were the commonly used experimental model *in vivo* (Supplementary Table S3).

**FIGURE 3:**
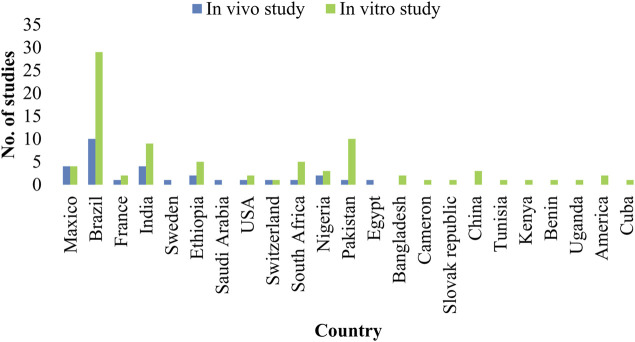
Country-wise comparison of *in vitro* and *in vivo* studies.

**FIGURE 4:**
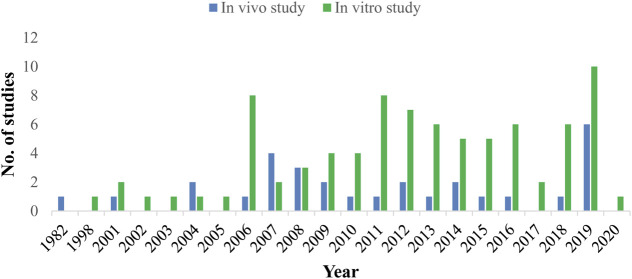
Year-wise comparison of *in vitro* and *in vivo* studies.

Out of the total reported plant species 171 plant species were evaluated *in vitro* and only 40 plant species *in vivo* against *H. contortus*. It is evident that *in vitro* studies were almost five times greater than *in vivo* studies. Mostly, *in vitro* studies were carried out in Brazil (*n* = 29 studies), then in Pakistan (*n* = 10 studies), and India (*n* = 9 studies) among others. Similarly, *in vivo* studies were also mostly reported from Brazil and India, 10 and 4 studies, respectively, (Figure 3). Most of the pharmacological studies were reported in the year 2019 (*n* = 15) and 2006, 2011, 2012 (*n* = 9) in each (Figure 4).

### Comparative Analysis and Toxicity of Common Plant Species

Plant species, which were common in both *in vitro* and *in vivo* studies, were compared to discriminate their efficacy against *H. contortus*. A total of 24 plant species were found to be commonly used both *in vitro* and *in vivo* (Table 2).

**TABLE 2:** Comparative analysis, toxicology, and mechanism of action of medicinal plants.

Plant names	*In vitro*			*In vivo*			Toxicology		Mechanism of action	References
	Ext	Conc. (mg/ml)	Eff. (%)	Ext	Dos. (mg/kg)	Eff. (%)	Dos. (mg/kg)	T. level		
*Allium sativum* L	Ethanol	0.5	88.5–100	n-hexane	40	68.7	NA	Moderate	Destructive and inhibitive effect on acetylcholinesterase causing paralysis	Krstin et al. (2018); Palacios-Landín et al. (2015); Veerakumari and Chitra (2016)
*Annona squamosa* L	Methanol	25	100	Aqueous	1500	No effect	NA	NA	NA	Dixit et al. (2019); Kamaraj et al. (2011)
*Achillea millefolium* L	Crude aqueous	25	94.4	Crude aqueous	2	88.4	0.01	Nil	Alteration of cell shape, restrict growth, collapsing cell membrane, and arrest cell division	Cavalcanti et al. (2006); Tariq et al. (2008)
							0.003			
*Artemisia absinthium* L	Methanol	1.024	100	Crude ethanol	2000	90.46	NA	Toxic	Inhibit vital metabolic enzymes, disrupt mitochondrial membrane potential, release of cytochrome c into cytoplasm and activation of caspase-3-mediated apoptosis	Lachenmeier (2010); Tariq et al. (2009); Váradyová et al. (2018)
		25	85							
*Artemisia herba-alba* Asso	Methanol	1.25–10	100	NA	0.003	100	300	Toxic	NA	Ahmed et al. (2020); Almasad et al. (2007); Idris et al. (1982)
*Artemisia capillaris* Thunb. (=*Artemisia vestita* Wall. ex Besser)	Methanol	50	95	Methanol	50	86.35	NA	Nil	NA	Irum et al. (2015)
		25	100							
*Artemisia maritima* L.(=*Seriphidium maritimum* (L.) Poljakov	Methanol	25	100	Methanol	50	82.22	NA	Nil	NA	Irum et al. (2015)
*Ananas comosus* (L.) Merr.	Ethanol	200	100	Aqueous	2000	No effect	750	Nil	Remove and digest the cuticle layer causing immobility and death	Ahmed et al. (2013); Domingues et al. (2013); Maurer (2001)
							5000			
*Azadirachta indica* A. Juss	Aqueous	12.5	97.8	Aqueous	4000	85.24	18.4–45	Toxic	Inhibit secretion of key enzymes, intracellular instability, neuromuscular disorganization, paralysis, and death	Deng et al. (2013); Hamad et al. (2014); Kamaraj et al. (2010); Maciel et al. (2006)
	Hydro-alcoholic	12.5	98.4	Methano						
	Ethanol	50	100	l						
*Calotropis procera* (Aiton) W.T.Aiton	Aqueous	25	70	Aqueous	0.003	88.4	0.001–6	Toxic	Tegumental disorganization and paralysis	Cavalcante et al. (2016); Iqbal et al. (2005); Mahmoud et al. (1979)
	Ethyl acetate	4	91							
*Castela tortuosa* Liebm.	n-hexane	40	70	n-hexane	40	27.1	NA	NA	NA	(Zamilpa et al., 2019)
*Coriandrum sativum* L	Essential oil	10	88.63	Aqueous	450	25	1000–5000	Nil	Inhibit vital functions, interfere metabolic processes, and destruction of nervous system	Eguale et al. (2007b); Hussien et al. (2011); Patel et al. (2012)
*Cocos nucifera* L	Ethyl acetate	5	100	Ethyl acetate	400	Not effective	2000	Toxic	Induces chemical and physical damage by binding to proteins of cuticle, oral cavity, esophagus, and cloaca	Oliveira et al. (2009); Tayler et al. (2020)
		80	99.77							
*Corymbia citriodora* (Hook.) K.D.Hill and L.A.S. Johnson	Essential oil	2	100	Essential oil	0.125, 0.25, 0.5	100	5000	Moderate	Formation of vacuoles, muscular disorganization, and changes in mitochondrial profile	Araújo-Filho et al. (2019); Ribeiro et al. (2014)
		8	100							
*Cymbopogon citratus* (DC.) Stapf	Essential oil	1.25	98.4	Essential oil	50	23.9	31.2 μg/ml	Nil	Alter the permeability, depolarization of membrane, and disrupt lipids, polysaccharides, and phospholipids	Macedo et al. (2019); Santoro et al. (2007)
	Essential oil nano-emulsion		97.1							
*Cymbopogon schoenanthus* (L.) Spreng.	Essential oil	0.18	98.6	Essential oil	360	No activity	180; 360	Nil	Inhibit vital functions, interfere metabolic processes, and destruction of nervous system	Katiki et al. (2012)
			96.8							
*Eucalyptus staigeriana* F. Muell. Ex F.M. Bailey	Essential oil	1.75	100	Essential oil	500	46.44	1000–5000	Nil	Alter the permeability, depolarization of membrane, and disrupt lipids, polysaccharides, and phospholipids	Macedo et al. (2010); Ribeiro et al. (2014)
							200–600			
*Euphorbia helioscopia* L	Methanol	25	98	Methanol	5	86	2000	Nil	Induce expansion, increase permeability, and disturb chemical structure of membrane	Lone et al. (2012); Saleem et al. (2016)
			82							
*Lippia origanoides* Kunth (=*Lippia sidoides* cham.)	Essential oil	0.625	100	Essential oil	283	56.9	NA	Moderate	Inhibit vital functions, interfere metabolic processes, nervous system destruction	Camurça-Vasconcelos et al. (2007); de Melo et al. (2020)
*Lysiloma latisiliquum* (L.) Benth.	Acetone	3.6	Not effective	NA	0.8	Highly effective	NA	NA	Cytoplasmic vacuolization, disturb muscular cells, and tissues	Martínez-Ortiz-de-Montellano et al. (2019); Vargas-Magaña et al. (2014)
*Nicotiana tabacum* L	Aqueous	25	77	Aqueous	4000	86.6	5000	Nil	Inhibit acetylcholine, and paralysis	Andjani et al. (2019); Hamad et al. (2014); Iqbal et al. (2006b)
	Methanol			Methano						
*Lachesiodendron viridiflorum* (Kunth) P.G.Ribeiro, L.P.Queiroz and Luckow (=*Piptadenia viridiflora* (Kunth) Benth.)	Aqueous	0.075	100	Aqueous	283	Highly effective	203	Nil	Inhibit enzymatic activities and metabolic processes	Morais-Costa et al. (2016)
	Methanol									
*Seriphidium brevifolium* (Wall. Ex DC.) Ling and Y.R.Ling	Methanol	25	80	Aqueous	0.003	67.2	NA	NA	NA	Iqbal et al. (2004)
*Spigelia anthelmia* L	Ethyl acetate	50	Highly effective	Aqueous	500	Significantly effective	5000	Nil	Destruct cuticle layer, degrade egg membrane and chitin of egg shell, inhibit development and death	Ademola et al. (2007); Assis et al. (2003); Ribeiro et al. (2017)
	Methanol									

NA, Data not available.

After comparative analysis based on minimum concentration and maximum efficacy, the identified plant species with promising anthelmintic activity *in vitro* against *H. contortus* were *Lachesiodendron viridiflorum* (Kunth) P.G.Ribeiro, L.P.Queiroz and Luckow (syn. *Piptadenia viridiflora* (Kunth) Benth.)*, Cymbopogon schoenanthus* (L.) Spreng.*, Allium sativum* L.*,* *Lippia origanoides* Kunth (syn. *Lippia sidoides* Chem.)*, Artemisia absinthium* L.*, Cymbopogon citratus* (DC.) Stapf*, Eucalyptus staigeriana* F. Muell. ex F.M. Bailey*, Artemisia herba-alba* Asso*,* and *Corymbia citriodora* (Hook.) K.D. Hill and L.A.S. Johnson in order of their appearance. While *A. herba-alba*, *C. procera*, *C. citriodora*, *Lysiloma latisiliqum* (L.) Benth.*,* and *Euphorbia helioscopia* L. were reported with high efficacy *in vivo*. Among the plants, *L. viridiflorum* was found highly effective both *in vitro* and *in vivo* with no observed toxic effects. *C. citriodora* was moderately toxic *in vivo*, but with promising nematicidal activity *in vitro* and *in vivo*. Additionally, *C. procera* and *A. herba-alba* despite of their high anti-haemonchiasis activity were found to be highly toxic at the tested concentrations (Supplementary Table S4). However, five species namely; *Annona squamosa* L., *Artemisia maritima* L., *Artemisia capillaris* Thunb., *Castela tortuosa* Liebm.*, L. latisiliquum*, and *Seriphidium brevifolium* (Wall. ex DC.) Ling and Y.R. Ling were not evaluated for their toxicological effects. Mostly non-toxic and low-toxic extracts were orally administered. The LC_50_ value of most plant species was missing and was not calculated.

From the results, it is evident that the effects of different plant extracts were dose and time dependent. Moreover, the comparative analysis also revealed that plant species were more effective *in vitro* than *in vivo* against various stages of the parasite.

### Phyto-Compounds With Anti-Haemonchiasis Activity

In this review, 19 compounds were reported to be assessed for *in vitro* activity against different life stages of *H. contortus*. While only 3 compounds were found to be evaluated for *in vivo* activity using gerbil (*Meriones unguiculatus*) as an animal model. Based on minimum concentration and maximum nematicidal activity, Cinnamaldehyde, anethole, and carvone were highly active and completely inhibited egg hatching of the parasite at the tested concentrations of 0.085, 0.085, and 0.366 mg/ml, respectively. Carvacrol inhibited the larval development at a minimum concentration of 1 mg/ml, though the effectiveness of thymol and anethole against larvae development was also significant, but relatively at high concentrations i.e., 10 and 20 mg/ml, correspondingly.

Carvacrol and carvacryl acetate at 2 mg/ml showed 100% larval motility inhibition as compared to other compounds. Similarly, carvacryl acetate (0.2 mg/ml) and citronellal (2 mg/ml) totally reduced the motility of adult parasites *in vitro*. However, citronellal was moderately toxic in mice. Anethole and carvone significantly reduced fecal egg count, decreased male length, and reproductive capacity of female at 50 mg/kg concentration *in vivo* (Table 3).

**TABLE 3:** Plant compounds efficacy against *H. contortus*.

Compound name	IUPAC name	Chemical structure	Plant name/family	Extract	Concentration (mg/ml)	Assays	Inhibition (%)	References
1,8-Cineole	1,3,3-Trimethyl-2-oxabicyclo [2.2.2] octan	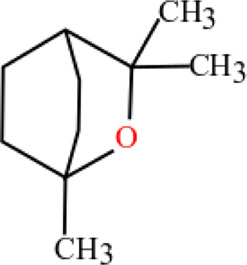	*Artemisia lancea* Vaniot/Asteraceae	Essential oil	0.63	EHT	3.4	Zhu et al. (2013b)
					1.25		10.0	
					2.5		26.6	
					5.0		56.6	
					10.0		74.8	
					0.63	LDT	10.6	
					1.25		23.4	
					2.5		33.6	
					5.0		49.2	
					10.0		65.2	
					0.63	LMT	5.5	
					1.25		10.4	
					2.5		30.2	
					5.0		48.6	
					10.0		60.3	
			Produced synthetically	Essential oil	1,787	EHT	99	Katiki et al. (2017b)
Anethole	1-Methoxy-4-(prop-1-en-1-yl)benzene	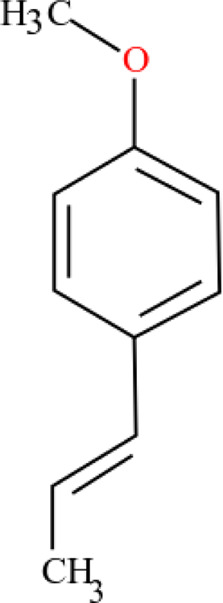	*Croton zehntneri* Pax and K.Hoffm./Euphorbiaceae	Essential oil	0.31	EHT	6.7	Camurça-Vasconcelos et al. (2007)
					0.62		26.6	
					1.25		99.9	
					1.25	LDT	35.8	
					2.5		52.1	
					5.0		87.7	
					10.0		96.7	
					20.0		100	
			Supplied by GRASP Ind. E com. (Curitibia-PR, Brazil)	Encapsulated oil	50	FEC^*^	Fecal egg count was significantly reduced, decreased male length, and reproductive capacity of female after 45 days in santa ines lambs	Katiki et al. (2019)
					20		The dose did not affect acquisition of parasites after pasture access and as FEC raised and body weight decreased of morada nova lambs	
			Produced synthetically	Essential oil	0.085	EHT	99	Katiki et al. (2017b)
Borneol	1,7,7-Trimethylbicyclo [2.2.1]heptan-2-ol	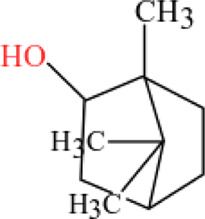	*Zanthoxylum bungeanum* Maxim. (=*Zanthoxylum simulans* Hance)/Rutaceae	Essential oil	1.25	EHT	48.6	Qi et al. (2015)
					2.5		62.1	
					5.0		80.0	
					10.0		92.2	
					20.0		98.8	
					40.0		100	
					1.25	LDT	38.2	
					2.5		52.2	
					5.0		80.2	
					10.0		91.2	
					20.0		98.0	
					40.0		100	
					1.25	LMT	52.8	
					2.5		37.2	
					5.0		23.2	
					10.0		9.6	
					20.0		2.6	
					40.0		1.8	
Camphor	1,7,7-trimethylbicyclo [2.2.1]heptan-2-one	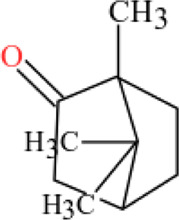	*Artemisia lancea* Vaniot/Asteraceae	Essential oil	0.63	EHT	-	Zhu et al. (2013b)
					1.25		-	
					2.5		2.8	
					5.0		9.0	
					10.0		12.8	
					0.63	LDT	4.4	
					1.25		15.8	
					2.5		23.4	
					5.0		37.2	
					10.0		57.0	
					0.63	LMT	5.7	
					1.25		13.2	
					2.5		17.4	
					5.0		13.0	
					10.0		18.1	
Carvacrol	2-Methyl-5-(propan-2-yl)phenol	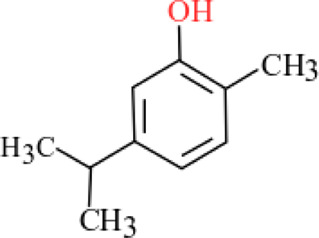	*Arisaema franchetianum* Engl.*, A. lobatum* Engl.*/*Araceae	Essential oil	0.32	EHT	52.6	Zhu et al. (2013a)
					0.63		65.6	
					1.25		83.4	
					2.5		93.2	
					5.0		100	
					10.0		100	
					0.32	LDT	38.4	
					0.63		54.2	
					1.25		77.2	
					2.5		89.2	
					5.0		98.0	
					10.0		100	
			Produced synthetically	Essential oil	5.517	EHT	99	Katiki et al. (2017b)
				Essential oil	1	EHT	97.7	Andre et al. (2016)
					2	LMT	100	
					0.2	AWMT	58.3	
Carvacryl acetate^*^	Phenol, 2-methyl-5-(1-Methylethyl)-, Acetate	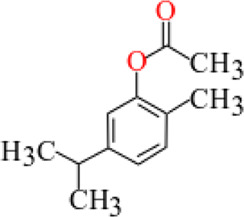	NA	NA	250	EGP	The compound reduced 57.7% of eggs per Gram of gastrointestinal parasites including *H. contortus*	André et al. (2020)
				Essential oil	8	EHT	89.3	Andre et al. (2016)
					2	LMT	100	
					0.2	AWMT	100	
Eugenol	4-Allyl-2-methoxyphenol	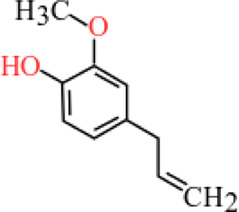	*Ocimum gratissimum* L./Lamiaceae	Essential oil	0.625	EHT	58.49	Pessoa et al. (2002)
					1.25		76.02	
					2.5		94.56	
					5		100	
					10		100	
			Produced synthetically	Essential oil	51.65	EHT	99	Katiki et al. (2017b)
Linalool	3,7-Dimethyl-1,6-octadien-3-ol	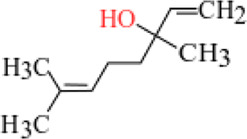	*Arisaema franchetianum* Engl.*, A. lobatum* Engl.*/*Araceae	Essential oil	0.32	EHT	2.0	Zhu et al. (2013a)
					0.63		6.4	
					1.25		12.6	
					2.5		29.6	
					5.0		46.6	
					10.0		65.8	
					0.32	LDT	1.8	
					0.63		4.4	
					1.25		12.0	
					2.5		25.2	
					5.0		37.6	
					10.0		48.2	
			Produced synthetically	Essential oil	17.47	EHT	99	Katiki et al. (2017b)
Thymol	5-Methyl-2-(propan-2-yl)phenol	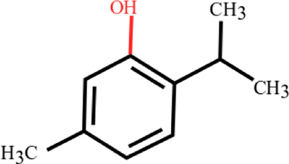	*Lippia origanoides* Kunth (=*Lippia sidoides* Cham.)/Verbenaceae	Essential oil	0.31	EHT	9.9	Camurça-Vasconcelos et al. (2007)
					0.62		93.6	
					1.25		98.2	
					1.25	LDT	33.0	
					2.5		54.8	
					5.0		73.9	
					10.0		99.2	
					20.0		99.7	
			Produced synthetically	Essential oil	5.0	EHT	99	Katiki et al. (2017b)
Citronellal	3,7-Dimethyloct-6-enal	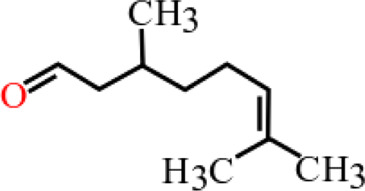	*Corymbia* *citriodora* (Hook.) K.D. Hill and L.A.S. Johnson (=*Eucalyptus citriodora* Hook.)/Myrtaceae	Essential oil	0.75	AWMT	37.50	Araújo-Filho et al. (2019)
					1		54.16	
					1.25		70.83	
					1.5		83.33	
					1.75		95.83	
					2		100	
Lectin	9-Benzyl-3-methylidene-1,5-bis-(4-methylphenyl)sulfonyl-1,5,9-triazacyclododecane	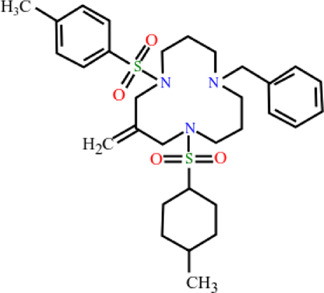	*Parkia platycephala* Benth/Leguminosae	Protein	1.2	LDT	-	Silva et al. (2019)
					LET	-		
					0.31	LDT	50	
Goniothalamin	(2*R*)-2-[(*E*)-2-phenylethenyl]-2,3-dihydropyran-6-one	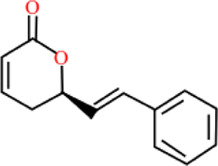	*Cryptocarya* *massoy* (Oken) Kosterm. (=*Cryptocarya novoguineensis* Teschner)*/*Lauraceae	NA	200–300 μM	LDT	IC_50_	Herath et al. (2019)
					6.25 μM	LMT	IC_50_	
Dihydrokavain	(2 S)-4-methoxy-2-(2-phenylethyl)-2,3-dihydropyran-6-one	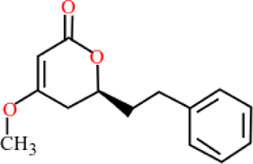	*Piper methysticum* G. Forst.*/*Piperaceae	NA	207 μM	LDT	IC_50_	Herath et al. (2019)
						LMT	-	
Desmethoxyyangonin	4-Methoxy-6-[(E)-2-phenylethenyl]pyran-2-one	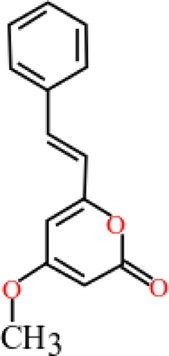	*P. methysticum* G. Forst.*/*Piperaceae	NA	31.7 μM	LDT	IC_50_	Herath et al. (2019)
						LMT	-	
Yangonin	4-Methoxy-6-[(E)-2-(4-methoxyphenyl) ethenyl] pyran-2-one	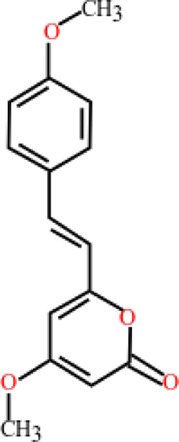	*P. methysticum* G. Forst.*/*Piperaceae	NA	23.7 μM	LDT	IC_50_	Herath et al. (2019)
						LMT	-	
Carvone^*^	2-Methyl-5-prop-1-en-2-ylcyclohex-2-en-1-one	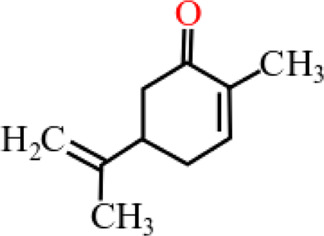	Supplied by GRASP Ind. E com. (Curitibia-PR, Brazil)	Encapsulated oil	50	FEC	Fecal egg count was significantly reduced, decreased male and reproductive capacity of female after 45 days in santa ines lambs	Katiki et al. (2019)
					20		The dose did not affect acquisition of parasites after pasture access and as FEC raised and body weight decreased of morada nova lambs	
			Produced synthetically	Essential oil	0.366	EHT	99	Katiki et al. (2017b)
Cinnamaldehyde	(E)-3-phenylprop-2-enal	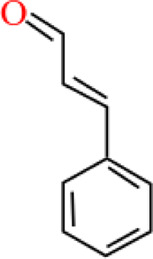	Produced synthetically	Essential oil	0.085	EHT	99	Katiki et al. (2017b)
Vanillin	4-Hydroxy-3-methoxybenzaldehyde	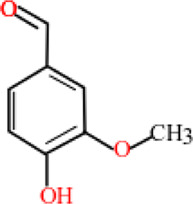	Produced synthetically	Essential oil	815.16	EHT	99	Katiki et al. (2017b)
Limonene	1-Methyl-4-prop-1-en-2-ylcyclohexene	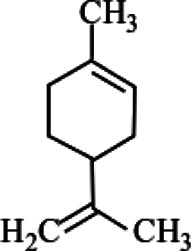	Obtained from citrus peel	Essential oil	207.56	EHT	50	Katiki et al. (2017b)

AWMT, Adult Worm Motility Test; LET, Larval Exsheathment Test; LMT, Larval Motility Test.

### Quantitative Analysis

#### Jaccard Similarity Index

**FIGURE 5:**
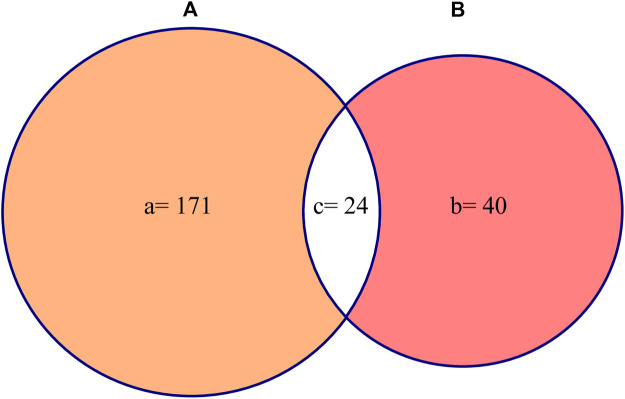
Jaccard similarity index **(A)** represents plants used *in vitro* and **(B)** represents plants used *in vivo*.

The JI was used to compare the two sets of data (i.e., *in vitro* and *in vivo* studies) to determine the similarity of the studies reported in this review. The result revealed 12.8% similarity between the two data sets (Figure 5).

## Discussion

### Anti-haemonchiasis Medicinal Plants, Their Families, and Habit

Extensive use of Asteraceae, Fabaceae, Lamiaceae, and Euphorbiaceae, users’ reliability, and antiparasitic activity could be attributed to the presence of potent phytochemicals such as saponins, tannins, flavonoids in Asteraceae (Carvalho et al., 2013), saponins, essential oils in Lamiaceae (Raja, 2012), phenolic compounds, alkaloids, and hemagglutinins in Fabaceae (Żarnowski et al., 2001), and alkaloids and terpenoids in Euphorbiaceae (Mwine and Van Damme, 2011). Mechanism of action of these compounds against different developmental stages of *H. contortus* is still unknown, however, probably these may inhibit both egg embryonation and direct effects on the larvae (Al-Shaibani et al., 2008). Athanasiadou et al. (2001) attributed the antiparasitic activity of condensed tannins to their ability to bind with the cellular membrane proteins, which results in the unavailability of nutrients to the larvae, causing starvation, and death (Athanasiadou et al., 2001). It seems that complexities of these compounds enable them to interrupt various molecular targets of different developmental stages of the parasite. Saponoids usually act by binding to surface molecules (proteins/sterols) inducing inhibition of the protein expression, and/or lysis of the cell (Bruneton, 1993). The expression of surface proteins of nematodes is stage specific (Rhoads and Fetterer, 1994).

Furthermore, Asteraceae is being the first and Fabaceae is the third largest terrestrial plant families all over the world and this could be another possible reason for such an extensive utilization of these families (Tariq et al., 2017). Plant species of Fabaceae are able to fix nitrogen, which leads to protein deposition in leaves and seeds (Molares and Ladio, 2012). Species of Lamiaceae could be easily cultivated and propagated, moreover, they are mostly utilized due to their strong aroma and ability to survive in severe hot weather because of their essential oils (Raja, 2012). Euphorbiaceae extensive use for different medicinal purposes may be attributed to its global distribution and mode of adaptation in the worst dry conditions because of the succulent nature of its species and crassulacean acid metabolism (CAM) pathway ability. Plants of this family possess a wide array of secondary metabolites and tendency of mutation load due to their exposure to a wide range of environmental conditions (Mwine and Van Damme, 2011).

Herbs were frequently used form of life against *H. contortus* eggs, larvae, and adult worms as compared to trees and shrubs. The dominancy of herbs over other forms of life could be attributed to their easy availability and high efficacy against different ailments as compared to shrubs and trees (Ahmad et al., 2009). Herbs are widely used in folk medicines all over the globe and contain a large number of active compounds responsible for their high efficacy and, therefore, are preferred by the scientists and traditional healers (Tariq et al., 2017).

Leaves were reported as the widely used part during pharmacological validation of medicinal plants against *H. contortus*. Leaves contain a variety of chemical compounds and due to their easy harvesting and less harmful effect on plant life, make them as the first choice of herbalists (Bhat et al., 2013; Tariq et al., 2017).

Medicinal plants owing to their potential of having a significant source of bioactive compounds that may lead to the development of novel drugs (Azwanida, 2015; Ali et al., 2020). Scientists have analyzed and evaluated the effect of various kinds of solvents, for the purpose to extract these bioactive compounds from various plant parts (Altemimi et al., 2017). Extraction is the separation of medicinally active portions of a plant, using selective solvents through standard procedures (Azwanida, 2015). The purpose of extraction is to separate the soluble plant metabolites, leaving behind the insoluble cellular marc (residue) (Azwanida, 2015). Methanol was the most preferred solvent for plant extraction possibly owing to its polar nature that ensures the release of several bioactive compounds from plants. It has been scientifically proven that highly polar solvents should be used to extract different bioactive compounds with high accuracy (Altemimi et al., 2017). Fruitful results of active compound in plants mainly depend upon the solvent used for herbal formulation.

The results revealed that more studies were conducted to evaluate *in vitro* anthelmintic activities of medicinal plants as compared to *in vivo*. *In vitro* validation of medicinal plants provides the proof of reliability of these plants against *H. contortus.* In veterinary parasitology several *in vitro* techniques are broadly used for analysis of nematicidal activity of drugs/plant extracts prior to *in vivo* testing (Sangster and Gill, 1999). There are several positive aspects of *in vitro* assays prior to *in vivo* including less time consuming, less expensive, need for a smaller number of animals, and permitting the evaluation of the efficacy of different anthelmintic compounds throughout the life cycle of the parasite (Demeler et al., 2013). Based on the reliable results obtained from *in vitro* analysis further selection of extract/pure compound for *in vivo* evaluation can be carried-out (Zips et al., 2005). *In vivo* studies are mainly conducted to evaluate the mechanism of action of the desired extract/compound, the immune response of the host animal, toxicity levels, as well as the *in vivo* effectiveness. Although there are many advantages of *in vivo* studies, but there are also some shortcomings including more time consuming, expensive, and lower precision and reproducibility (Lacey et al., 1990). These limitations should be taken into account and highlight the significance of pharmaceutical/pharmacokinetic studies for the industrial development of new anthelmintic products against *H. contortus*. The research to find effective and natural anthelmintics has been highly inundated with *in vitro* studies, hence, it is suggested to evaluate the plant extracts/compounds *in vivo* in future.

### Pharmacological Action

**FIGURE 6:**
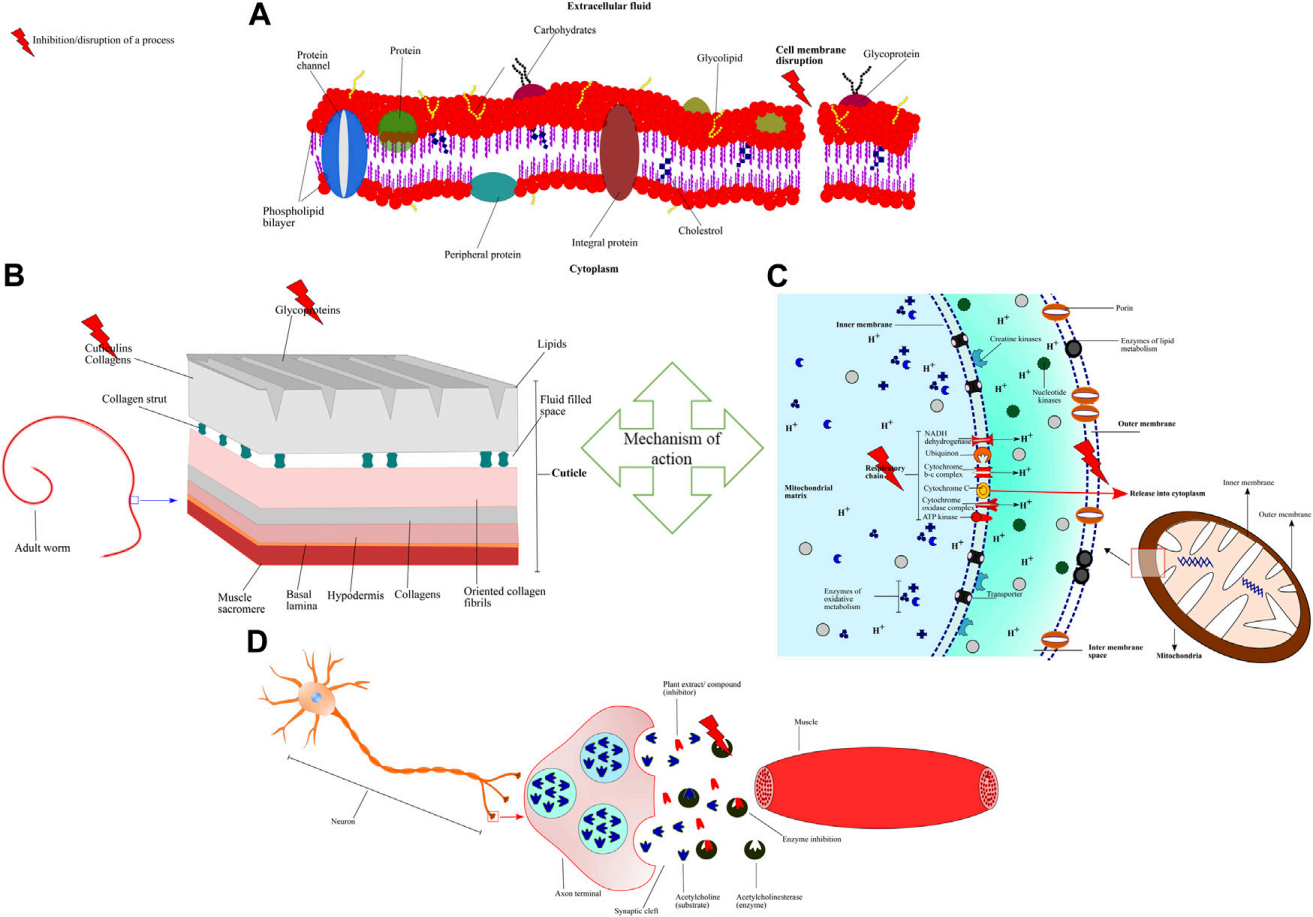
Schematic representation of mechanism of action and different pharmacological targets of plant extracts/compounds **(A)** Essential oils alter the permeability and cause depolarization of cytoplasmic membrane by interacting and disrupting the chemical structures of lipids, polysaccharides, and phospholipids **(B)** Condensed tannins (CT) bind to the cuticle proteins thus, inducing chemical and physical damage **(C)** Artemisinin disrupts the mitochondrial membrane potential and releases cytochrome c into the cytoplasm leading to inhibition of electron transfer and oxidative phosphorylation **(D)** Allium sativum inhibits Acetylcholinesterase (AChE) that hydrolyzes the neurotransmitter acetylcholine. Inhibition of ACheE leads to the accumulation of acetylcholine at the synaptic junction and disrupting the neuromuscular transmission which causes muscle paralysis.

Pharmacological activity of an extract/compounds/drug depends on how the candidate interacts with enzymes, proteins, nucleic acids, biomolecules, and different types of receptors (Roy, 2011). Pharmacological activity is an important phenomenon to know the precise target of the drug/extract/compound with anthelmintic efficacy against the parasite or other organism/pathogen under observation (Figure 6).

*A. sativum* ethanolic extract inhibits the motility of the *H. contortus* through a destructive and inhibitive effect on the enzyme acetylcholinesterase (AChE). The enzyme rapidly hydrolyzes acetylcholine (neurotransmitter) and thus, limits and terminates the cholinergic synaptic transmission (Taylor, 1990; Lee, 1996). Inhibition of ACheE leads to the accumulation of acetylcholine, thereby interrupting the neuromuscular transmission causes paralysis of musculature. Due to muscular discoordination/paralysis food swallowing and movement through the digestive system is stopped. The parasites enter the state of starvation and energy deprivation and thus, unable to survive inside the host (Kaur and Sood, 1982; Opperman and Chang, 1992). The antiparasitic activity of *A. sativum* may also be attributed to the sulfur containing compounds (e.g., ajoene and allicin) which can possibly form disulphide bonds with free thiol groups, and thus, inhibit enzymes or other proteins, which are important for survival of the parasite (Krstin et al., 2018). Crude aqueous extract of *Achillea millefolium* L. has profound anthelmintic activity, this could be due to the presence of several key chemical constituents one of which is eugenol. It is reported that eugenol can cause alteration of cell shape, membrane blebs (Machado et al., 2011), restrict cell growth, swelling, and collapsing cell membrane and arrest cell division (Ueda-Nakamura et al., 2006). The compound artemisinin of *A. absinthium* can inhibit vital enzymes of metabolic cascade by forming covalent bonds and resulting in irreversible inhibition of the enzyme (s) activities. The enzymes include S-adenosyl-methionine synthesase (SAMS), spermidine synthase (SpdSyn), L-lactate dehydrogenase (LDH), pyruvate kinase, and ornithine aminotransferase (OAT) (Wang et al., 2015). Artemisinin is also reported to disrupt mitochondrial membrane potential, cause cytochrome c release into the cytoplasm (Jia et al., 2016), and inhibit the electron transfer and oxidative phosphorylation of mitochondria, with final activation of caspase-3-mediated apoptosis (Li et al., 2005). Additionally, crude aqueous extract of *A. absinthium* can induce ultrastructural changes such as tegumental damage, nephridial canal epithelium lining and intrauterine eggs destruction, lipid accumulation, glycogen depletion, and finally worm paralysis and death (Beshay, 2018). Similar anthelmintic effects of tegumental disorganization/perforation and adult worm paralysis were also observed for *C. procera* aqueous and ethanolic extracts (Khalil et al., 2016). Bromelain extracted from *Ananas comosus* (L.) Merr. stem has been found as potent anthelmintic against gastrointestinal nematodes (Stepek et al., 2004; Domingues et al., 2013). Bromelain removes and digests the cuticle layer of nematodes resulting in immobility and death of the parasites (Stepek et al., 2004; Stepek et al., 2006).

*Azadirachta indica* A. Juss. leaves contain condensed tannins (CT) (Sakti et al., 2018), which facilitate diffusion of flavonoids by binding to the cuticle proteins (Kerboeuf et al., 2008). Flavonoids and CT inhibit secretion of key enzymes (e.g., esterase, tyrosin kinase, and nonspecific cholinesterase) that may cause fatal intracellular instability, neuromuscular disorganization, energy depletion, paralysis and death of parasites (Hoste et al., 2006; Kerboeuf et al., 2008).

Essential oils of *Corriandrum sativum* L., *C. citratus*, *C. schoenanthus*, *E. staigeriana* and *L. origanoides* were found highly effective against *H. contortus in vitro*. Essential oils may acquire this efficacy owing to a mixture of different chemical constituents whose interaction can result in compounds that inhibit or disorganize vital functions from the initial stages of development onward, interrupt with parasite metabolic activities, and interfere with drive mechanisms due to possible destructuring of the nervous system (Oka et al., 2000). Furthermore, essential oils can alter the permeability and cause depolarization of cytoplasmic membrane by interacting and disrupting the chemical structures of lipids, polysaccharides, and phospholipids (Bakkali et al., 2008). Phytochemical profile of *Cocos nucifera* L. revealed the presence of alkaloids, flavonoids, phenols, triterpenes, and condensed tannins among others (Lima et al., 2015). The compounds like flavonoids have antioxidant activities, while condensed tannins have shown antiparasitic activities by binding to proteins present in the cuticle, oral cavity, esophagus, and cloaca, thus inducing chemical and physical damage in the parasite (Costa et al., 2010). The efficacy of plant compounds may be attributed to the fact that they inhibit or retard the growth, maturation damage, suppress appetite or reduce procreative ability, which are all the causes of mortality. Moreover, the considerable activity of plants extracts may be due to the additive or synergistic relationship among different major components which can interact with multiple molecular targets in various developmental stages of the parasite to produce a pharmacological effect (Marie-Magdeleine et al., 2009).

*H. contortus* exposed to *C. citriodora* essential oils demonstrated ultrastructural changes, such as formations of vacuoles, disorganization of muscular layer, and changes in the mitochondrial profile. These changes suggest the loss of homeostasis and loss of motility due to muscular disorganization of the parasite (Araújo-Filho et al., 2019). *E. helioscopia* has monoterpens, which are lipophilic and can penetrate through cell membrane, induce expansion, increase permeability and disturb membrane structure and membrane embedded enzymes (Cox et al., 2000; Samy and Ignacimuthu, 2000; Cox et al., 2001). The nematicidal activity may also be attributed to the presence of tannins, which on the surface of nematodes can form complexes with proteins and result in alteration of metabolic pathways (Min et al., 2004; Ahmed, 2010) and enzymes (cysteine proteinases), which can damage the cuticle and kill the nematode parasites (Stepek et al., 2004; Stepek et al., 2006). Similarly, saponins, amino acids, and sterols can disturb proteins structure (Mabusela et al., 1990) therefore, affecting growth and reparation of nematode body. While compound muzigadial and ajoene has anti-feedant activity and inhibit proliferation of arterial smooth muscle cells and protein prenylation (Ferri et al., 2003; Mohanlall and Odhav, 2009). *L. latisiliquum* leaves forage utilization presented ultrastructural changes; for instance, disturbance of intestinal muscular cells/tissues and cytoplasmic vacuolization, suggesting that different secondary metabolites of leaves may provoke these changes. The alterations to the intestinal cells may be due to the ingestion of active compounds by the parasite, and the resulting direct contact between the bioactive compounds and the intestinal cells. The cytoplasmic vacuolization described can be interpreted as signs of disturbances in cellular functions, possibly due to imbalance of fluid exchanges between the intestinal and pseudocoelomic space and/or between the muscle and the pseudocoelomic space (Martínez-Ortiz-de-Montellano et al., 2019).

The high efficacy of *Nicotiana tabacum* L. against *H. contortus* could be attributed to the presence of nicotine, a ganglion stimulant (Bowman and Rand, 1980). Nematode muscles are known to contain excitatory neuromuscular junctions containing ganglion type nicotinic receptors with acetylcholine as their neurotransmitter (Neal, 2020). Any ganglion stimulant would tend to activate theses neuromuscular junctions causing a spastic paralysis in the worms leading to their death and expulsion from the host (Nouri et al., 2016). Among the reported plants with promising anti-haemonchiasis activity, *L. viridiflorum* leaves are rich in flavonoids and its anthelmintic action of egg hatching inhibition could be due to the effect on enzymatic activity and metabolic processes in helminthes (Kerboeuf et al., 2008).

Different protein contents e.g., proteases, ribosomal proteins, chitinases etc. isolated from *Spigelia anthelmia* L. were effective against different life stages of *H. contortus* (Araujo et al., 2017). Proteases can cause severe damage to the cuticle by disrupting cuticular proteins of the parasite (Liang et al., 2010), in larvae the proteases may hydrolyze/digest the proteins necessary for larval migration (Araujo et al., 2017), and also degrade the egg membrane during egg hatching (Mansfield et al., 1992). Chitinases were also identified which can degrade chitin present in the egg shell (Rogers and Brooks, 1977) and larva, inhibiting their development and leading to death (Rocha et al., 2015).

Comparative analysis also revealed that plant extracts/essential oils were more effective *in vitro* than *in vivo* against various stages of the parasite. Similar differences between *in vitro* and *in vivo* results with plant treatments have been previously reported (Peneluc et al., 2009; Nogueira et al., 2012) and might be related with bioavailability of plant chemical constituents in different parts of the ruminant gastrointestinal tract (Athanasiadou et al., 2007; Eguale et al., 2007b). Furthermore, adult nematodes may also be more resistant to the active components, or rumen microbiota may reduce the activity of metabolites (Nogueira et al., 2012), and other aspects, such as ruminal pH. Mostly, in gerbils the efficacy of plant extracts was reported to be comparatively low than the activity observed in sheep. Using rodents for evaluation of plants anthelmintic activity has some drawbacks, firstly the habitat for nematodes is quite different in rodents and small ruminants, hence, association between habitat and drug site absorption define the higher or lower drug activity (Hennessy, 1997). Secondly, different efficacy obtained in rodents and sheep can be described by mechanism of distribution and biotransformation of the drug in a monogastric and polygastric animal species. However, efficacy test on rodent nematodes can help researchers deduce the prescriptions to be used on sheep and goats (Camurça-Vasconcelos et al., 2007). The studies concerning isolation and purification of plant compounds responsible for antiparasitic acitivities are few and insufficient. Most of the studies reported the presence of key components of the plants which do not provide any information about the effective antiparasitic compounds and their mechanism of action. Therefore, pure comounds isolated from plants should be given focus for *in vitro*, *in vivo* evaluation, toxicology, and pharmacology studies in future research, this will provide base-line information for developing new ecofriendly and cost effective drugs with lesser side effects.

### Toxicity Evaluation

Safety issues of herbal medicines have been remained a big question and scientists are being interested in herbal medicines for decades. The notion that “natural” equals “safe” is apparently deceptive, since natural products comprise pharmacologically active compounds which, when taken in high doses or in specific conditions, can be detrimental to health. Bromelain of *A. comosus* is non-toxic and considered safe without any adverse effects and has shown good absorption and therapeutic benefits (Maurer, 2001). Moreover, no alteration in body weight, food, and water consumption was observed. The enzymes, urea, and creatinine levels of serum were also unaltered and no significant difference was observed (Dutta and Bhattacharyya, 2013). *A. millefolium* aqueous extract oral and intraperitoneal administration produced no significant biochemical and histopathological changes in Wistar rats (Cavalcanti et al., 2006). No relevant signs of toxicity were observed for longer periods of exposure, however, slight changes in blood glucose and cholesterol levels, and liver weight were detected, neither correlated with dose or period of exposure nor suggestive of toxicity (Cavalcanti et al., 2006). *C. sativum* was safe and no effects on hematological profile, histology, relative organ weights, and plasma markers of damage vital organs were found. However, a significant body weight loss was observed due to reduction in food intake (Patel et al., 2012), which is suggestive of the disturbances in carbohydrates, proteins, and fats (Ecobichon and Klaassen, 2001). *C. citratus* was safe and produced no toxic effects when tested against mice peritoneal macrophages (Santoro et al., 2007). Similarly, *C. schoenanthus* depicted no toxicological effects on hepatic and renal parameters in lambs (Katiki et al., 2012).

The ethyl acetate extract of *C. nucifera* presented no acute oral toxicity at the tested doses. However, the intraperitoneal and intramuscular administration was toxic (Tayler et al., 2020). Moreover, the hemoglobin level fallen below the normal limit after 8 days, suggesting that the extract could have negative effects if used for longer periods of time (Tayler et al., 2020). Similarly, *E. staigeriana* essential oil when administered orally was non-toxic, while intraperitoneal administration did not depict similar results (Macedo et al., 2010). Traditionally, when a substance administered orally and show LD_50_ value equal to 1000 mg/kg is considered to be safe or less toxic (Garner et al., 1961). The observed difference in toxicity may be attributed to the fact that after oral administration, the extract may be poorly absorbed, detoxified by the liver (Hayes and Loomis, 1996) or degraded by the stomach and gut digestive enzymes, however, during intraperitoneal administration the absorption is systematic and toxicity is stronger and appear earlier (Hayes and Loomis, 1996; Obici et al., 2008).

*E. helioscopia* was safe and produced no physiological alternations of vital organs and the biochemical parameters were also unchanged at the tested doses (Saleem et al., 2016). *S. anthelmia* and *N. tabacum* did not affect the body weight and animal behavior and were considered to be non-toxic (Ribeiro et al., 2017; Andjani et al., 2019).

Low toxicity of *A. sativum* and *L. viridiflorum* was observed on human HaCat and mammalian macrophages cells, respectively, (Krstin et al., 2018; de Melo et al., 2020). Mice became dead after oral administration of *C. citriodora* essential oil and citronellal suggesting toxicity of the plant species (Araújo-Filho et al., 2019). Prolonged use of *A. absinthium* and *A. herba-alba* lead to neurotoxicity and infertility by affecting the reproductive system, respectively (Almasad et al., 2007; Lachenmeier, 2010). *A. indica* poisoning affect was dose and time dependent and histopathological analysis showed that the testicles, liver, and kidneys were the organs affected (Deng et al., 2013). *C. procera* latex was found to be toxic at the tested doses, animals developed signs of nervousness, salivation, urination, dyspnea, tachycardia, and loss of condition. Severe pathological changes in intestines, heart, liver, kidneys, lungs, and brain were also observed (Mahmoud et al., 1979). A slight change (decrease) in the serum biochemical profile was also observed. This decrease in serum zinc, iron, and copper concentration (Al-Qarawi et al., 2001) might be due to continue (i) interference of adult parasites in the abomasum with digestibility and absorption of nutritive substances as a results of existing damage to the abomasal mucosa and its digestive function, or (ii) effects of unknown toxic principles elaborated by the worm.

## Conclusion and Future Recommendations

Mostly, *in vitro* studies have been performed to evaluate the anti-haemonchiasis activity of plants. *In vitro* studies have a key role in initial screening however, these studies provide no information of bioavailability, toxicity, and *in vivo* efficacy of tested extract/compound. Hence, in future *in vivo* studies by using suitable animal models should be carried out to understand the pharmacokinetics and pharmacodynamics of the tested extract/compound. Most of the *in vivo* studies provide no evidence about toxicity and mechanism of action of the medicinal plant/compound, this is the most neglected aspect and strongly suggested to researchers to evaluate toxicity levels and pharmacological action of the tested plant/compound. *Mentha x vilosa* Huds.*, Anthemis nobilis* L (syn. *Chamaemelum nobilis* (L.) All.)*, Lantana camara* L.*, Trichilia claussenii* C. DC*., Croton macrostachyus* Hochst ex Delile*, Lavandula officinalis* (Chaix and Kitt.), *Coleus maculosus* subsp. edulis (Vatke) A.J.Paton (*Plectranthus punctatus* (L.f) L’Her.), *Maesa lanceolata* Forssk.*,* and *Foeniculum vulgare* Mill. among others were highly effective *in vitro* against different life stages of the parasite, however, these plant species are not tested for *in vivo* efficacy. These plants should be evaluated for *in vivo* anti-haemonchiasis activity along with phytochemical profile, toxicological effects, and pharmacological activity. Most of the reported studies provide no information regarding time exposure and LC_50_ values of medicinal plant/compound used for *in vitro* evaluation. Time exposure and LC_50_ are very important parameters to understand the accurate efficacy of medicinal plants, therefore, it is recommended to provide this information in future studies. Plants contain a number of different compounds, which act synergistically to perform an activity, only few compounds have been isolated and tested *in vitro*/*in vivo* against *H. contortus*. It is recommended to identify/isolate individual compounds and evaluate their activity*,* this will provide more precise and in depth information of anti-haemonchiasis potential of the medicinal plant under observation. Plant compounds cinnamaldehyde, thymol, and carvacrol have revealed high efficacy in *in vitro* studies, it is recommended to further investigate these compounds for *in vivo* activity. Carvone and anethole have shown promising anti-haemonchiasis potential *in vitro* and *in vivo*, however, their toxicity levels and pharmacological effects are unknown and should be investigated in future studies. *L. viridiflorum* has revealed high efficacy both *in vitro* and *in vivo* and has no adverse/toxic effects after oral administration and considered to be safe, it is recommended to pharmaceutical industries to further investigate this plant species because it could be an alternative candidate for drug development against *H. contortus*.

## Data Availability

The original contributions presented in the study are included in the article/Supplementary Material, further inquiries can be directed to the corresponding authors.
