# Supplementary material for: A Systematic Review on Comparative Analysis, Toxicology, and Pharmacology of Medicinal Plants Against Haemonchus contortus
Source: Front Pharmacol. 2021 May 10;12:644027. doi: 10.3389/fphar.2021.644027 (PMC8141741; doi:10.3389/fphar.2021.644027)
Supplement: Supplementary file 1 [file Table1.DOCX]

Supplementary Table 1: PRISMA checklist

| **Section/topic** | **#** | | **Checklist item** | | **Reported on page #** | |
| --- | --- | --- | --- | --- | --- | --- |
| **TITLE** | | | | |  | |
| Title | 1 | | Identify the report as a systematic review, meta-analysis, or both. | | Title page | |
| **ABSTRACT** | | | | |  | |
| Structured summary | 2 | | Provide a structured summary including, as applicable: background; objectives; data sources; study eligibility criteria, participants, and interventions; study appraisal and synthesis methods; results; limitations; conclusions and implications of key findings; systematic review registration number. | | Abstract | |
| **INTRODUCTION** | | | | |  | |
| Rationale | 3 | | Describe the rationale for the review in the context of what is already known. | | Introduction, paragraph 2, 3 | |
| Objectives | 4 | | Provide an explicit statement of questions being addressed with reference to participants, interventions, comparisons, outcomes, and study design (PICOS). | | Introduction , paragraph 4 | |
| **METHODS** | | | | |  | |
| Protocol and registration | 5 | | Indicate if a review protocol exists, if and where it can be accessed (e.g., Web address), and, if available, provide registration information including registration number. | | “There is no protocol for this review this systematic review.” Methods, paragraph 1 | |
| Eligibility criteria | 6 | | Specify study characteristics (e.g., PICOS, length of follow-up) and report characteristics (e.g., years considered, language, publication status) used as criteria for eligibility, giving rationale. | | Methods, paragraph 3 | |
| Information sources | 7 | | Describe all information sources (e.g., databases with dates of coverage, contact with study authors to identify additional studies) in the search and date last searched. | | Methods, paragraph 2 | |
| Search | 8 | | Present full electronic search strategy for at least one database, including any limits used, such that it could be repeated. | | Methods, paragraph 2 | |
| Study selection | 9 | | State the process for selecting studies (i.e., screening, eligibility, included in systematic review, and, if applicable, included in the meta-analysis). | | Methods, paragraph 4 | |
| Data collection process | 10 | | Describe method of data extraction from reports (e.g., piloted forms, independently, in duplicate) and any processes for obtaining and confirming data from investigators. | | Methods, paragraph 4 | |
| Data items | 11 | | List and define all variables for which data were sought (e.g., PICOS, funding sources) and any assumptions and simplifications made. | | Methods, paragraph 4 | |
| Risk of bias in individual studies | 12 | | Describe methods used for assessing risk of bias of individual studies (including specification of whether this was done at the study or outcome level), and how this information is to be used in any data synthesis. | | N/A | |
| Summary measures | 13 | | State the principal summary measures (e.g., risk ratio, difference in means). | | N/A | |
| Synthesis of results | 14 | | Describe the methods of handling data and combining results of studies, if done, including measures of consistency (e.g., I^2^) for each meta-analysis. | | N/A | |
| Risk of bias across studies | 15 | | Specify any assessment of risk of bias that may affect the cumulative evidence (e.g., publication bias, selective reporting within studies). | | N/A | |
| Additional analyses | 16 | | Describe methods of additional analyses (e.g., sensitivity or subgroup analyses, meta-regression), if done, indicating which were pre-specified. | | N/A | |
| **RESULTS** | | | | | |  |
| Study selection | | 17 | | Give numbers of studies screened, assessed for eligibility, and included in the review, with reasons for exclusions at each stage, ideally with a flow diagram. | | Results, paragraph 1 |
| Study characteristics | | 18 | | For each study, present characteristics for which data were extracted (e.g., study size, PICOS, follow-up period) and provide the citations. | | Results, paragraph 2-12 |
| Risk of bias within studies | | 19 | | Present data on risk of bias of each study and, if available, any outcome level assessment (see item 12). | | N/A |
| Results of individual studies | | 20 | | For all outcomes considered (benefits or harms), present, for each study: (a) simple summary data for each intervention group (b) effect estimates and confidence intervals, ideally with a forest plot. | | N/A |
| Synthesis of results | | 21 | | Present results of each meta-analysis done, including confidence intervals and measures of consistency. | | N/A |
| Risk of bias across studies | | 22 | | Present results of any assessment of risk of bias across studies (see Item 15). | | N/A |
| Additional analysis | | 23 | | Give results of additional analyses, if done (e.g., sensitivity or subgroup analyses, meta-regression [see Item 16]). | | N/A |
| **DISCUSSION** | | | | | |  |
| Summary of evidence | | 24 | | Summarize the main findings including the strength of evidence for each main outcome; consider their relevance to key groups (e.g., healthcare providers, users, and policy makers). | | Discussion, paragraph 1-18 |
| Limitations | | 25 | | Discuss limitations at study and outcome level (e.g., risk of bias), and at review-level (e.g., incomplete retrieval of identified research, reporting bias). | | N/A |
| Conclusions | | 26 | | Provide a general interpretation of the results in the context of other evidence, and implications for future research. | | Discussion, paragraph 19 |
| **FUNDING** | | | | | |  |
| Funding | | 27 | | Describe sources of funding for the systematic review and other support (e.g., supply of data); role of funders for the systematic review. | | N/A |

Supplementary Table 2: *In-vitro* efficacy of medicinal plants against *H. contortus*

| **Family** | **Plant name/ Local name/ Habit** | **Country** | **Part used** | **Extract** | **Type of assay** | **Concentration (mg/ml)** | **Time exposure (min)** | **Inhibition (%)** | **Other uses** | **References** |
| --- | --- | --- | --- | --- | --- | --- | --- | --- | --- | --- |
| Amaranthaceae | *Alternanthera sessilis*(L.) R.Br. ex DC. / Sachi-shak/ Herb | Bangladesh | Whole plant | Ethanol | AWMT | 50  25  12.5  6.25  3.13  1.5f0 | 120 | 8.33  6.67  5.00  4.00  1.67  5.33 | Helminthiasis | (Mondal et al., 2015) |
|  |  |  |  |  |  | 50  25  12.5  6.25  3.13  1.50 | 240 | 10.00  9.00  7.33  5.33  4.00  1.67 |  |  |
|  |  |  |  |  |  | 50  25  12.5  6.25  3.13  1.50 | 360 | 10.00  10.00  9.33  7.33  5.67  3.3 |  |  |
|  |  |  |  |  |  | 50  25  12.5  6.25  3.13  1.50 | 480 | 10.00  10.00  10.00  9.33  7.67  5.33 |  |  |
|  | *Chenopodium album* L./Bathu/ Herb | Pakistan | Whole plant | Aqueous | EHT | 48 | 360 |  | Anthelmintic activity | (Jabbar et al., 2007) |
|  |  |  |  | Methanol | AME |  |  |  |  |  |
|  | *Chenopodium ambrosioides* L.*/* NA/ Herb | Mexico | Aerial parts | n-hexane | LDT | 1.25  2.5  5  10  20  40 | 1440 | 8.2  7.6  58.2  77.5  92.8  91.1 |  | (Zamilpa et al., 2019) |
|  |  |  |  |  |  | 1.25  2.5  5  10  20  40 | 4320 | 47.9  67.0  83.9  93.4  89.3  96.3 |  |  |
| Amaryllidaceae | *Allium sativum* L./ Garlic, Lahsan/ Herb | India | Bulb | Ethanol | AWMT | 0.5 | 420 | 88.5 | Antibacterial, antiviral, antifungal, and Antiparasitic | (Veerakumari and Chitra, 2016) |
|  |  | Pakistan | Bulb | Methanol | AWMT | NA | 120 | 100 | Anthelmintic activity | (Iqbal et al., 2001b) |
|  |  | South Africa | NA | Ethanol | LMT | 100  200  300 | NA | 68.0  77.0  84.0 | NA | (Ahmed et al., 2013) |
|  | *Scadoxus puniceus*(L.) Friis & Nordal/ Chinese bush clover/ Herb | South Africa | NA | Ethanol | LMT | 100  200  300 | NA | 15.6  50.0  59.4 | NA | (Ahmed et al., 2013) |
| Acanthaceae | *Andrographis* paniculata (Burm.f.) Nees/ NA/ Herb | India | Leaves | Ethyl acetate | EHT | 25  12.5  6.25 |  | 88.6  75.8  64.2 | Larvicidal effect | (Kamaraj et al., 2011) |
|  |  |  |  | Acetone |  | 25  12.5  6.25 |  | 87.0  74.0  57.8 |  |  |
|  |  |  |  | Methanol |  | 25  12.5  6.25 |  | 100  84.2  74.0 |  |  |
|  |  |  |  | Ethyl acetate | LDT | 25  12.5  6.25 |  | 94.4  73.8  62.8 |  |  |
|  |  |  |  | Acetone |  | 25  12.5  6.25 |  | 78.8  62.8  49.8 |  |  |
|  |  |  |  | Methanol |  | 25  12.5  6.25 |  | 100  88.6  64.4 |  |  |
|  | *Adhatoda vasica* Nees.(=*Justicia* *adhatoda* L.) /NA/ Shrub | Pakistan | NA | Aqueous | EHT | 3.12  6.20  12.50  25.00  50.00 |  | 13.3  21.7  40.7  74.3  81.0 | Anthelmintic | (Al-Shaibani et al., 2008) |
|  |  |  |  | Ethanolic |  | 3.12  6.20  12.50  25.00  50.00 |  | 13.0  22.3  46.3  80.0  88.0 |  |  |
|  |  |  |  | Aqueous | LDT | 3.12  6.20  12.50  25.00  50.00 |  | 11.3  15.3  47.7  74.7  80.7 |  |  |
|  |  |  |  | Ethanolic |  | 3.12  6.20  12.50  25.00  50.00 |  | 14.7  26.7  47.7  74.0  82.7 |  |  |
|  | *Avicennia* *germinans* (L.) L. */* NA/ Shrub | Mexico | Leaves | Acetone water | EHT | 3.6 |  | 5.58 |  | (Vargas-Magaña et al., 2014) |
|  |  |  |  |  | LMT | 3.6 |  | 47.98 |  |  |
| Anacardiaceae | *Anacardium humile* A. St.-Hil.*/* Cajuzinho-do-cerrado/ Shrub | Brazil | Leaves | Aqueous | LDT | 30.0  50.0  100.0  150.0  187.5 |  | 62.3  93.2  90.9  97.3  100 |  | (Nery et al., 2010) |
|  |  |  |  | Ethanolic | LDT | 20  40  60  80  100 |  | 47.7  83.8  99.6  99.6  99.6 |  |  |
|  | *Rhus aromatica*Aiton/ NA/ Shrub | United state | Leaves | Methanol with 0.5% DMSO | EHT | 50 |  | 100 |  | (Acharya et al., 2014) |
|  |  |  |  | Methanol with 0.5% MOPS |  | 50 |  | 11.8 |  |  |
|  | *Rhus glabra* L.*/* Kimo/ Shrub | Ethiopia | Leaves | Aqueous  Hydro-alcoholic | EHT | 2  2 | 2880 | 66  51 | Anthelmintic | (Getachew et al., 2012) |
|  |  |  |  | Aqueous  Hydro-alcoholic | LDT | 50  50 |  | 86  97 |  |  |
|  | *Sclerocarya* *birrea* (A.Rich.) Hochst. / NA/ Tree | South Africa | Fruit | Acetone | EHT | 2.5 | NA | 28 |  | (Fouche et al., 2016) |
|  | [*Searsia* *pyroides* (Burch.) Moffett](http://www.theplantlist.org/tpl1.1/record/kew-2608664) (=*Rhus vulgaris* Meikle)*/*NA/ Tree | Ethiopia | Leaves | Aqueous  Hydro-alcoholic | EHT | 2  2 | 2880 | 90  67 | Anthelmintic | (Getachew et al., 2012) |
|  |  |  |  | Aqueous  Hydro-alcoholic | LDT | 50  50 |  | 10  63 |  |  |
|  | *Myracrodruon urundeuva* Allemao/ Aroeira-do-sertao/ Tree | Brazil | Leaves | Acetone | EHT | 0.3125  0.625  1.25  2.5  5 |  | 24.93  69.23  97.73  100  100 | anthelmintic | (de Oliveira et al., 2011) |
|  |  |  | Stem | Acetone | EHT | 0.3125  0.625  1.25  2.5  5 |  | 16.82  20.07  30.66  61.03  83.56 |  |  |
|  |  | Brazil | Seeds | Ethanol | LDA | 0.29 |  | 50 | Antimicrobial, antiparasitic | (Soares et al., 2018) |
| Annonaceae | *Annona muricata* L./ Graviola/Tree | Brazil | Leaves | Aqueous | EHT | 62.5  125  250  500 |  | 47.42  66.97  79.12  84.91 | Anthelmintic, antipyretic, sedative, antispasmodic, anticonvulsant, hypotensive | (Ferreira et al., 2013) |
|  |  |  |  |  | LMT | 62.5  125  250  500 |  | 30.47  74.62  89.08  83.29 |  |  |
|  | *Annona senegalensis* Pers./NA/ Shrub | Cameroon | Leaves | Crude ethanolic | EHT | 8 | 10080 | 95 |  | (Monglo et al., 2006) |
|  |  |  |  |  | LMT | 8 | 2880 | 98.33 |  |  |
|  | *Annona* squamosa L./ NA/ Shrub | India | Leaves | Ethyle acetate | EHT | 50  25  12.5  6.25  3.125 |  | 87.4  74.4  53.0  36.6  16.8 | Ovicidal and larvicidal activity | (Kamaraj and Rahuman, 2011) |
|  |  |  |  | Acetone |  | 50  25  12.5  6.25  3.125 |  | 88.0  69.6  52.4  32.2  13.2 |  |  |
|  |  |  |  | Methanol |  | 50  25  12.5  6.25  3.125 |  | 100  88.6  75.6  53.6  15.8 |  |  |
|  |  |  |  | Ethyl acetate |  | 50  25  12.5  6.25  3.125 |  | 87.4  74.4  53.0  36.6  16.8 |  |  |
|  |  | India | Bark | Ethyl acetate | EHT | 25  12.5  6.25 |  | 94.0  80.8  68.8 |  | (Kamaraj et al., 2011) |
|  |  |  |  | Acetone |  | 25  12.5  6.25 |  | 91.0  78.2  69.4 |  |  |
|  |  |  |  | Methanol |  | 25  12.5  6.25 |  | 100  92.6  68.6 |  |  |
|  |  |  |  | Ethyl acetate | LDT | 25  12.5  6.25 |  | 94.0  76.0  51.0 |  |  |
|  |  |  |  | Acetone |  | 25  12.5  6.25 |  | 90.8  70.2  47.4 |  |  |
|  |  |  |  | Methanol |  | 25  12.5  6.25 |  | 100  93.0  77.4 |  |  |
|  |  | India | Leaves | Ethyl acetate |  | 50  25  12.5 |  | 87.4  74.4  53.0 | Pesticidal and parasiticidal, acaricidal and insecticidal | (Kamaraj et al., 2011) |
|  |  |  |  | Acetone |  | 50  25  12.5 |  | 88.0  69.6  52.4 |  |  |
|  |  |  |  | Methanol |  | 50  25  12.5 |  | 100  88.6  75.6 |  |  |
| Apiaceae | *Coriandrum sativum* L.*/* Dimbilal/ Herb | Ethiopia | Seeds | Essential oil | EHT | 0.624  1.25  2.5  5  10 |  | 26.37  41.27  54.33  68.60  83.63 | Stomachache, hepatitis | (Hussien et al., 2011) |
|  | *Foeniculum vulgare* Mill./ Fennel/Herb | Slovak Republic | Seeds | Methanol  Aqueous | EHT | 1.024 1.563 |  | 18.9  8.7 | Anthelmintic | (Váradyová et al., 2018) |
|  |  | Ethiopia | Leaves | Aqueous  Hydro-alcoholic | EHT | 1  1 | 2880 | 99.4  100 | Gonorrhea | (Getachew et al., 2012) |
|  |  |  |  | Aqueous  Hydro-alcoholic | LDT | 50  50 |  | 31  90 |  |  |
|  | *Perideridia gairdneri* (Hook. & Arn.)/ Common yampah/ Herb | United states | Roots | Methanol with 0.5% DMSO | EHT | 50 |  | 100 |  | (Acharya et al., 2014) |
|  |  |  |  | Methanol with 0.5% MOPS | EHT | 50 |  | 100 |  |  |
| Apocynaceae | *Acokanthera schimperi* (A.DC.) Schweinf. */* Mirenz/ Shrub | Ethiopia | Leaves | Aqueous  Hydro-alcoholic | EHT | 2  2 | 2880 | 53  86 | Treatment of snake bite and tape worm infection | (Getachew et al., 2012) |
|  |  |  |  | Aqueous  Hydro-alcoholic | LDT | 50  50 |  | 83  90 |  |  |
|  | *Calotropis procera* (Aiton) Dryand. */* NA/ Shrub | Pakistan | Flowers | Crude aqueous | AWMT | 25 | 360 | 70 | Anthelmintic activity | (Iqbal et al., 2005) |
|  |  |  |  | Methanol |  |  |  | 57 |  |  |
|  |  | Brazil | Latex | Ethyl acetate | EHT | 1  2  4 |  | 29.3  48.2  91.0 | Anthelmintic | (Cavalcante et al., 2016) |
|  |  |  |  | Ethyl acetate | LDT | 1  2  4 |  | 99.8  48.2  91.0 |  |  |
|  | *Catharanthus roseus* (L.) G. Don/ NA/ Herb | India | Leaves | Ethyle acetate | EHT | 50  25  12.5  6.25  3.125 |  | 100  84.4  75.8  59.8  29.4 | Ovicidal and larvicidal activity | (Kamaraj and Rahuman, 2011) |
|  |  |  |  | Acetone |  | 50  25  12.5  6.25  3.125 |  | 92.2  81.0  75.4  52.0  27.2 |  |  |
|  |  |  |  | Methanol |  | 50  25  12.5  6.25  3.125 |  | 94.0  86.0  69.0  38.6  19.4 |  |  |
|  |  | India | Leaves | Ethyl acetate | LDT | 50  25  12.5 |  | 100  89.4  75.8 |  | (Kamaraj and Rahuman, 2011) |
|  |  |  |  | Acetone |  | 50  25  12.5 |  | 92.4  81.0  75.4 |  |  |
|  |  |  |  | Methanol |  | 50  25  12.5 |  | 94.0  86.0  69.0 |  |  |
|  | *Tabernaemontana citrifolia* L./ NA/ Shrub | France | Leaves | Aqueous | EHT | - |  | 2 |  | (Marie-Magdeleine et al., 2010) |
|  |  |  |  |  | LDT | - |  | 75 |  |  |
|  |  |  |  |  | LMT | - |  | 20.6 |  |  |
|  |  |  |  |  | AWMT | - |  | 25.1 |  |  |
|  |  |  |  | Methanol | EHT | - |  | 1.6 |  |  |
|  |  |  |  |  | LDT | - |  | 76 |  |  |
|  |  |  |  |  | LMT | - |  | 49.4 |  |  |
|  |  |  |  |  | AWMT | - |  | 49.89 |  |  |
|  |  |  |  | Dichloromethane | EHT | - |  | 6.5 |  |  |
|  |  |  |  |  | LDT | - |  | 33.5 |  |  |
|  |  |  |  |  | LMT | - |  | 8.2 |  |  |
|  |  |  |  |  | AWMT | - |  | 29.8 |  |  |
|  |  |  | Fruit | Aqueous | EHT | - |  | 6.4 |  |  |
|  |  |  |  |  | LDT | - |  | 89.9 |  |  |
|  |  |  |  |  | LMT | - |  | 11.5 |  |  |
|  |  |  |  |  | AWMT | - |  | 3.1 |  |  |
|  |  |  |  | Methanol | EHT | - |  | 8.2 |  |  |
|  |  |  |  |  | LDT | - |  | 92 |  |  |
|  |  |  |  |  | LMT | - |  | 20.6 |  |  |
|  |  |  |  |  | AWMT | - |  | 20.4 |  |  |
|  |  |  |  | Dichloromethane | EHT | - |  | 12.7 |  |  |
|  |  |  |  |  | LDT | - |  | 99.8 |  |  |
|  |  |  |  |  | LMT | - |  | 4.7 |  |  |
|  |  |  |  |  | AWMT | - |  | 47.3 |  |  |
|  |  |  | Roots | Aqueous | EHT | - |  | 7.5 |  |  |
|  |  |  |  |  | LDT | - |  | 72.1 |  |  |
|  |  |  |  |  | LMT | - |  | 9.3 |  |  |
|  |  |  |  |  | AWMT | - |  | 34.4 |  |  |
|  |  |  |  | Methanol | EHT | - |  | 16.6 |  |  |
|  |  |  |  |  | LDT | - |  | 83.6 |  |  |
|  |  |  |  |  | LMT | - |  | 18.7 |  |  |
|  |  |  |  |  | AWMT | - |  | 31.6 |  |  |
|  |  |  |  | Dichloromethane | EHT | - |  | 22.7 |  |  |
|  |  |  |  |  | LDT | - |  | 83.8 |  |  |
|  |  |  |  |  | LMT | - |  | 16.9 |  |  |
|  |  |  |  |  | AWMT | - |  | 5.3 |  |  |
|  | *Tabernaemontana elegans*Stapf/ NA/ Tree | South Africa | Leaves | Acetone | EHT | 5 | NA | 47 |  | (Fouche et al., 2016) |
| Araceae | *Arisaema* *franchetianum* Engl./ NA/ Herb | China | Leaves | Essential oil | EHT | 10 |  | 100 | Anti-parastitic, anti-malarial | (Zhu et al., 2013a) |
|  |  |  |  |  | LDA | 10 |  | 100 |  |  |
|  |  |  |  |  | LMIA | 10 |  | 74.1 |  |  |
|  | *Arisaema lobatum* Engl. */* NA/ Herb | China | Leaves | Essential oil |  | 10 |  | 100 | Anti-parastitic, anti-malarial | (Zhu et al., 2013a) |
|  |  |  |  |  |  | 10 |  | 100 |  |  |
|  |  |  |  |  |  | 10 |  | 95.6 |  |  |
| Asteraceae (=Compositeae)  ‎ | *Achillea millefolium* L./ Pehl-ghasa/ Herb | India | Whole plant | Crude aqueous | AWMT | 25 |  | 94.44 | Vermifuge | (Tariq et al., 2008) |
|  |  |  |  | Crude ethanolic | AWMT | 25 |  | 88.88 |  |  |
|  | *Anthemis* *nobilis* L. (= *Chamaemelum* *nobilis* (L.) All.)/ NA/ Herb | Brazil | Flowers | Essential oil | EHT | 1.562  3.125  6.25  12.5  25  50 |  | 86.9  95.9  96.4  98.9  100  100 | Anthelmintic | (Ferreira et al., 2018) |
|  |  |  |  |  | LDT | 0.0937  0.187  0.375  0.75  1.5  3.0 |  | 71.5  85.4  90.1  100  100  100 |  |  |
|  |  |  |  |  | AWMT | 0.05  5  50 | 720  600  600 | 100  100  100 |  |  |
|  | *Artemisia absinthium* L./ Wormwood/ Herb | Slovak Republic | Stem | Methanol  Aqueous | EHT | 1.024  1.563 |  | 100  16.8 | Anti-parasitic, anti-bacterial | (Váradyová et al., 2018) |
|  |  | India | Whole plant | Crude aqueous | AWMT | 25 | 480 | 65 | Anti-septic, anti-bacterial, anti-fungal | (Tariq et al., 2009) |
|  |  |  |  | Crude ethanolic | AWMT | 25 | 480 | 85 |  |  |
|  | *Artemisia herba-alba* Asso/ NA/ Shrub | Ethiopia | Flower | Methanol | EHT | 0.1  0.25  0.5  1 | 420 | 58  83  84  98 |  | (Ahmed et al., 2020) |
|  |  |  |  |  | AMT | 1.25  2.5  5  10 |  | 100  100  100  100 |  |  |
|  |  |  | Aerial parts |  | EHT | 0.1  0.25  0.5  1 |  | 58  60  68  82 |  |  |
|  |  |  |  |  | AMT | 1.25  2.5  5  10 |  | 100  100  100  100 |  |  |
|  | *Artemisia vulgaris* L./ Mugwort / Herb | Bangladesh | Aerial parts | Ethanolic | EHT | 5  10  20 |  | 100  100  100  100  100  100 |  | (Karim et al., 2019) |
|  |  |  |  |  | LMT | 5  10  20 | 1440 |  |  |  |
|  |  |  |  |  | AMT | 5  10  20 |  | 100  100  100 |  |  |
|  |  |  |  | Aqueous | EHT | 5  10  20 | 1440 | 75  100  100 |  |  |
|  |  |  |  |  | LMT | 5  10  20 |  | 67.33  78.67  100 |  |  |
|  |  |  |  |  | AMT | 5  10  20 |  | 93.33  100  100 |  |  |
|  |  | Brazil | Leaves | Essential oil | EHT | 10 |  | 7.4 | Dyspepsia, rheumatic, worm infestaion | (Malik et al., 2019) |
|  |  |  |  |  | LET | 10 |  | 4.1 |  |  |
|  |  |  |  |  | LMIT | 1.2 |  | 1.9 |  |  |
|  | *[Artemisia vestita](https://pfaf.org/user/Plant.aspx?LatinName=Artemisia+vestita)* [Wall. ex Besser](https://pfaf.org/user/Plant.aspx?LatinName=Artemisia+vestita)[/ NA/ Shrub](https://pfaf.org/user/Plant.aspx?LatinName=Artemisia+vestita) | Pakistan | Whole plant | Methanol | LDT | 50 |  | 95 | Anthelmintic activity | (Irum et al., 2015) |
|  |  |  |  |  | APMT | 25 |  | 100 |  |  |
|  | *Artemisia maritima* L.*/* NA/ Herb | Pakistan | Whole plant | Methanol | LDT | 25 |  | 100 |  |  |
|  |  |  |  |  | APMT | 25 |  | 100 |  |  |
|  | *Artemisia lancea* Vaniot/ NA/ Shrub | China | Whole plant | Essential oil | EHT | 0.63  1.25  2.5  5.0  10.0 |  | 16.4  38.4  58.4  78.0  99.4 | Anti-fungal, anti-viral, anti-bacterial | (Zhu et al., 2013b) |
|  |  |  |  |  | LDT | 0.63  1.25  2.5  5.0  10.0 |  | 25.8  42.6  58.0  76.2  93.6 |  |  |
|  |  |  |  |  | LMT | 0.63  1.25  2.5  5.0  10.0 |  | 25.8  45.0  56.5  69.1  79.6 |  |  |
|  | *Baccharis conferta* Kunth/ Azoyote/ Shrub | Mexico | Aerial parts | Methanol  Ethyl acetate | EHT | 50  2 |  | 34.3  100 | Digestive disorders, cramps, seizures | (Cortes-Morales et al., 2019) |
|  | [*Baccharoides* *anthelmintica* (L.) Moench](http://www.theplantlist.org/tpl1.1/record/gcc-111955) (=*Vernonia anthelmintica* (L.) Willd.*/*NA/ Herb | Pakistan | Seeds | Crude aqueous |  | 25 | 360 |  | Anthelmintic | (Iqbal et al., 2006a) |
|  |  |  |  | Methanol |  |  |  |  |  |  |
|  | *Chrysothamnus viscidiflorus*(Hook.) Nutt/ Yellow rabbit brush/ Herb | United States | Leaves | Methanol with 0.5% DMSO | EHT | 50 |  | 100 |  | (Acharya et al., 2014) |
|  |  |  |  | Methanol with 0.5% MOPS | EHT | 50 |  | 100 |  |  |
|  | *Eclipta prostrata* (L.) L./ NA/ Herb | India | Leaves | Ethyl acetate |  | 50  25  12.5 |  | 88.4  73.2  62.8 |  | (Kamaraj and Rahuman, 2011) |
|  |  |  |  | Acetone |  | 50  25  12.5 |  | 83.8  71.6  53.8 |  |  |
|  |  |  |  | Methanol |  | 50  25  12.5 |  | 100  90.4  65.6 |  |  |
|  | *Ericameria nauseosa*(Pall. ex Pursh) G.L.Nesom & G.I.Baird/ Chamisa, rubber rabbitbrush/ Shrub | United state | Leaves | Methanol with 0.5% DMSO | EHT | 50 |  | 100 |  | (Acharya et al., 2014) |
|  |  |  |  | Methanol with 0.5% MOPS | EHT | 50 |  | 100 |  |  |
|  | *Ernonia anthelmintica*  (L.) Willd./ Purple fleabane/ Herb | Switzerland | Seeds | Ethanol | LMT | 0.010  0.020  0.040  0.080  0.160 |  | 0.00  0.00  12.1  62.8  65.0 | Anthelmintic | (Hördegen et al., 2006) |
|  | *Liatris punctata*Hook./ Dotted gayfeather/ Herb | United states | Roots | Methanol with 0.5% DMSO | EHT | 50 |  | 100 |  | (Acharya et al., 2014) |
|  |  |  |  | Methanol with 0.5% MOPS | EHT | 50 |  | 100 |  |  |
|  | [*Matricaria* *chamomilla* L.](http://www.theplantlist.org/tpl1.1/record/gcc-103038)(=*Chamomilla recutita* (L.) Rausch.)/ Chamomile/ Herb | Slovak Republic | Flowers | Methanol  Aqueous | EHT | 1.024  1.563 |  | 37.5  23.8 | Anti-parasitic | (Váradyová et al., 2018) |
|  | *Matricaria recutita* L./ NA/ Herb | Tunisia | Flower | Aqueous  Methanol  Chloroform  Hexane | EHT | 8 |  | 98  100  70  42 | Antiallergic, antiviral | (Hajaji et al., 2018) |
|  |  |  |  | Aqueous  Methanol  Chloroform  Hexane | AWMT | 8 | 420 | 75.05  91.77  48.07  25.25 |  |  |
|  | *Echinops kebericho* Mesfin*/* Mesfin/ Herb | Ethiopia | Roots | Essential oil | EHT | 0.624  1.25  2.5  5  10 |  | 24.63  41.23  53.67  69.53  81.80 | Diarrhea, migraine, intestinal worm infestation | (Hussien et al., 2011) |
|  | *Eumorphia prostrata* Bolus/ NA/ Shrub |  |  | Ethyle acetate | EHT | 50  25  12.5  6.25  3.125 |  | 88.4  73.2  62.8  41.0  14.6 | Ovicidal and larvicidal activity | (Kamaraj and Rahuman, 2011) |
|  |  |  |  | Acetone |  | 50  25  12.5  6.25  3.125 |  | 83.8  71.6  53.8  27.8  11.8 |  |  |
|  |  |  |  | Methanol |  | 50  25  12.5  6.25  3.125 |  | 100  90.4  65.6  42.2  16.2 |  |  |
|  | *Inula helenium* L./ Elecampane/ Herb | Slovak Republic | Roots | Methanol | EHT | 1.024 |  | 1.7 | Anthelmintic | (Váradyová et al., 2018) |
|  |  |  |  | Aqueous |  | 1.563 |  | 9.9 |  |  |
|  | *Pestasitis hybridus* (L.) G. Gaertn., B. Mey. & Scherb./ Butterbur/ Herb | Slovak Republic | Roots | Methanol | EHT | 1.024 |  | 4.3 |  | (Váradyová et al., 2018) |
|  |  |  |  | Aqueous |  | 1.563 |  | 6.2 |  |  |
|  | *Schkuhria pinnata*(Lam.) Kuntze ex Thell./ NA/ Herb | South Africa | Roots | Acetone | EHT | 2.5 | NA | 37 |  | (Fouche et al., 2016) |
|  | *Solidago virgaurea* L./ Goldenrod/ Herb | Slovak Republic | Stem | Methanol | EHT | 1.024 |  | 4.3 | Antibacterial | (Váradyová et al., 2018) |
|  |  |  |  | Aqueous |  | 1.563 |  | 5.3 |  |  |
|  | *Seriphidium brevifolium* (Wall. ex DC.) Ling & Y.R.Ling  (=*Artemisia brevifolia* Wall. ex DC.)*/*NA/ Herb | Pakistan | Whole plant | Crude aqueous | AWMT | 25 | 360 | 30 | Anthelmintic | (Iqbal et al., 2004) |
|  |  |  |  | Methanol |  |  |  | 80 |  |  |
|  | *Tagetes minuta* L*./*NA/ Herb | Brazil | Leaves | Decoction | EHT | 0.31  0.62  1.25  2.5  5  10 |  | 12.1  44.6  85.8  96.8  100  - |  | (Macedo et al., 2012) |
|  |  |  |  |  | LAEA | 6.20 | 60 | 100 |  |  |
|  | *Vernonia tonoreana* L./NA/ Shrub | Cameroon | Leaves | Crude ethanolic | EHT | 8 | 10080 | 100 |  | (Monglo et al., 2006) |
|  |  |  |  |  | LMT | 8 | 2880 | >85 |  |  |
|  | *Vernonia amygdalina* Delile/ NA/ Shrub | Kenya | Roots | Acetone | AWMT | 6.25  12.5  25 |  | NE | De-wormer | (Sirama et al., 2015) |
|  |  |  |  | Methanol | AWMT | 6.25  12.5  25 |  | 33.3  46.7  56.7 |  |  |
|  |  |  |  | Aqueous | AWMT | 6.25  12.5  25 |  | 20  23.3  26.7 |  |  |
| Bignoniaceae (Bromeliaceae) | *Stereospermum kunthianum* Cham./NA/ Shrub | Cameroon | Leaves | Crude ethanolic | EHT | 8 | 10080 | 84 |  | (Monglo et al., 2006) |
|  |  |  |  |  | LMT | 8 | 2880 | >85 |  |  |
|  | *Ananas comosus*(L.) Merr./ Pineapple/ Herb | South Africa | NA | Ethanol | LMT | 100  200  300 | NA | 98.0  100  100 |  | (Ahmed et al., 2013) |
|  |  | Switzerland | Stem | Ethanol | LMT | 0.010  0.020  0.040  0.060  0.160 |  | 15.3  85.0  80.8  87.9  81.2 | Anthelmintic | (Hördegen et al., 2006) |
| Cactaceae | *Opuntia ficus-indica* (L.) Mill./ NA/ Shrub | Brazil | Fruit | Ethanol | EHT | 3.12  6.25  12.5  25  50  100 |  | 13.0  14.0  24.7  58.2  98.2  100 | Anti-inflammatory, analgesic, antioxidant and antiviral activities. | (Féboli et al., 2016) |
|  |  |  |  | Hexane |  | 3.12  6.25  12.5  25  50  100 |  | 7.5  28.2  26.5  47.0  -  - |  |  |
|  |  |  |  | Dichloromethane |  | 3.12  6.25  12.5  25  50  100 |  | 6.7  15.0  22.5  32.2  -  - |  |  |
|  |  |  |  | Ethyl acetate |  | 3.12  6.25  12.5  25  50  100 |  | 19.2  32.7  58.5  98.7  -  - |  |  |
|  |  |  |  | Residual aqueous |  | 3.12  6.25  12.5  25  50  100 |  | 69.0  84.2  90.0  100  -  - |  |  |
|  |  |  |  | Ethanol | LDA | 1.56  3.12  6.25  12.5  25  50  100 |  | 12.5  49.2  68.5  86.7  96.2  100  100 |  |  |
|  |  |  |  | Hexane |  | 1.56  3.12  6.25  12.5  25  50  100 |  | 86.0  100  100  100  100  -  - |  |  |
|  |  |  |  | Dichloromethane |  | 1.56  3.12  6.25  12.5  25  50  100 |  | 53.2  63.2  68.3  74.5  85.0  -  - |  |  |
|  |  |  |  | Ethyl acetate |  | 1.56  3.12  6.25  12.5  25  50  100 |  | 44.2  67.0  71.1  83.2  85.7  -  - |  |  |
|  |  |  |  | Residual aqueous |  | 1.56  3.12  6.25  12.5  25  50  100 |  | 100  100  100  100  100  -  - |  |  |
| Canellaceae | *Warburgia salutaris*(G.Bertol.) Chiov. / Uganda greenheart/ Herb | South Africa | NA | Ethanol | LMT | 100  200  300 | NA | 76.9  80.8  80.8 |  | (Ahmed et al., 2013) |
|  | *Warburgia ugandensis*Sprague/ Pepper-bark/ Tree | South Africa | NA | Ethanol | LMT | 100  200  300 | NA | 74.1  76.8  81.5 |  | (Ahmed et al., 2013) |
| Capparaceae | *Maerua angolensis*DC./ NA/ Tree | South Africa | Leaves | Acetone | EHT | 2.5 | NA | 25 |  | (Fouche et al., 2016) |
|  |  |  | Stem |  |  | 5 |  | 65 |  |  |
| Caricaceae | *Carica papaya*L.*/* Papaya/ Tree | South Africa | NA | Ethanol | LMT | 100  200  300 | NA | 56.0  64.0  76.0 |  | (Ahmed et al., 2013) |
| Cleomaceae | *Cleome gynandra*L*./*NA/ Shrub | South Africa | Leaves | Acetone | EHT | 5 | NA | 68 |  | (Fouche et al., 2016) |
| Combretaceae | *Anogeissus leiocarpa* (DC.) Guil and Perr (=*Anogeissus leiocarpus* (DC.) Guil and Perr.)/ Axle wood / Tree | Cameroon | Leaves | Crude ethanolic | EHT |  | 10080 | 95 |  | (Monglo et al., 2006) |
|  |  |  |  |  | LMT | 8 | 2880 | >85 |  |  |
|  |  | Nigeria | Leaves | Acetone  Chloroform  Hexane  Butanol  Methanol | LDT | 0.50  0.16  0.18  0.28  0.12 |  | 50  50  50  50  50 | Antiparasitic, anthelmintic | (Ademola and Eloff, 2011) |
|  |  |  |  | Acetone  Chloroform  Hexane  Butanol  Methanol |  |  |  |  |  |  |
|  | *Combretum glutinosum* Perr. Ex D.C./ NA/ Shrub | Benin | Leaves | Acetone | EHT | 2.4 | 2880 | 62.5 | Gastrointestinal disorder, helminth infections(Alowanou et al., 2019) | (Alowanou et al., 2019) |
|  |  |  |  |  | LMT | 1.2 |  | 70.32 |  |  |
|  |  |  |  |  | AMT | 2.4 |  | 100 |  |  |
|  |  |  |  | Methanol | EHT | 2.4 |  | 67.56 |  |  |
|  |  |  |  |  | LMT | 1.2 |  | 71.5 |  |  |
|  |  |  |  |  | AMT | 2.4 |  | 100 |  |  |
|  | *Laguncularia racemosa*(L.) C.F.Gaertn/ NA/ Tree | Mexico | Leaves | Acetone water | EHT | 3.6 |  | 1.90 |  | (Vargas-Magaña et al., 2014) |
|  |  |  |  |  | LMT | 3.6 |  | 47.81 |  |  |
|  | *Terminalia catappa*L./ NA/ Tree | Brazil | Seed | Ethanol | EHT | 0.00248 |  | 50 | Antioxidant, Anti-inflammatory, Anticancer | (Katiki et al., 2017) |
|  |  |  | Pulp |  |  | 0.00463 |  | 50 |  |  |
|  |  |  | Leaves |  |  | 0.02 |  | 50 |  |  |
|  | *Terminalia chebula* Retz.*/* NA/ Tree | India | Leaves | Ethyl acetate | EHT | 50  25  12.5 |  | 84.8  67.8  50.4 | antiviral,  antibacterial and anticancer | (Kamaraj and Rahuman, 2011) |
|  |  |  |  | Acetone |  | 50  25  12.5 |  | 100  92.4  80.4 |  |  |
|  |  |  |  | Methanol |  | 50  25  12.5 |  | 87.4  74.2  63.2 |  |  |
|  |  |  | Seed | Ethyl acetate |  | 50  25  12.5  6.25  3.125 |  | 84.8  67.8  50.4  30.8  13.4 |  |  |
|  |  |  |  | Acetone |  | 50  25  12.5  6.25  3.125 |  | 100  92.4  80.4  55.8  25.0 |  |  |
|  |  |  |  | Methanol |  | 50  25  12.5  6.25  3.125 |  | 87.4  74.2  63.2  48.4  15.4 |  |  |
|  | *Terminalia schimperiana* Hochest. ex Delile/NA/ Tree |  |  | Aqueous  Hydro-alcoholic | EHT | 2  2 | 2880 | 2-3  17-18 | Parasitic skin diseases, fever, jaundice, gonorrhea, | (Eguale et al., 2006) |
|  |  |  |  | Aqueous  Hydro-alcoholic | APMT | 8  8 | 1440 | NE |  |  |
| Cucurbitaceae | [*Cucurbita* *ficifolia* Bouché](http://www.theplantlist.org/tpl1.1/record/kew-2747136) (=*Cucurbita mexicana* Dammann)/ Kuddu/ Herb | Pakistan | Whole fruits | Methanol |  |  | 240 | 83.4 | Anthelmintic activity | (Iqbal et al., 2001b) |
|  | *Cucumis myriocarpus*Naudin/ Wild cucumber/ Herb | South Africa | NA | Ethanol | LMT | 100  200  300 |  | 48.0  56.0  60.0 | NA | (Ahmed et al., 2013) |
| Ericaceae | *Vaccinium macrocarpon*Aiton/ Cranberry vine/ Shrub | United States | Stem, leaves | Aqueous | EHT | 0.3  0.6  1.2  2.5  5.0  10.0 |  | 7  7  10  9  22  39 |  | (Barone et al., 2018) |
|  |  |  |  |  | LMA | 0.3  0.6  1.2  2.5  5.0  10.0 |  | 24  18  14  34  41  88 |  |  |
|  |  |  |  |  | AWMT | 0.3  0.6  1.2  2.5  5.0  10.0 |  | 77  91  97  97  97  97 |  |  |
| Euphorbiaceae  ‎ | *Bridelia ferrugginea* Benth/ NA/ Shrub | Benin | Leaves | Acetone | EHT | 2.4 | 2880 | 70.0 | Gastrointestinal disorder, helminth infections | (Alowanou et al., 2019) |
|  |  |  |  |  | LMT | 1.2 |  | 62.3 |  |  |
|  |  |  |  |  | AMT | 2.4 |  | 100 |  |  |
|  |  |  |  | Methanol | EHT | 2.4 |  | 58.10 |  |  |
|  |  |  |  |  | LMT | 1.2 |  | 73.5 |  |  |
|  |  |  |  |  | AMT | 2.4 |  | 100 |  |  |
|  | *Croton macrostachyus* Hochs.t ex Delile/ NA/Shrub |  |  | Aqueous Hydro-alcoholic | EHT | 0.5  2 | 2880 | 100  98 | Constipation, syphilis, asthma | (Eguale et al., 2006) |
|  |  |  |  | Aqueous  Hydro-alcoholic | APMT | 8  8 | 1240 | 36.67  90 |  |  |
|  | *Croton zehntneri* Pax & K.Hoffm.*/* Canela de cuncha/ Tree |  |  | Essential oi | EHT | 0.31  0.62  1.25 |  | 9.2  12.2  99.9 | Anti-parasitic, anti-bacterial | (Camurça-Vasconcelos et al., 2007) |
|  |  |  |  |  | LDT | 1.25  2.5  5.0  10.0  20.0 |  | 55.8  73.3  81.5  99.2  98.6 |  |  |
|  | *Euphorbia helioscopia* L.*/*Guri sochel/Herb | India | Whole plant | Aqueous | AWMT | 50  25  12.5 | 480 | 82  76  64 | Anthelmintic activity | (Lone et al., 2012) |
|  |  |  |  | Methanol | EHT | 50  25  12.5 |  | 98  92  88 |  |  |
|  |  | India | Whole plant | Crude Aqueous | AWMT | 50  25  12.5 | 480 | 82  76  64 | Anthelmintic and antimicrobial activity | (Lone et al., 2013) |
|  |  |  |  | Crude Methanol | EHT | 50  25  12.5 |  | 98  92  88 |  |  |
|  | *Hura crepitans* L./ Assacu/Tree | Brazil | - | Latex | EHT | 2.5 |  | 16.84 |  | (Carvalho et al., 2012) |
|  |  |  |  |  | LDT | 2.5 |  | 100 |  |  |
|  | *Jatropha curcas* L./ NA/ Shrub | Brazil | Seeds | Ethanol | EHT | 50 |  | 98.8 | Molluscicide, anthelmintic | (Monteiro et al., 2011) |
|  |  |  |  | Ethyl acetate | EHT | 50 |  | 32.2 |  |  |
|  |  |  |  | Hexane | EHT | 50 |  | 15.3 |  |  |
| Fabaceae(=Leguminosae) | *Acacia nilotica* (L.) Delile/NA/ Tree | Ethiopia | Seeds | Aqueous  Hydro-alcoholic | EHT | 2  2 | 2880  1440 | 96  25 | Diabetes, diarrhea, anthelmintic, antiseptic | (Eguale et al., 2006) |
|  | *Acacia cochliacantha*Willd/ Cubata/ Tree | Mexico | Leaves | Hydroalcoholic |  | 25  12.5  6.25  3.12  1.56  0.78 |  | 100  100  100  100  98.0  84.0 | Antiparasitic | (Castillo-Mitre et al., 2017) |
|  |  |  |  | Aqueous |  | 25  12.5  6.25  3.12 |  | 30.50  29.25  16.00  13.00 |  |  |
|  | *Caesalpinia crista* L.*/*Kranjwa/ Herb | Pakistan | Seeds | Aqueous | EHT  AMT | 48 | 360 |  | Anthelmintic activity | (Jabbar et al., 2007) |
|  |  |  |  | Methanol |  |  |  |  |  |  |
|  | *Calpurnia aurea (*Aiton) Benth./ NA/ Tree | South Africa | Leaves | Acetone | EHT | 2.5 | NA | 27 |  | (Fouche et al., 2016) |
|  |  |  | Stem |  |  |  |  | 32 |  |  |
|  | Elephantorrhiza *elephantine* (Burch.) Skeels*/* NA/ Shrub | South Africa | Roots | Aqueous | EHT | 20  10  5  2.5  1.25  0.625 |  | 100  100  100  100  99.3  96.3 |  | (Maphosa et al., 2010) |
|  |  |  |  |  | LDT | 20  10  5  2.5  1.25  0.625 |  | 100  100  100  100  100  98.1 |  |  |
|  |  | South Africa | Roots | Aqueous |  | 0.312  0.625  1.25  2.5  5  10 | 360 | 34.4  44.8  46.0  56.0  56.0  72.2 | Anthelmintic activity | (Maphosa and Masika, 2012) |
|  |  |  |  |  |  | 0.312  0.625  1.25  2.5  5  10 | 1080 | 51.1  54.3  64.3  72.2  67.1  90.3 |  |  |
|  |  |  |  |  |  | 0.312  0.625  1.25  2.5  5  10 | 1800 | 87.8  90.5  95.8  96.3  100  100 |  |  |
|  |  |  |  |  |  | 0.312  0.625  1.25  2.5  5  10 | 2520 | 100  100  100  100  100  100 |  |  |
|  |  |  |  | Ethyl acetate |  | 0.312  0.625  1.25  2.5  5  10 | 360 | 44.4  37.0  37.4  62.7  70.8  77.8 |  |  |
|  |  |  |  |  |  | 0.312  0.625  1.25  2.5  5  10 | 1080 | 77.8  58.9  60.0  84.1  84.7  88.0 |  |  |
|  |  |  |  |  |  | 0.312  0.625  1.25  2.5  5  10 | 1800 | 100  100  100  100  100  100 |  |  |
|  |  |  |  |  |  | 0.312  0.625  1.25  2.5  5  10 | 2520 | 88.9  100  100  100  100  100 |  |  |
|  |  |  |  | Hexane |  | 0.312  0.625  1.25  2.5  5  10 | 360 | 35.6  44.4  44.4  38.9  52.4  51.9 |  |  |
|  |  |  |  |  |  | 0.312  0.625  1.25  2.5  5  10 | 1080 | 35.6  50.0  44.4  44.4  62.7  66.7 |  |  |
|  |  |  |  |  |  | 0.312  0.625  1.25  2.5  5  10 | 1800 | 82.2  88.9  88.9  88.9  89.7  90.7 |  |  |
|  |  |  |  |  |  | 0.312  0.625  1.25  2.5  5  10 | 2520 | 94.4  95.6  100  96.1  100  100 |  |  |
|  | ***Lespedeza cuneata*** (Dum. Cours.) G.DON/ Chinese bush clover/ Herb | South Africa | NA | Ethanolic | LMT | 100  200  300 | NA | 96.1  100  100 | NA | (Ahmed et al., 2013) |
|  | *Leucaena leucocephala* (Lam.) de Wit. / NA/ Shrub | Nigeria | Seeds | Aqueous | LDT | 0.586 ug/ml |  | 50 | Useful in expelling *Ascaris* species worms | (Ademola and Idowu, 2006) |
|  | *Lysiloma latisiliquum*(L.) Benth.*/* NA/ Tree | Mexico | Leaves | Acetone water | EHT | 3.6 |  | 6.35 |  | (Vargas-Magaña et al., 2014) |
|  |  |  |  |  | LMT | 3.6 |  | 19.59 |  |  |
|  | *Melilotus officinalis* subsp. *alba* (Medik.) H. Ohashi & Tateishi )*Melilotus alba* Ledeb.)*/*  White sweet clover/ Shrub | United states | Leaves | Methanol with 0.5% DMSO | EHT | 50 |  | 100 |  | (Acharya et al., 2014) |
|  |  |  |  | Methanol with 0.5% MOPS | EHT | 50 |  | 100 |  |  |
|  | *Melilotus officinalis* (L.) Pall*.*/ Yellow sweet clover/ Herb | United states | Leaves | Methanol with 0.5% DMSO | EHT | 50 |  | 100 |  | (Acharya et al., 2014) |
|  |  |  |  | Methanol with 0.5% MOPS | EHT | 50 |  | 100 |  |  |
|  | *Peltophorum africanum*Sond./ Weeping wattle/ Tree | South Africa | NA | Ethanol | LMT | 100  200  300 | NA | 52.2  60.2  65.2 |  | (Ahmed et al., 2013) |
|  | *Lachesiodendron viridiflorum* (Kunth) P.G.Ribeiro, L.P.Queiroz & Luckow (=*Piptadenia viridiflora* (Kunth) Benth.)/ Surucuc/ Tree | America | Leaves | Aqueous | EHT | 0.075  0.15  0.3  0.6  1.2  2.4 |  | 100  97.85  75.84  44.98  13.16  11.24 |  | (Morais-Costa et al., 2016) |
|  |  |  |  | Ethanol |  | 0.075  0.15  0.3  0.6  1.2  2.4 |  | 100  100  90.9  86.4  74.2  69.6 |  |  |
|  | *Senna italica*Mill./ NA/ Tree | South Africa | Leaves | Acetone | EHT | 5 | NA | 65 |  | (Fouche et al., 2016) |
|  | [*Senna* *occidentalis* (L.) Link](http://www.theplantlist.org/tpl1.1/record/ild-1086). (=*Cassia occidentalis* L.)*/* NA/ Shrub | Uganda | Shoot | Methanolic |  | 25  50  100  2 | 360 | 19.4  36.3  36  94.9 | Inflammations, Diarrhea, Dysentery, Constipation, Fever, Cancer, Eczema, Venereal, Diseases, Helminthosis | (Nsereko et al., 2019) |
|  |  |  |  |  |  | 25  50  100  2 | 1440 | 49.1  54.5  95.7  97 |  |  |
|  | *Tephrosia inandensis*H.M.L.Forbes/ Inanda tephros/ Shrub | South Africa | NA | Ethanol | LMT | 100  200  300 | NA | 24.0  48.0  64.0 | NA | (Ahmed et al., 2013) |
|  | *Caesalpinia* *crista* L./ Nut/ Shrub | Switzerland | Seeds | Ethanol | LMT | 0.002  0.004  0.008  0.016  0.032 |  | 13.2  12.8  24.7  92.1  92.1 | Anthelmintic | (Hördegen et al., 2006) |
| ‎[Gentianaceae](https://en.wikipedia.org/wiki/Gentianaceae) | *Swertia chirata* Buch.-Ham. ex Wall. /Chirayta/ Herb | Pakistan | Whole plant | Methanol |  | 25 | 360 | 100 | Anthelmintic activity | (Iqbal et al., 2006c) |
|  |  |  |  | Aqueous |  |  |  |  |  |  |
| Geraniaceae | *Geranium viscosissimum*Fisch. & C.A.Mey/ Sticky purple geranium/ Herb | Unites states | Leaves | Methanol with 0.5% of DMSO | EHT | 50 | NA | 100 |  | (Acharya et al., 2014) |
|  |  |  |  | Methanol with 0.5% of MOPS | EHT | 50 |  | 55.1 |  |  |
|  | *Monsonia angustifolia*E. Mey. ex A. Rich. */* NA/ Herb | South Africa | Whole plant | Acetone | EHT | 5 | NA | 56 |  | (Fouche et al., 2016) |
|  | *Pelargonium luridum*(Andrews) Sweet/ NA/ Herb | South Africa | Whole plant | Acetone | EHT | 2.5 | NA | 25 |  | (Fouche et al., 2016) |
| Hypoxidaceae | *Hypoxis rigidula*Baker/ NA/ Herb | South Africa | Bulb | Acetone | EHT | 2.5 | NA | 17 | NA | (Fouche et al., 2016) |
| Lamiaceae (=Labiateae) | *Anisomeles* malabarica (L.) R. Br. ex Sims/ NA/ Herb | India | Leaves | Ethyl acetate | EHT | 25  12.5  6.25 |  | 100  89.4  68.8 |  | (Kamaraj et al., 2011) |
|  |  |  |  | Acetone |  | 25  12.5  6.25 |  | 83.2  65.0  39.4 |  |  |
|  |  |  |  | Methanol |  | 25  12.5  6.25 |  | 93.8  78.6  57.2 |  |  |
|  |  |  |  | Ethyl acetate | LDT | 25  12.5  6.25 |  | 100  89.4  75.6 |  |  |
|  |  |  |  | Acetone |  | 25  12.5  6.25 |  | 82.4  65.2  36.8 |  |  |
|  |  |  |  | Methanol |  | 25  12.5  6.25 |  | 92.6  70.0  59.6 |  |  |
|  | *Hyssopus officinalis* L./ Hyssop/ Herb | Slovak Republic | Stem | Methanol  Aqueous | EHT | 1.024  1.563 |  | 3.7  8.3 | Anthelmintic | (Váradyová et al., 2018) |
|  | *Lavandula officinalis* (Chaix & Kitt.)/ NA/ Herb | Brazil | Flowers | Essential oil | EHT | 1.562  3.125  6.25  12.5  25  50 | NA | 74.5  86.4  95.7  97.0  99.4  99.0 | Anthelmintic | (Ferreira et al., 2018) |
|  |  |  |  |  | LDT | 0.937  0.187  0.375  0.75  1.5  3.0 | NA | 46.9  67.1  86.4  97.2  100  100 |  |  |
|  |  |  |  |  | AWMT | 0.5  5  50 | 600  480  480 | 100  100  100 |  |  |
|  | *Leonotis leonurus* (L.) R.Br.*/* Wild dagga/ Shrub | South Africa | Leaves | Aqueous | EHT | 0.625  1.25  2.5  5  10  20 |  | 66  100  100  100  100  100 | Anthelmintic activity | (Maphosa et al., 2010) |
|  |  |  |  |  | LDT | 0.625  1.25  2.5  5  10  20 |  | 97.1  100  100  100  100  100 |  |  |
|  |  | South Africa | NA | Ethanol | LMT | 100  200  300 | NA | 43.5  56.6  56.6 | NA | (Ahmed et al., 2013) |
|  | *Melissa officinalis* L./ Lemon balm/ Herb | Slovak Republic | Stem | Methanol | EHT | 1.024 |  | 4.3 | Anti-parasitic, antibacterial | (Váradyová et al., 2018) |
|  |  |  |  | Aqueous |  | 1.563 |  | 13.2 |  |  |
|  | *Mentha* *×* *villosa* Huds.*/*NA/ Herb | Brazil | Leaves | Decoction | EHT | 0.31  0.62  1.25  2.5  5  10 |  | 24.7  62.7  90.0  97.6  -  - |  | (Macedo et al., 2012) |
|  |  |  |  |  | LAEA | 0.31 |  | 100 (0-60 min) |  |  |
|  | *Ocimum gratissimum* L./ Anchabi/ NA/ Herb | Ethiopia | Leaves | Essential oil | EHT | 0.624  12.5  205  5  10 |  | 51.20  68.93  80.40  93.47  100 | Anthelmintic, digestive disorders | (Hussien et al., 2011) |
|  |  | Nigeria | Leaves | Ethanol | LDA | 1  2  4  8 | 1440 | 1.70  5.00  5.00  15.0 |  | (Njoku and Asuzu, 1998) |
|  |  | Brazil | Leaves | Essential oil | EHT | 10  5  2.5  12.5  0.625 |  | 100.0  100.0  96.94  71.96  57.08 | Anthelmintic activity | (Pessoa et al., 2002) |
|  | *Ocimum lamiifolium* Hotchst. ex Benth/ Damakesee/ Herb | Ethiopia | Leaves | Essential oil | EHT | 0.624  12.5  2.5  5  10 |  | 42.03  55.10  64.13  77.87  92.43 | Intestinal disorders | (Hussien et al., 2011) |
|  |  |  | Infruitscence |  | EHT | 0.624  12.5  2.5  5  10 |  | 28.60  43.97  59.80  75.63  90.50 |  |  |
|  | *Plectranthus punctatus* (L.f) L’Her./ NA/ Herb | Ethiopia | Leaves | Aqueous | EHT | 0.0625  0.125  0.25  0.5  1  2 | 2880 | 15.1  58.7  94.3  100  100  100 | Anthelmintics | (Tadesse et al., 2009) |
|  |  |  |  |  | LDT | 50 | 8640 | 80 |  |  |
|  |  |  |  | Hydro-alcoholic | EHT | 0.0625  0.125  0.25  0.5  1  2 | 2880 | 5.6  13.5  17.7  98.9  100  100 |  |  |
|  |  |  |  |  | LDT | 50 | 8640 | 85 |  |  |
|  | *Rosmarinus officinalis* L./ Rosemary/ Herb | Slovak Republic | Leaves | Methanol | EHT | 1.024 |  | 5.7 | Anthelmintic | (Váradyová et al., 2018) |
|  |  |  |  | Aqueous |  | 1.563 |  | 8.2 |  |  |
|  | *Thymus schimperi* Ronniger/ Tossign/ Herb | Ethiopia | Leaves | Essential oil | EHT | 0.624  1.25  2.5  5  10 |  | 31.10  47.37  59.86  76.77  88.67 | Cough, bronchitis, sore throat, colic, dyspepsia gastritis, flatulence, diarrhea, anthelmintic | (Hussien et al., 2011) |
|  | *Thymus vulgaris* L.*/* NA/ Shrub | Brazil | Leaves | Essential oil | EHT | 50 |  | 100 | Anthelmintic agent | (Ferreira et al., 2016) |
|  |  |  |  |  | LDT | 0.125 |  | 100 |  |  |
|  |  |  |  |  | LMT | 50 |  | 100 |  |  |
|  |  |  |  |  | AWMT | 50  25 |  | 100  98 |  |  |
| Logoniaceae | *Spigelia anthelmia* L./NA/ Herb | Brazil | Arial parts | Hexane | EHT | 50  25  12.5 |  | 46.5  48.9  42.4 |  | (Assis et al., 2003) |
|  |  |  |  | Chloroform |  | 50  25  12.5 |  | 46.7  53.4  31.0 |  |  |
|  |  |  |  | Ethyl acetate |  | 50  25  12.5 |  | 100  83.8  20.9 |  |  |
|  |  |  |  | Methanolic |  | 50  25  12.5 |  | 97.4  59.3  35.0 |  |  |
|  |  |  |  | Hexane | LDT | 50  25  12.5 |  | 45.0  16.4  11.3 |  |  |
|  |  |  |  | Chloroform |  | 50  25  12.5 |  | 36.9  17.2  29.0 |  |  |
|  |  |  |  | Ethyl acetate |  | 50  25  12.5 |  | 81.2  83.1  65.0 |  |  |
|  |  |  |  | Methanolic |  | 50  25  12.5 |  | 84.4  36.1  16.8 |  |  |
| Lythraceae | *Punica granatum* L./ NA/ Shrub | Ethiopia | Peel | Methanol | EHT | 0.1  0.25  0.5  1 |  | 49.3  60.6  72.6  94.6 |  | (Ahmed et al., 2020) |
|  |  |  |  |  | AMT | 1.25  2.5  5  10 |  | 100  90  86  76 |  |  |
|  |  |  | Roots |  | EHT | 0.1  0.25  0.5  1 |  | 46.3  54.3  68.3  90.3 |  |  |
|  |  |  |  |  | AMT | 1.25  2.5  5  10 |  | 90  73  73  70 |  |  |
| Malvaceae | *Abutilon theophrasti* Medik./NA/ Herb | India | Stem | Methanol | EHT | 31.25  62.5  125  250  500 | 1,440 | 47.3  62.4  65.1  71.9  74.3 |  | (Hassan et al., 2019) |
|  |  |  |  | Hexane |  | 31.25  62.5  125  250  500 |  | 48.1  53.1  59.7  64.8  70.0 |  |  |
|  |  |  |  | Aqueous |  | 31.25  62.5  125  250  500 |  | 43.4  59.3  63.9  70.7  72.5 |  |  |
|  |  |  |  | Methanol | LMT | 31.25  62.5  125  250  500 |  | 28.4  49.5  72.3  78.0  79.7 |  |  |
|  |  |  |  | Hexane |  | 31.25  62.5  125  250  500 |  | 19.5  33.1  57.8  70.9  71.2 |  |  |
|  |  |  |  | Aqueous |  | 31.25  62.5  125  250  500 |  | 54.1  61.6  68.4  70.0  75.6 |  |  |
|  | *Althaea officinalis* L./ Marshmallow/ Herb | Slovak Republic | Roots | Methanol | EHT | 1.024 |  | 9.7 | Antibacterial | (Váradyová et al., 2018) |
|  |  |  |  | Aqueous |  | 1.563 |  | 88.3 |  |  |
|  | *Malva sylvestris* L./ Mallow/ Herb | Slovak Republic | Flowers | Methanol | EHT | 1.024 |  | 2.4 | Antibacterial | (Váradyová et al., 2018) |
|  |  |  |  | Aqueous |  | 1.563 |  | 40.4 |  |  |
|  | *Theobroma cacao*L./ NA/ Tree | Maxico | Seeds | Acetone water | EHT | 3.6 |  | 6.54 |  | (Vargas-Magaña et al., 2014) |
|  |  |  |  |  | LMT | 3.6 |  | 50.55 |  |  |
| Meliaceae | *Azadirachta indica* A. Juss. (=*Melia azedarach* L.) / Neem, Syringa/ Tree | Brazil | Ariel parts | Ethanol | EHT | 50  12.5  6.25 |  | 51.3  30.7  4.83 | Acaricide | (Costa et al., 2008) |
|  |  |  |  |  | LDT | 50  25  6.25 |  | 68.1  42.8  35.2 |  |  |
|  |  | South Africa | NA | Ethanolic | LMT | 100  200  300 |  | 48.1  62.9  66.7 | NA | (Ahmed et al., 2013) |
|  |  | Switzerland | Seeds | Ethanol | LMT | 0.003  0.006  0.012  0.024  0.048 |  | 93.1  80.9  91.8  89.2  90.5 |  | (Hördegen et al., 2006) |
|  |  | Brazil | Fruit | Hexane | EHT | 0.00936  0.0187  0.0375  0.075  0.15 |  | 11  13  15  14  18 |  | (Cala et al., 2012) |
|  |  |  |  |  | LDT | 0.00058  0.00117  0.00234  0.00468  0.00936  0.00187 |  | 50  72  80  87.5  89.9  92.2 |  |  |
|  |  | India | Leaves | Aqueous | EHT | 12.5  6.2  3.12  1.56  0.78 |  | 97.8  73  52.2  16.4  9.6 | Ovicidal and larvicidal activity | (Kamaraj et al., 2010) |
|  |  |  |  | Hydro-alcoholic |  | 12.5  6.2  3.12  1.56  0.78 |  | 98.4  84.4  60.4  28.2  8.8 |  |  |
|  |  |  | Seeds | Aqueous | LDT | 12.5  6.2  3.12  1.56  0.78 |  | 100  90.6  58.4  16.2  8.4 |  |  |
|  |  |  |  | Hydro-alcoholic |  | 12.5  6.2  3.12  1.56  0.78 |  | 100  90.2  70.2  18  8.6 |  |  |
|  |  | Brazil | Leaves | Hexane | EHT | 50  25  12.5  6.2  3.12 |  | 16.92  12.93  15.21  15.41  14.45 | Anthelmintic activity | (Maciel et al., 2006) |
|  |  |  |  |  | LDT | 50  25  12.5  6.2  3.12 |  | 67.90  32.08  22.75  13.89  14.25 |  |  |
|  |  |  |  | Ethanol | EHT | 50  25  12.5  6.2  3.12 |  | 100  100  98.24  90.40  66.64 |  |  |
|  |  |  |  |  | LDT | 50  25  12.5  6.2  3.12 |  | 91.64  76.73  67.57  71.7  15.63 |  |  |
|  |  |  | Seeds | Chloroform | EHT | 50  25  12.5  6.2  3.12 |  | 92.39  62.92  54.98  40.60  32.77 |  |  |
|  |  |  |  |  | LDT | 50  25  12.5  6.2  3.12 |  | 93.48  35.13  32.66  26.94  10.44 |  |  |
|  |  |  |  | Ethanol | EHT | 50  25  12.5  6.2  3.12 |  | 100  100  100  100  100 |  |  |
|  |  |  |  |  | LDT | 50  25  12.5  6.2  3.12 |  | 29.03  32.69  29.41  19.08  22.03 |  |  |
|  | *Carapa guianensi*s Aubl./ Andiroba/ Tree | Brazil | Seeds | Oil | EHT | 10 |  | 80.9 |  | (Carvalho et al., 2012) |
|  | *Ekebergia capensis* Sparrm/NA/ Tree | Ethiopia | Seeds | Aqueous  Hydro-alcoholic | EHT | 0.25  2  8  8 | 2880 | 91.8 | Cough, purgative parasitcide,abdominal cramps | (Eguale et al., 2006) |
|  |  |  |  | Aqueous  Hydro-alcoholic | APMT |  | 1440 | 89.2 |  |  |
|  | *Trichilia claussenii* C. DC*./*NA/ Tree | Brazil | Leaves | Methanol | EHT | 0.00936  0.00187  0.00375  0.075  0.15 |  | 4.5  3.8  4.9  4.9  27.2 |  | (Cala et al., 2012) |
|  |  |  |  |  | LDT | 0.00058  0.00117  0.00234  0.00468  0.00936  0.00187 |  | 51  52  72  91.5  92.7  98 |  |  |
|  |  |  |  | Aqueous  Hydro-alcoholic | APMT | 8  8 |  | Not effective |  |  |
| Menispermaceae | *Antizoma angustifolia*(Burch.) Miers ex Harv./ NA/ Shrub | South Africa | Roots | Aetone | EHT | 2.5 | NA | 37 |  | (Fouche et al., 2016) |
| Moraceae | *Ficus abelii*Miq./ Sycamore fig/ Tree | South Africa | NA | Ethanol | LMT | 100  200  300 | NA | 3.10  0.00  6.30 | NA | (Ahmed et al., 2013) |
|  | *Ficus benjamina*L*./* Weeping fig/ Tree | South Africa | NA | Ethanol | LMT | 100  200  300 | NA | 28.1  68.8  78.1 | NA | (Ahmed et al., 2013) |
|  | *Ficus carica*L.*/* Domestic brown fig/ Tree | South Africa | NA | Ethanol | LMT | 100  200  300 | NA | 15.6  53.1  56.1 | NA | (Ahmed et al., 2013) |
|  | *Ficus elastica*Roxb. ex Hornem/ Rubber tree/ Tree | South Africa | NA | Ethanol | LMT | 100  200  300 | NA | 66.7  74.1  77.8 | NA | (Ahmed et al., 2013) |
|  | *Ficus indica* L./ Indian fig/ Shrub | South Africa | NA | Ethanol | LMT | 100  200  300 | NA | 7.49  29.6  44.5 | NA | (Ahmed et al., 2013) |
|  | *Ficus ingens*(Miq.) Miq./ Red-leaved rock fig/ Tree | South Africa | NA | Ethanol | LMT | 100  200  300 | NA | 68.8  75.0  78.1 | NA | (Ahmed et al., 2013) |
|  | *Ficus lutea*Vahl/ Giant-leaved fig/ Tree | South Africa | NA | Ethanol | LMT | 100  200  300 | NA | 40.0  53.4  60.0 | NA | (Ahmed et al., 2013) |
|  | *Ficus natalensis*Hochst./ Natal fig / Tree | South Africa | NA | Ethanol | LMT | 100  200  300 | NA | 62.5  68.6  68.8 | NA | (Ahmed et al., 2013) |
|  | *Ficus ornamental thai/*  Fig species/ Shrub | South Africa | NA | Ethanol | LMT | 100  200  300 | NA | 50.0  60.0  60.0 | NA | (Ahmed et al., 2013) |
|  | *Ficus religiosa* L./ Pippal/ Tree | Pakistan | Bark | Methanol |  |  | 260 | 100 | Anthelmintic activity | (Iqbal et al., 2001a) |
|  | *Ficus sur*Forssk/ Broom cluster fig/ Tree | South Africa | NA | Ethanol | LMT | 100  200  300 | NA | 71.9  75.0  81.3 | NA | (Ahmed et al., 2013) |
| Moringaceae | *Moringa oleifera* Lam./ NA/ Tree | Philippines | Seeds | Ethanol | EHT | 15.6  7.8  3.9 |  | 95.8  71.1  56.8 |  | (Cabardo Jr and Portugaliza, 2017) |
|  |  |  |  | Aqueous |  | 15.6  7.8  3.9 |  | 81.7  68.6  47.6 |  |  |
|  |  |  |  | Ethanolic | LDT | 15.6  7.8  3.9 |  | 56.1  56.9  42.1 |  |  |
|  |  |  |  | Aqueous |  | 15.6  7.8  3.9 |  | 71.6  92.5  32.7 |  |  |
| Musaceae | *Musa x paradisiaca* L.*/* Banana/ Shrub | France | Leaves | Aqueous  Methanol  Dichloromethane | EHT |  |  | Not effective | Antimicrobial | (Marie-Magdeleine et al., 2014) |
|  |  |  |  | Aqueous  Methanol  Dichloromethane | LDT |  |  | 77.4  98.7  51 |  |  |
|  |  |  |  | Aqueous  Methanol  Dichloromethane | AMI |  |  | 12.9  0.0  1 |  |  |
|  |  |  | Seeds | Aqueous  Methanol | EHT |  |  | Not effective |  |  |
|  |  |  |  | Aqueous  Methanol | LDT |  |  | 67.8  95.1 |  |  |
|  |  |  |  | Aqueous  Methanol | AMT |  |  | 3.8  4.8 |  |  |
| Myrtaceae | [*Corymbia* *citriodora* (Hook.) K.D.Hill & L.A.S.Johnson](http://www.theplantlist.org/tpl1.1/record/kew-47992)  (=*Eucalyptus citriodora* Hook.)/ NA/ Tree | Brazil | NA | Essential oil | EHT | 0.125  0.25  0.5  1  2  4 |  | 12.5  12.5  16.0  23.5  58.5  97.1 | antioxidant, antifungal, antibacterial,  anti-inflammatory, analgesic, insect  repellent, insecticide and acaricide | (Ribeiro et al., 2014) |
|  |  |  |  |  | LDT | 0.5  1  2  4  8 |  | 14.4  25.1  47.5  83.8  99.7 |  |  |
|  |  | Brazil | Leaves | Essential oil | EHT | 2  1  0.5 |  | 96.4  73.9  55.4 | Antioxidant, Antifungal, Antibacterial, Anti-Inflammatory, Analgesic, Insecticidal and Acaricidal | (de Araújo-Filho et al., 2018) |
|  |  |  |  |  | LDT | 08  04  02 |  | 93.7  57.1  30.7 |  |  |
|  |  | Brazil | NA | Essential oil |  | 0.75  1  1.25  1.5  1.75  2 | 180 | 29.1  45.8  45.8  62.4  87.5  100 | Anti-bacterial, anti-fungal, insecticidal | (Araújo-Filho et al., 2019) |
|  | *Eucalyptus staigeriana* F.Muell. ex F.M.Bailey*/* NA/ Tree | Brazil | NA | Essential oil | EHT | 0.08  0.16  0.33  0.67  1.35  2.7  5.4 |  | 6.46  8.24  41.54  92.64  99.27  100  100 | Anti-fungal, anti-bacterial, anthelmintic | (Macedo et al., 2010) |
|  |  |  |  |  | LDT | 0.08  0.16  0.33  0.67  1.35  2.7  5.4 |  | -  -  4.26  19.46  32.15  59.54  99.26 |  |  |
|  |  | Brazil |  | Essential oil | EHT | 1.75  0.75  037  018 |  | 99.9  88.6  41.2  20.0 | Acaricide, Insecticides and nematicides | (Ribeiro et al., 2014) |
|  |  |  |  |  | LDT | 1.75  0.75  037  018 |  | 98.4  56.8  18.1  4.69 |  |  |
|  | *Eucalyptus globulus* Labill.*/* NA/ Tree | Brazil | NA | Essential oil | EHT | 21.75 |  | 99.3 |  | (Lara TF et al., 2009) |
|  |  |  |  |  | LDT | 43.5 |  | 98.7 |  |  |
|  | *Melaleuca quinquenervia* (Cav.) S.T. Blake/ NA/ Tree | Cuba | Leaves | Essential oil | EHT | 50  25  12.5 |  | 100  99.4  98.4 | Anti-Inflammatory, Antimalarial, bactericidal, Fungicidal, Larvicidal, repellent | (Gaínza et al., 2015) |
|  |  |  |  |  | LDT | 3.12  1.56  0.78 |  | 100  97.5  89.5 |  |  |
|  | *Psidium cattleianum* Afzel. ex Sabine/ NA/ Tree | Brazil | Leaves | Hydroalcoholic extract | EHT | 0.19  0.39  0.78  1.56  3.12  6.25  12.5  25 |  | 22.1  45.3  56.2  78.3  81.3  98.6  99.4  100 | Anti-inflammatory, Analgesic, Anti-oxidant | (Piza et al., 2019) |
|  |  |  |  |  | NAL | 0.19  0.39  0.78  1.56  3.12  6.25  12.5  25 |  | 49.6  55.7  62.6  61.5  75.8  85.9  90.7  100 |  |  |
|  |  |  |  |  | MLA | 0.19  0.39  0.78  1.56  3.12  6.25  12.5  25 |  | 80.2  94.5  98.0  98.2  98.6  100  100  100 |  |  |
| Oleacea | *Jasminum abysinicum* Hochst. ex DC.*/* Tembelel/ Shrub | Ethiopia | Leaves | Aqueous  Hydro-alcoholic | EHT | 2  2 | 2880 | 51  70 | Anthelmintic | (Getachew et al., 2012) |
|  |  |  |  | Aqueous  Hydro-alcoholic | LDT | 50  50 |  | 67  84 |  |  |
| Palmae (=Arecaceae) | *Cocos nucifera* L./ NA/ Tree | Brazil | - | Ethyl acetate | EHT | 0.31  0.62  1.25  2.5  5 |  | 8.75  10.53  17.12  68.45  100 | Anti-parasitic, anti-bacterial | (Oliveira et al., 2009) |
|  |  |  |  |  | LDT | 5  10  20  40  80 |  | 3.48  2.49  20.71  58.90  99.77 |  |  |
| Papaveraceae | *Fumaria officinalis* L./ Fumitory/ Herb | Slovak Republic | Stem | Methanol  Aqueous | EHT | 1.024  1.563 |  | 2.7  36.6 | Anthelmintic | (Váradyová et al., 2018) |
|  | *Fumaria parviflora*  Lam./ Fumitory/ Herb | Switzerland | Whole plant | Ethanol | LMT | 0.020  0.040  0.080  0.160 |  | 16.1  74.0  67.9  47.5 | Anthelmintic | (Hördegen et al., 2006) |
| Phytolaccaceae | *Phytolacca icosandra* L./ Pokeweed/ Herb | Tropica America | Leaves | Ethanol | LMI | 0.5  1  2  3  4 |  | N.T.  15.1  55.4  28.6  21.8 |  | (Hernández-Villegas et al., 2011) |
|  |  |  |  | n-Hexane |  | 0.5  1  2  3  4 |  | 15.7  22.3  22.3  6.6  N.T. |  |  |
|  |  |  |  | Dichloromethane |  | 0.5  1  2  3  4 |  | 43.1  52.7  56.2  67.1  N.T. |  |  |
|  |  |  |  | Ethanol | EHT | 0.15  0.25  0.35  0.45  0.90  1.8  3.6 |  | 72.6  81.0  81.5  89.7  92.2  95.4  97.5 |  |  |
|  |  |  |  | n-Haxane |  | 0.15  0.25  0.35  0.45  0.90  1.8  3.6 |  | -  -  -  23.0  29.0  40.3  23.2 |  |  |
|  |  |  |  | Dichloromethane |  | 0.15  0.25  0.35  0.45  0.90  1.8  3.6 |  | 33.6  35.1  47.1  74.8  96.7  97.7  99.1 |  |  |
| Piperaceae | *Piper aduncum* L.*/* Pepper jack/ Tree | Brazil | Leaves | Essential oil | EHT | 1.5  3.6  6.0  12.0 |  | 37.2  52.1  87.9  95.0 | Insecticides, larvicides, anti-microbial, molluscicidal, leishmanicidal | (Oliveira et al., 2014) |
|  | *Piper tuberculatum* Jacq./ Pimenta longa/ Shrub | Brazil | Leaves | Crude | EHT |  |  |  |  | (Carvalho et al., 2012) |
|  |  |  |  |  | LDT |  |  |  |  |  |
| Plantaginaceae | *Plantago lanceolata* L./ Ribwort plantain/ Herb | Slovak Republic | Leaves | Methanol | EHT | 1.024 |  | 10.0 | Antibacterial,anthelmintic | (Váradyová et al., 2018) |
|  |  |  |  | Aqueous |  | 1.563 |  | 10.1 |  |  |
| Poaceae (Gramineae) | *Cymbopogon citratus* (DC.) Stapf*/* Lemon grass/ Herb | Brazil | NA | Essential oil | EHT | 0.07  0.15  0.31  0.62  1.25 |  | 27.5  37.1  74.8  96.0  98.4 | Anthelmintic | (Macedo et al., 2019) |
|  |  |  |  | Essential oil nano-emulsion | EHT | 0.07  0.15  0.31  0.62  1.25 |  | 34.9  49.4  58.1  73.2  97.1 |  |  |
|  | *Cymbopogon schoenanthus* (L.) Spreng./ NA/ Herb | Brazil | NA | Essential oils | EHT | 0.18 | 1440  7200  14400  21600 | 97.50  95.00  97.16  98.66 | Sedative, digestive, aromatic properties, and insecticidal activity | (Katiki et al., 2012) |
|  |  |  |  |  |  | 0.36 | 1440  7200  14400  21600 | 95.00  98.80  99.60  98.40 |  |  |
|  |  |  |  |  | LDT | 0.18 | 1440  7200  14400  21600 | 93.33  83.80  84.33  96.83 |  |  |
|  |  |  |  |  |  | 0.36 | 1440  7200  14400  21600 | 71.80  84.80  87.20  96.80 |  |  |
| Primulaceae ( Myrsinaceae) | *Embelia* *ribes* Burm.f./ Black pepper/ Shrub | Switzerland | Fruit | Ethanol | LMT | 0.010  0.020  0.040  0.080  0.160 |  | 15.3  76.7  64.2  85.0  21.8 | Anthelmintic | (Hördegen et al., 2006) |
|  | *Myrsine africana* L.*/* Kechemo/ Shrub | Ethiopia | Leaves | Aqueous  Hydro-alcoholic | EHT | 2  2 | 2880 | 58  52 | Anthelmintic | (Getachew et al., 2012) |
|  |  |  |  | Aqueous  Hydro-alcoholic | LDT | 50  50 |  | 51  89 |  |  |
|  | *Maesa lanceolata* Forssk./ NA/ Shrub | Ethiopia | Leaves | Aqueous | EHT | 0.0625  0.125  0.25  0.5  1  2 | 2880 | 23.7  23.3  86.5  100  100  100 | Anti-parasitic | (Tadesse et al., 2009) |
|  |  |  |  |  | LDT | 50 |  | 85 |  |  |
|  |  |  |  | Hydro-alcoholic | EHT | 0.0625  0.125  0.25  0.5  1  2 |  | 12.1  19.1  67.9  99.4  100  100 |  |  |
|  |  |  |  |  | LDT | 50 |  | 50.3 |  |  |
|  |  |  | Fruit | Aqueous | EHT | 0.0625  0.125  0.25  0.5  1  2 |  | 11  28.9  98.6  99.8  100  100 |  |  |
|  |  |  |  |  | LDT | 50 |  | 78 |  |  |
|  |  |  |  | Hydro-alcoholic | EHT | 0.0625  0.125  0.25  0.5  1  2 |  | 4.7  46.8  98.6  99.7  100  100 |  |  |
|  |  |  |  |  | LDT | 50 |  | 100 |  |  |
| Ranunculaceae | *Aconitum* ferox Wall. ex Ser./NA/ Herb | South Africa | Leaves | Aqueous | EHT | 20  10  5  2.5  1.25  0.625 |  | 100  100  100  100  100  66.0 | Anthelmintic activity | (Maphosa et al., 2010) |
|  |  |  |  |  | LDT | 20  10  5  2.5  1.25  0.625 |  | 100  100  100  100  100  97.1 |  |  |
|  | *Clematis* *brachiata* Thunb./ NA/ Shrub | South Africa | Whole plant | Acetone | EHT | 2.5 |  | 11 |  | (Fouche et al., 2016) |
| Rhizophoraceae | *Rhizophora mangle*L./ NA/ Tree | Maxixo | Leaves | Acetone water | EHT | 3.6 |  | 6.54 |  | (Vargas-Magaña et al., 2014) |
|  |  |  |  |  | LMT | 3.6 |  | 50.55 |  |  |
| Rubiaceae | *Mitragyna inermis* (Willd.) O. Ktze./ NA/ Tree | Benin | Leaves | Acetone | EHT | 2.4 | 2880 | 77.0 | Gastrointestinal disorder, helminth infections | (Alowanou et al., 2019) |
|  |  |  |  |  | LMT | 1.2 |  | 63.6 |  |  |
|  |  |  |  |  | AMT | 2.4 |  | 100 |  |  |
|  |  |  |  | Methanol | EHT | 2.4 |  | 67.5 |  |  |
|  |  |  |  |  | LMT | 1.2 |  | 62.2 |  |  |
|  |  |  |  |  | AMT | 2.4 |  | 100 |  |  |
| Rutaceae | *Citrus aurantifolia* (Christm.) Swingle/ NA/ Tree | Brazil | Fruit peel | Essential oil | EHT | 1.562  3.125  6.25  12.5  25  50 |  | 82.8  92.3  99.4  99.8  100  100 |  | (Ferreira et al., 2018) |
|  |  |  |  |  | LDT | 0.937  0.187  0.375  0.75  1.5  3.0 |  | 11.5  35.9  64.2  81.4  89.8  95.3 |  |  |
|  |  |  |  |  | AWMT | 0.5  5  50 | 840  720  600 | 100  100  100 |  |  |
|  | *Citrus sinensis* (L.) Osbeck/ NA/ Tree | Cuba | Leaves | Essential oil | EHT | 50  25  12.5  6.25 |  | 100  100  100  97.4 | Antioxidant | (Gaínza et al., 2015) |
|  |  |  |  |  | LDT | 3.12  1.56  0.78 |  | 100  81.6  14.8 |  |  |
|  | *Ruta chalepensis* L./ Tenaadam/ Shrub | Ethiopia | Leaves | Essential oil | EHT | 0.624  12.5  2.5  5  10 |  | 48.70  62.07  77.83  91.90  100 | Stomachache | (Hussien et al., 2011) |
|  |  |  | Fruit | Essential oil |  | 0.624  1.25  2.5  5  10 |  | 43.73  58.17  72.67  87.60  99.30 |  |  |
|  | *Zanthoxylum simulans* Hance*/* NA/ Shrub | China | Leaves | Essential oil | EHT | 40.0  20.0  10.0  5.0  2.5  1.25 |  | 100  95.6  75.0  51.4  32.6  20.2 | Anthelmintic activity | (Qi et al., 2015) |
|  |  |  |  | Essential oil | LDT | 40.0  20.0  10.0  5.0  2.5  1.25 |  | 99.8  93.4  84.0  55.4  25.6  18.0 |  |  |
|  |  |  |  | Essential oil | LMT | 40.0  20.0  10.0  5.0  2.5  1.25 |  | 80.4  69.2  59.4  49.6  38.2  21.4 |  |  |
| Simaroubaceae | *Castela tortuosa* Liebm.*/* NA/ Shrub | Mexico | Aerial parts | n-hexane | LDT | 1.25  2.5  5  10  20  40 | 1440 | 8.9  6.6  14.8  17.2  43.9  53.8 |  | (Zamilpa et al., 2019) |
|  |  |  |  |  |  | 1.25  2.5  5  10  20  40 | 4320 | 7.4  22.3  29.8  48.7  78.9  76 |  |  |
| Solanaceae | *Datura* metel L*.*/ NA/ Herb | India | Leaves | Ethyl acetate | EHT | 25  12.5  6.25 |  | 86.6  77.0  62.4 |  | (Kamaraj and Rahuman, 2011) |
|  |  |  |  | Acetone |  | 25  12.5  6.25 |  | 82.4  69.8  54.0 |  |  |
|  |  |  |  | Methanol |  | 25  12.5  6.25 |  | 100  94.6  78.2 |  |  |
|  |  |  |  | Ethyl acetate | LDT | 25  12.5  6.25 |  | 93.8  75.4  58.2 |  |  |
|  |  |  |  | Acetone |  | 25  12.5  6.25 |  | 88.4  67.6  53.0 |  |  |
|  |  |  |  | Methanol |  | 25  12.5  6.25 |  | 100  92.0  81.6 |  |  |
|  | *Nicotiana tabacum* L./ NA/ Herb | Pakistan | Leaves | Crude aqueous | AWMT | 25 | 360 | 77 | Anthelmintic activity | (Iqbal et al., 2006b) |
|  |  |  |  | Methanol |  |  |  |  |  |  |
|  | *Solanum* torvum Sw./ NA/ Shrub | India | Leaves | Ethyle acetate | EHT | 50  25  12.5  6.25  3.125 |  | 100  92.4  74.4  60.6  18.8 | Ovicidal and larvicidal activity | (Kamaraj and Rahuman, 2011) |
|  |  |  |  | Acetone |  | 50  25  12.5  6.25  3.125 |  | 91.4  76.2  68.6  32.4  14.6 |  |  |
|  |  |  |  | Methanol |  | 50  25  12.5  6.25  3.125 |  | 91.2  83.4  60.4  43.6  18.0 |  |  |
|  |  | India | Seeds | Ethyl acetate | EHT | 25  12.5  6.25 |  | 94.8  75.4  52.2 |  | (Kamaraj et al., 2011) |
|  |  |  |  | Acetone |  | 25  12.5  6.25 |  | 91.4  78.0  62.2 |  |  |
|  |  |  |  | Methanol |  | 25  12.5  6.25 |  | 100  93.6  71.2 |  |  |
|  |  |  |  | Ethyl acetate | LDT | 25  12.5  6.25 |  | 93.4  82.4  56.2 |  |  |
|  |  |  |  | Acetone |  | 25  12.5  6.25 |  | 80.6  64.4  48.2 |  |  |
|  |  |  |  | Methanol |  | 25  12.5  6.25 |  | 100  91.4  75.8 |  |  |
| Verbenaceae | *Lantana camara* L.*/* NA/ Shrub | Brazil | Leaves | Decoction | EHT | 0.31  0.62  1.25  2.5  5  10 |  | -  18.3  18.8  14.0  15.7  19.7 |  | (Macedo et al., 2012) |
|  |  |  |  |  | LAEA | 0.62 |  | 100 (0-60 min) |  |  |
|  | *Lippia rugosa* A. Chev./NA/ Herb | Cameroon | Leaves | Crude ethanolic | EHT | 8 | 10080 | 85 |  | (Monglo et al., 2006) |
|  |  |  |  |  | LMT | 8 | 2880 | >85 |  |  |
|  | *Lippia sidoides* Cham./ Alecrim-pimenta/ Shrub | Brazil | Leaves | Essential oil | EHT | 0.625 |  | 100 |  | (Carvalho et al., 2012) |
|  |  |  |  |  | LDT | 0.625 |  | 100 |  |  |
|  |  | Brazil | Leaves | Essential oil | EHT | 0.31  0.62  1.25 |  | 18.8  94.88  100 | Anti-parasitic, anti-bacterial | (Camurça-Vasconcelos et al., 2007) |
|  |  |  |  |  | LDT | 1.25  2.5  5.0  10.0  20.0 |  | 24.9  40.5  64.1  90.2  94.5 |  |  |
| Xanthorrhoeace | *Aloe ferox* Mill./ Bitter Aloe/ Tree | South Africa | Leaves | Aqueous | EHT | 0.625  1.25  2.5  5  10  20 |  | -  33.1  80.7  95.5  98.3  100 |  | (Maphosa et al., 2010) |
|  |  |  |  |  | LDT | 0.625  1.25  2.5  5  10  20 |  | 95.5  97.3  100  100  100  100 |  |  |
|  |  |  |  |  | LAEA | 0.31 |  | 100 (0-60 min) |  |  |
|  |  | South Africa | NA | Ethanol | LMT | 100  200  300 | NA | 73.9  78.3  86.9 | NA | (Ahmed et al., 2013) |
|  | *Aloe rupestris*Baker/ NA/ Shrub | South Africa | Leaves | Acetone | EHT | 2.5 | NA | 47 |  | (Fouche et al., 2016) |
| Zingiberaceae | *Zingiber officinale* Roscoe/ Ginger, Adrak/Herb | Pakistan | Rhizomes | Methanol |  |  | 120 | 100 | Anthelmintic activity | (Iqbal et al., 2001b) |
|  |  | South Africa | NA | Ethanol | LMT | 10  20  30 | NA | 56.0  60.0  72.0 | NA | (Ahmed et al., 2013) |

Supplementary Table 3: *In-vivo* efficacy of medicinal plants against *H. contortus*

| **Scientific/common name/Habit/ Family** | **Part used/ location** | **Extract** | **Dose (mg/kg)** | **Time (days)** | **Mode of administration** | **Animal model** | **Age (months)** | **Inhibition effect (%age)** | **Reference** |
| --- | --- | --- | --- | --- | --- | --- | --- | --- | --- |
| *Dysphania* *ambrosioides* (L.) Mosyakin & Clemants (*=Chenopodium ambrosioides* L.)*/* Mexican tea/ Herb / Amaranthaceae | Aerial parts/ Mexico | n-hexane | 40 | NA | Oral | Gerbils | NA | 45.8. | (Zamilpa et al., 2019) |
| *Allium sativum* L.*/* Garlic/ Bulb/ Amaryllidaceae | Peel/ Mexico | n-hexane | 40 | 13 | Oral | Gerbils | 3 months | 68.7 | (Palacios-Landín et al., 2015) |
| *Anacardium occidentalis* L./ Cashew apple/ Tree/ Anacardiaceae | Fiber/ Brazil | NA | 0.3^*^ | 28 | Oral | Santa Ines sheep | 8 | 40.8 | (Lopes et al., 2018) |
| *Schinopsis spp./* Quebracho/ Tree/ Anacardiaceae | NA/ France | Drench | 0.9 | 8 | Oral | Goats | NA | Caused vacuolization of intestinal, muscular, and hypodermal cells. | (Martínez-Ortiz-de-Montellano et al., 2019) |
| *Annona squamosa* L.*/* Sitaphal/ Tree/ Annonaceae | Leaves/ India | Aqueous | 1500 | 10 | Oral | Goats | NA | No significant reduction of FEC and showed poor anti-parasitic activity. | (Dixit et al., 2019) |
|  | Leaves/ Sweden | Aqueous | 1000 | 21 | Oral | Lambs | 3-6 months | No significant effect on FEC and TWC | (Githiori et al., 2004) |
| *Coriandrum sativum* L./ NA/ Herb/ Apiaceae | Seeds/ Ethiopia | Aqueous | 450 | 14 | Oral | Menz sheep | 6-8 | 24.79 | (Eguale et al., 2007b) |
| *Calotropis procera* (Aiton) Daryand./ Usher/ Shrub/ Apocynaceae | Latex/ Saudi Arabia | NA | 20 | Single-dose | Oral | Lambs | 6-8 | Reduced the egg production and after necropsies, few adult *H. contortus* were found. | (Al-Qarawi et al., 2001) |
|  | Flower/ Pakistan | Crude aqueous | 0.003 | 7 | Oral | Sheep |  | 88.4 | (Iqbal et al., 2005) |
|  |  | Crude powder |  | 10 |  |  |  | 77.8 |  |
|  |  | Methanol |  | 7 |  |  |  | 20.9 |  |
| *Hedera helix* L./ NA/ Climber/ Araliaceae | Fruit/ Ethiopia | Aqueous | 2250 | 14 | Oral | Indigenous sheep | 6-8 | 36.07 | (Eguale et al., 2007a) |
| *Achillea millefolium* L./ Pehl-ghasa/ Herb/ Asteraceae (=Compositae) | Whole plant/ India | Crude aqueous | 2 | 15 | Oral | Sheep | NA | 88.40 | (Tariq et al., 2008) |
|  |  | Crude ethanolic | 2 | 15 | Oral |  |  | 76.53 |  |
| *Artemisia absinthium* L.*/* Wormwood/ Herb/ Asteraceae | Whole plant/ India | Crude ethanolic | 2000 | 15 | Oral | Sheep | NA | 90.46% | (Tariq et al., 2009) |
|  |  | Crude aqueous | 2 | 15 | Oral |  |  | 82.85 |  |
|  |  | Crude ethanolic | 2 | 15 | Oral |  |  | 76.53 |  |
|  | Seeds/ USA | Ethanolic | 100 | 5 | Oral | Gerbils | 1.1 | No significant effect was observed. | (Squires et al., 2011) |
| *Artemisia annua* L.*/* Sweet Annie/ Herb/ Asteraceae | Leaves/ USA | Ethanolic | 600  1000 | 5 | Oral | Gerbils | 1.1 | No significant effect was observed. | (Squires et al., 2011) |
|  |  | Aqueous | 600 |  |  |  |  |  |  |
|  |  | Essential oil | 300 |  |  |  |  |  |  |
| *Artemisia herba-alba* Asso/ NA/ Shrub/ Asteraceae | Shoots/ NA | NA | 0.003 |  | Oral | Sheep | NA | Absence of eggs in feces, adult worms in the abomasum, and significant lesions in the tissues. | (Idris et al., 1982) |
| *Artemisia maritima* L./NA/Herb/ Asteraceae | Whole plant/ Pakistan | Methanolic | 50 | 28 | Oral | Sheep | 8-12 months | 82.22 | (Irum et al., 2015) |
| *Artemisia vestita* Wall. ex Besser / NA/ Shrub/ Asteraceae | Whole plant/ Pakistan | Methanolic | 50 | 28 | Oral | Sheep | 8-12 months | 86.35 | (Irum et al., 2015) |
| *Seriphidium brevifolium* (Wall. ex DC.) Ling & Y.R.Ling  (=*Artemisia brevifolia* Wall. ex DC.)*/*NA/ Herb/ Asteraceae | Pakistan | Whole plant | 0.003 | 14 | Oral | Sheep | ≤1 year | 67.2  62.1 | (Iqbal et al., 2004) |
| *Tagetes erecta* L./ Marigold/ Herb/ Asteraceae | Flower/ Mexico | n-hexane | 40 | 13 | Oral | Gerbils | 3 months | 53.9 | (Palacios-Landín et al., 2015) |
| *Ananas comosus* (L.) Merr./ Pineapple/ Herb/ Bromeliaceae | Skin/ Brazil | Aqueous | 2000 | 3 | Oral | Santa Ines sheep | 4 | 22.6 | (Domingues et al., 2013) |
|  | Leaves/ Sweden | Aqueous | 1000 | 21 | Oral | Lambs | 3-6 months | No significant effect on FEC and TWC | (Githiori et al., 2004) |
| *Hildebrandtia sepalosa* Rendle / NA/ Shrub/ Convolvulaceae | Roots, bark/ Sweden | Aqueous | 1600 | 21 | Oral | Lambs | 3-6 months | No significant effect on FEC | (Githiori et al., 2004) |
| *Euphorbia helioscopia* L.*/* Guri sochel/ Herb/ Euphorbiaceae | Whole plant/ India | Aqueous | 5 | 18 | Oral | Kashmir Marino Sheep | 12 | 86.07 | (Lone et al., 2012) |
|  |  | Methanol |  |  |  |  |  | 44.15 |  |
| *Acacia pubescene* (Vent.) R.Br. (=*Acacia mollissima* Willd. */* NA/ Tree/ Fabaceae (Leguminosae) | NA/ Brazil | Condensed tannin (CT) | 0.0016 | 60 | Oral | Santa Ines | 3 months | Reduced FEC and worm burden | (Minho et al., 2008) |
| *Lysiloma latisiliquum* (L.) Benth.*/* Tzalam/ Tree/ Fabaceae | Leaves/ Mexico | Forage | 0.8 | 8 | Oral | Goats | NA | Caused vacuolization of intestinal, muscular, and hypodermal cells. | (Martínez-Ortiz-de-Montellano et al., 2019) |
| *Onobrychis viciifolia* Scop.*/* Sainfoin hay/ Herb/ Fabaceae | NA/ France | Suspension | 0.9 | 8 | Oral | Goats | NA | Caused vacuolization of intestinal, muscular, and hypodermal cells. | (Martínez-Ortiz-de-Montellano et al., 2019) |
|  | Whole plant/ Switzerland | NA | NA | 16 | Oral | Naïve lambs | 2.5-3 months | 47 (adult worms)  58 (FEC)  49 (adult worms)  48 (FEC) | (Heckendorn et al., 2006) |
| *Lachesiodendron viridiflorum* (Kunth) P.G.Ribeiro, L.P.Queiroz & Luckow (=*Piptadenia viridiflora* (Kunth) Benth.)/ Surucucu/ Tree/ Fabaceae | Leaves/ South Africa | Aqueous | 283 | 21 | Oral | Lambs | 4-8 | Fecal egg count was significantly reduced | (Morais-Costa et al., 2016) |
| *Prosopis laevigata* (Willd.) M.C. Johnst./ Mezquite/ Tree/ Fabaceae | Leaves/Mexico | n-hexane | 40 | 5 | Intraperitoneal | Gerbil | 5 weeks | 42.5 | (De Jesús-Gabino et al., 2010) |
| *Cymbopogon schoenanthus* (L.) Spreng./ Herb/ Fabaceae | NA/ Brazil | Essential oil | 360 | 20 | Oral | Santa Ines lambs | 2 | No anthelmintic activity at the tested doses. | (Katiki et al., 2012) |
| *Cymbopogon citratus* (DC.) Stapf*/* Lemon grass/ Herb/ Gramineae (=Poaceae) | NA/ Brazil | Essential oil | 50 | 8  15 | Oral | Sheep | 10-16 | 19.5  23.9 | (Macedo et al., 2019) |
| *Spigelia anthelmia* L./ wormgrass/ Herb/ Loganiaceae | Whole plant/ Nigeria | Aqueous crude | 500 | 12 | Oral | Weaned lambs | 4-8 | Significantly reduced fecal egg account | (Ademola et al., 2007) |
| *Azadirachta indica* A. Juss. (=*Melia azadirachta* L)/ Neem/ Tree/ Meliaceae | Seeds/Pakistan | Crude aqueous methanol | 4000 | 14 | Oral | Ram and ewe lambs | 3-6 | 85.24 | (Hamad et al., 2014) |
|  | Leaves/ Brazil | NA | 100 | 90 | Oral | Sheep | 6-12 | Revealed no significant anthelmintic activity. | (Costa et al., 2008) |
|  | Leaves/ India | Aqueous | 1000 | 10 | Oral | Goats | NA | No anti-parasitic activity. | (Dixit et al., 2019) |
|  | Leaves/ Sweden | Aqueous | 500 | 21 | Oral | Lambs | 3-6 months | No significant effect on FEC | (Githiori et al., 2004) |
| *Khaya senegalensis* (Desv.) A.Juss./ NA/ Tree/ Meliaceae | Bark/ Nigeria | Ethanolic | 500 | 12 | Oral | Weaned lambs | 4-8 | 88.82 | (Ademola et al., 2004) |
| *Eucalyptus staigeriana* F. Muell. ex F.M. Bailey*/* NA/ Tree/ Myrtaceae | NA/ Brazil | EncEO, EsEO | 500 | 4 | Oral | Mongolian gerbil (*Meriones* *unguiculatus*) | 1.3 | 40.51  46.44 | (Ribeiro et al., 2014) |
| [*Corymbia* *citriodora* (Hook.) K.D.Hill & L.A.S.Johnson](http://www.theplantlist.org/tpl1.1/record/kew-47992)**.** (=*Eucalyptus citriodora* Hook.)*/* NA/ Tree/ Myrtaceae | NA/ Brazil | Essential oil | 2 | 0.125  0.25  0.5 | Oral | Sheep | 7-16 | 100 | (Araújo-Filho et al., 2019) |
| *Olea europaea* (=*Olea europaea* var. *africana*)/ NA/ Tree | Bark/ Sweden | Aqueous | 2000 | 21 | Oral | Lambs | 3-6 months | No significant effect on FEC | (Githiori et al., 2004) |
| *Cocos nucifera* L./ NA/ Tree/ Palmae (=Arecaceae) | Fruit/ Brazil | Ethyl acetate | 400 | 3 | Oral | Sheep | 12 | 4.77 | (Oliveira et al., 2009) |
| *Hagenia abyssinica* (Bruce ex Steud.) J.F.Gmel. / NA/ Tree/ Rosaceae | Inflorescence/ Sweden | Aqueous | 1000 | 21 | Oral | Lambs | 3-6 months | No significant effect on FEC | (Githiori et al., 2004) |
| *Dodonaea viscosa* subsp. *angustifolia* (L.f.) J.G.West (=*Dodonea angustifolia* L.f.)/ NA/ Tree/ Sapindaceae | Leaves/ Sweden | Aqueous | 1000 | 21 | Oral | Lambs | 3-6 months | No significant effect on FEC | (Githiori et al., 2004) |
| *Castela tortuosa* Liebm.*/* NA/ Shrub/ Simaroubaceae | Aerial parts/ Mexico | n-hexane | 40 | NA | Oral | Gerbils | NA | 27.1 | (Zamilpa et al., 2019) |
| *Nicotiana tabacum* L./ Tobaco/ Herb/ Solanaceae | Leaves/Pakistan | Crude aqueous methanol | 4000 | 14 | Oral | Ram and ewe lambs | 3-6 | 86.6 | (Hamad et al., 2014) |
|  | Leaves/ India | Aqueous | 1000 | 10 | Oral | Goats | NA | No significant anti-parasitic activity. | (Dixit et al., 2019) |
| *Lippia sidoides* Cham./ Shrub/ Verbenaceae | NA/ Brazil | Essential oil | 283 | 14  21 | Oral | Sheep | NA | 39.5  56.9 | (Camurça-Vasconcelos et al., 2007) |
| *Balanites aegyptiaca* (L.) Delile. / Desert date/ Tree/ Zygophyllaceae (=Balanitaceae) | Fruit/ Egypt | Ethanolic | 9 | 28 | Oral | Male Baladi Goats | 6-9 | 88.10 (EPG)  94.66 (worm burden) | (Jaheed et al., 2019) |

Keys: NA= Data not available; EncEO= Encapsulated essential oil; EsEO= *Eucalyptus staigeriana* essential oil; FECR%= Feacal egg count reduction %; EPG= egg count per gram of feces; WCT= total worm count reduction

^*^ indicates % BW

Supplementary Table 4: Toxicology of different plants species

| **Species** | **Concentration (mg/kg)** | **Exposure time** | **Mode of administration** | **Model/cell line** | **Toxicity level** | **LC­_50_ value** | **Physiological changes** | **References** |
| --- | --- | --- | --- | --- | --- | --- | --- | --- |
| *Allium sativum* L. | NA | NA |  | Human HaCat cells | Moderate | 22.27±1.61 µg/mL | NA | (Krstin et al., 2018) |
| *Ananas comosus* (L.) Merr. | 750 | NA | Oral | NA | Nil | NA | Safe, without any adverse effects | (Maurer, 2001) |
|  | 5000 | 14 days | Oral | Sprague-Dawley rats | Non | >5000 mg/kg | No alteration in body | (Dutta and Bhattacharyya, 2013) |
| *Achillea millefolium* L. | 0.01  0.003 | 30-90 days | Oral | Wistar rats | Nil | NA | Non-significant change in blood glucose, cholesterol levels, and liver weight. | (Cavalcanti et al., 2006) |
| *Artemisia absinthium* L. | NA | NA | NA | NA | Toxic | NA | Long-term use leads to neurotoxic effects. | (Lachenmeier, 2010) |
| *Artemisia herba-alba* Asso | 300 | 4-12 weeks | Oral | Female Sprague-Dawley rats | Toxic | NA | Effects on the fertility and reproductive system | (Almasad et al., 2007) |
| *Azadirachta indica* L. | 18.4-45 | 14-28 days | Oral | Mice | Toxic | 31.95g/kg | Histopathological analysis at 1600mg/kg showed that the testicles, liver, and kidney are the target organs. | (Deng et al., 2013) |
| *Calotropis procera* (Aiton.) W.T.Aiton | 0.001-6 | 1-14 days | Oral, intravenous, intraperitoneal, subcutaneous | Sheep, goats | Toxic | NA | Signs of nervousness, salivation, urination, dyspnea, tachycardia, and loss of condition. Morbidity in intestines, heart, liver, kidneys, lungs, brain. | (Mahmoud et al., 1979) |
| *Coriandrum sativum* L. | 1000-5000 | 28 days | Oral | Mice | Nil | >5000 mg/kg | No damage to vital organs | (Patel et al., 2012) |
| *Cocos nucifera* L. | 2000 | 8 days | Intramuscular | Mice | Toxic | 1233.9 mg/kg | NA | (Tayler et al., 2020) |
| *Corymbia citriodora* (Hook.) K.D.Hill & L.A.S. Johnson | 175; 440; 1,100; 2,800; 5000 | 48 hours | Oral | Swiss albino mice | Slight toxic | 5,000  2,609 | Mortality effects | (Araújo-Filho et al., 2019) |
| *Cymbopogon citratus* (DC.) Stapf | 0.0312 | 72 hours | NA | Mouse peritoneal macrophages | Nil | NA | NA | (Santoro et al., 2007) |
| *Cymbopogon schoenanthus* (L.) Spreng. | 180; 360 | 5, 10, 20 days | Oral | Lambs | Nil | NA | No significant changes | (Katiki et al., 2012) |
| *Eucalyptus staigeriana* F. Muell. ex F.M. Bailey | 1000-5000  200-600 | 6 hours  30 days | Oral  Intraperitoneal | Swiss albino mice | Nil | 4,112.94 mg/kg  408.95 mg/kg | No significant difference | (Macedo et al., 2010) |
| *Euphorbia helioscopia* L. | 2000 | 6 hours  14 days | Oral | Swiss albino mice | Nil | NA | No physiological alteration | (Saleem et al., 2016) |
| Lippia origanoides Kunth | NA | NA | NA | Mammalian macrophage cells | Low | 192.7 µg/mL | Low toxicity was observed | (de Melo et al., 2020) |
| *Nicotiana tabacum* L. | 5000 | 14 days | Oral | Winstar rats | Nil | NA | Not toxic | (Andjani et al., 2019) |
| *Lachesiodendron viridiflorum* (Kunth) P.G.Ribeiro, L.P.Queiroz & Luckow | 203.0 | 4 days | Oral | Mice | Nil | NA | Not toxic | (Morais-Costa et al., 2016) |
| *Spigelia anthelmia* L. | 5000 | 14 days | Oral | Swiss albino mice | Nil | NA | Not toxic | (Ribeiro et al., 2017) |

Key: NA, Data not available

**References**

Acharya, J., Hildreth, M. B., Reese, R. N. (2014). In vitro screening of forty medicinal plant extracts from the United States Northern Great Plains for anthelmintic activity against *Haemonchus contortus*. *Vet Parasitol* 201(1-2), 75-81.

Ademola, I., Eloff, J. N. (2011). Ovicidal and larvicidal activity of *Cassia alata* leaf acetone extract and fractions on *Haemonchus contortus*: In vitro studies. *Pharm Biol* 49(5), 539-544.

Ademola, I., Fagbemi, B., Idowu, S. (2004). Evaluation of the anthelmintic activity of *Khaya senegalensis* extract against gastrointestinal nematodes of sheep: in vitro and in vivo studies. *Vet Parasitol* 122(2), 151-164.

Ademola, I., Fagbemi, B., Idowu, S. (2007). Anthelmintic activity of *Spigelia anthelmia* extract against gastrointestinal nematodes of sheep. *Parasitol Res* 101(1), 63-69.

Ademola, I., Idowu, S. (2006). Anthelmintic activity of *Leucaena leucocephala* seed extract on *Haemonchus contortus*-infective larvae. *Vet Rec* 158(14), 485.

Ahmed, A. H., Ejo, M., Feyera, T., Regassa, D., Mummed, B., Huluka, S. A. (2020). In Vitro Anthelmintic Activity of Crude Extracts of *Artemisia herba-alba* and *Punica granatum* against *Haemonchus contortus*. *J Parasitol Res*, 4950196. doi: 10.1155/2020/4950196

Ahmed, M., Laing, M., Nsahlai, I. (2013). In vitro anthelmintic activity of crude extracts of selected medicinal plants against *Haemonchus contortus* from sheep. *J Helminthol* 87(2), 174-179.

Al-Qarawi, A., Mahmoud, O., Sobaih, M., Haroun, E., Adam, S. (2001). A preliminary study on the anthelmintic activity of *Calotropis procera* latex against Haemonchus contortus infection in Najdi sheep. *Vet Res Commun* 25(1), 61-70.

Al-Shaibani, I., Phulan, M., Arijo, A., Qureshi, T. (2008). Ovicidal and larvicidal properties of *Adhatoda vasica* (L.) extracts against gastrointestinal nematodes of sheep in vitro. *Pak Vet J* 28(2), 79-83.

Almasad, M. M., Qazan, W. S., Daradka, H. (2007). Reproductive toxic effects of *Artemisia herba alba* ingestion in female Spague-Dawley rats. *Pak J Biol Sci* 10(18), 3158-3161.

Alowanou, G. G., Olounladé, P. A., Akouèdegni, G. C., Faihun, A. M. L., Koudandé, D. O., Hounzangbé-Adoté, S. (2019). In vitro anthelmintic effects of *Bridelia ferruginea, Combretum glutinosum*, and *Mitragyna inermis* leaf extracts on *Haemonchus contortu*s, an abomasal nematode of small ruminants. *Parasitol Res* 118(4), 1215-1223. doi: 10.1007/s00436-019-06262-5

Andjani, H., Sentosa, Y., Yati, K., Fauzantoro, A., Gozan, M., Yoo, Y. (2019). *Acute Oral Toxicity Test of Nicotiana tabacum L. Bio-Oil Against Female Winstar Rats.* Paper presented at the IOP Conference Series: Earth and Environmental Science.

Araújo-Filho, J. V., Ribeiro, W. L. C., André, W. P. P., Cavalcante, G. S., Rios, T. T., Schwinden, G. M., et al. (2019). Anthelmintic activity of *Eucalyptus citriodora* essential oil and its major component, citronellal, on sheep gastrointestinal nematodes. *Rev Bras Parasitol Vet* 28(4), 644-651. doi: 10.1590/s1984-29612019090

Assis, L., Bevilaqua, C., Morais, S., Vieira, L., Costa, C., Souza, J. (2003). Ovicidal and larvicidal activity in vitro of *Spigelia anthelmia* Linn. extracts on *Haemonchus contortus*. *Vet Parasitol* 117(1-2), 43-49.

Barone, C. D., Zajac, A. M., Manzi-Smith, L. A., Howell, A. B., Reed, J. D., Krueger, C. G., et al. (2018). Anthelmintic efficacy of cranberry vine extracts on ovine *Haemonchus contortus*. *Vet Parasitol* 253, 122-129.

Cabardo Jr, D. E., Portugaliza, H. P. (2017). Anthelmintic activity of *Moringa oleifera* seed aqueous and ethanolic extracts against *Haemonchus contortus* eggs and third stage larvae. *Int J Vet Sci Med* 5(1), 30-34.

Cala, A., Chagas, A., Oliveira, M., Matos, A., Borges, L., Sousa, L., et al. (2012). In vitro anthelmintic effect of *Melia azedarach* L. and *Trichilia claussenii* C. against sheep gastrointestinal nematodes. *Exp Parasitol* 130(2), 98-102.

Camurça-Vasconcelos, A., Bevilaqua, C., Morais, S., Maciel, M., Costa, C., Macedo, I., et al. (2007). Anthelmintic activity of *Croton zehntneri* and *Lippia sidoides* essential oils. *Vet Parasitol* 148(3-4), 288-294.

Carvalho, C. O., Chagas, A. C. S., Cotinguiba, F., Furlan, M., Brito, L. G., Chaves, F. C., et al. (2012). The anthelmintic effect of plant extracts on *Haemonchus contortus* and *Strongyloides venezuelensis*. *Vet Parasitol* 183(3-4), 260-268.

Castillo-Mitre, G., Olmedo-Juárez, A., Rojo-Rubio, R., González-Cortázar, M., Mendoza-de Gives, P., Hernández-Beteta, E., et al. (2017). Caffeoyl and coumaroyl derivatives from *Acacia cochliacantha* exhibit ovicidal activity against *Haemonchus contortus*. *J Ethnopharmacol* 204, 125-131.

Cavalcante, G. S., de Morais, S. M., Andre, W. P., Ribeiro, W. L., Rodrigues, A. L., De Lira, F. C., et al. (2016). Chemical composition and in vitro activity of *Calotropis procera* (Ait.) latex on *Haemonchus contortus*. *Vet Parasitol* 226, 22-25.

Cavalcanti, A. M., Baggio, C. H., Freitas, C. S., Rieck, L., de Sousa, R. S., Da Silva-Santos, J. E., et al. (2006). Safety and antiulcer efficacy studies of *Achillea millefolium* L. after chronic treatment in Wistar rats. *J Ethnopharmacol* 107(2), 277-284.

Cortes-Morales, J. A., Olmedo-Juárez, A., Trejo-Tapia, G., González-Cortazar, M., Domínguez-Mendoza, B. E., Mendoza-de Gives, P., et al. (2019). In vitro ovicidal activity of *Baccharis conferta* Kunth against *Haemonchus contortus*. *Exp Parasitol* 197, 20-28. doi: 10.1016/j.exppara.2019.01.003

Costa, C., Bevilaqua, C., Camurça-Vasconcelos, A., Maciel, M., Morais, S., Castro, C., et al. (2008). In vitro ovicidal and larvicidal activity of *Azadirachta indica* extracts on *Haemonchus contortus*. *Small Rumin Res* 74(1-3), 284-287.

de Araújo-Filho, J. V., Ribeiro, W. L., André, W. P., Cavalcante, G. S., de CM Guerra, M., Muniz, C. R., et al. (2018). Effects of *Eucalyptus citriodora* essential oil and its major component, citronellal, on *Haemonchus contortus* isolates susceptible and resistant to synthetic anthelmintics. *Ind Crops Prod* 124, 294-299.

De Jesús-Gabino, A., Mendoza-de Gives, P., Salinas-Sánchez, D., López-Arellano, M. E., Liébano-Hernández, E., Hernández-Velázquez, V., et al. (2010). Anthelmintic effects of *Prosopis laevigatan*-hexanic extract against *Haemonchus contortus* in artificially infected gerbils (*Meriones unguiculatus*). *J Helminthol* 84(1), 71-75.

de Melo, A. R. B., Higino, T. M. M., da Rocha Oliveira, A. D. P., Fontes, A., da Silva, D. C. N., de Castro, M. C. A. B., et al. (2020). *Lippia sidoides* and *Lippia origanoides* essential oils affect the viability, motility and ultrastructure of *Trypanosoma cruzi*. *Micron* 129, 102781.

de Oliveira, L. M. B., Bevilaqua, C. M. L., Macedo, I. T. F., de Morais, S. M., Machado, L. K. A., Campello, C. C., et al. (2011). Effects of *Myracrodruon urundeuva* extracts on egg hatching and larval exsheathment of *Haemonchus contortus*. *Parasitol Res* 109(3), 893.

Deng, Y.-x., Cao, M., Shi, D.-x., Yin, Z.-q., Jia, R.-y., Xu, J., et al. (2013). Toxicological evaluation of neem (*Azadirachta indica*) oil: acute and subacute toxicity. *Environ Toxicol Pharmacol* 35(2), 240-246.

Dixit, A. K., Das, G., Dixit, P., Sharma, R. L. (2019). Efficacy of herbal extracts and closantel against fenbendazole-resistant *Haemonchus contortus*. *J Helminthol* 93(5), 529-532. doi: 10.1017/s0022149x18000627

Domingues, L. F., Giglioti, R., Feitosa, K. A., Fantatto, R. R., Rabelo, M. D., de Sena Oliveira, M. C., et al. (2013). In vitro and in vivo evaluation of the activity of pineapple (*Ananas comosus*) on *Haemonchus contortus* in Santa Inês sheep. *Vet Parasitol* 197(1-2), 263-270.

Dutta, S., Bhattacharyya, D. (2013). Enzymatic, antimicrobial and toxicity studies of the aqueous extract of *Ananas comosus* (pineapple) crown leaf. *J Ethnopharmacol* 150(2), 451-457.

Eguale, T., Tilahun, G., Debella, A., Feleke, A., Makonnen, E. (2007a). *Haemonchus contortus*: in vitro and in vivo anthelmintic activity of aqueous and hydro-alcoholic extracts of *Hedera helix*. *Exp Parasitol* 116(4), 340-345.

Eguale, T., Tilahun, G., Debella, A., Feleke, A., Makonnen, E. (2007b). In vitro and in vivo anthelmintic activity of crude extracts of *Coriandrum sativum* against *Haemonchus contortu*s. *J Ethnopharmacol* 110(3), 428-433.

Eguale, T., Tilahun, G., Gidey, M., Mekonnen, Y. (2006). In vitro anthelmintic activities of four Ethiopian medicinal plants against *Haemonchus contortus*. *Pharmacologyonline* 3, 153-165.

Féboli, A., Laurentiz, A. C., Soares, S. C., Augusto, J. G., Anjos, L. A., Magalhães, L. G., et al. (2016). Ovicidal and larvicidal activity of extracts of *Opuntia ficus-indica* against gastrointestinal nematodes of naturally infected sheep. *Vet Parasitol* 226, 65-68.

Ferreira, L., Castro, P., Chagas, A., França, S., Beleboni, R. (2013). In vitro anthelmintic activity of aqueous leaf extract of *Annona muricata* L.(Annonaceae) against *Haemonchus contortus* from sheep. *Exp Parasitol* 134(3), 327-332.

Ferreira, L. E., Benincasa, B. I., Fachin, A. L., Contini, S. H. T., França, S. C., Chagas, A. C. S., et al. (2018). Essential oils of *Citrus aurantifolia, Anthemis nobile* and *Lavandula officinalis*: in vitro anthelmintic activities against *Haemonchus contortus*. *Parasite. Vector.* 11(1), 269.

Ferreira, L. E., Benincasa, B. I., Fachin, A. L., Franca, S. C., Contini, S. S., Chagas, A. C., et al. (2016). *Thymus vulgaris* L. essential oil and its main component thymol: Anthelmintic effects against *Haemonchus contortus* from sheep. *Vet Parasitol* 228, 70-76.

Fouche, G., Sakong, B. M., Adenubi, O. T., Pauw, E., Leboho, T., Wellington, K. W., et al. (2016). Anthelmintic activity of acetone extracts from South African plants used on egg hatching of *Haemonchus contortus*. *Onderstepoort J Vet Res* 83(1), 1-7.

Gaínza, Y. A., Domingues, L. F., Perez, O. P., Rabelo, M. D., López, E. R., de Souza Chagas, A. C. (2015). Anthelmintic activity in vitro of *Citrus sinensis* and *Melaleuca quinquenervia* essential oil from Cuba on *Haemonchus contortus*. *Ind Crops Prod* 76, 647-652.

Getachew, S., Ibrahim, N., Abebe, B., Eguale, T. (2012). In vitro evaluation of Anthelmintic activities of crude extracts of selected medicinal plants against *Haemonchus contortus* in AlemgenaWereda, Ethiopia. *APG* 3, 20-27.

Githiori, J., Höglund, J., Waller, P., Baker, R. (2004). Evaluation of anthelmintic properties of some plants used as livestock dewormers against *Haemonchus contortus* infections in sheep. *Parasitology* 129(2), 245-253.

Hajaji, S., Alimi, D., Jabri, M., Abuseir, S., Gharbi, M., Akkari, H. (2018). Anthelmintic activity of Tunisian chamomile (*Matricaria recutita* L.) against *Haemonchus contortus*. *J Helminthol* 92(2), 168-177.

Hamad, K. K., Iqbal, Z., Abbas, R. Z., Khan, A., Muhammad, G., Epperson, B. (2014). Combination of *Nicotiana tabacum* and *Azadirachta indica*: A Novel Substitute to Control Levamisole and Ivermectin-Resistant *Haemonchus contortus* in Ovine. *Pak Vet J* 34(1).

Hassan, M., Ra, M., Joshi, T., Yatoo, F. A., Habib, H. (2019). In vitro anthelmintic activity of *Abutilon theophrasti* Medik.(malvaceae) against eggs and l3 larvae of *Haemonchus contortus*. *In Vitro Mol Toxicol* Asian Journal of Pharmaceutical and Clinical Research(3).

Heckendorn, F., Häring, D. A., Maurer, V., Zinsstag, J., Langhans, W., Hertzberg, H. (2006). Effect of sainfoin (*Onobrychis viciifolia*) silage and hay on established populations of *Haemonchus contortus* and *Cooperia curticei* in lambs. *Vet Parasitol* 142(3-4), 293-300.

Hernández-Villegas, M., Borges-Argáez, R., Rodriguez-Vivas, R., Torres-Acosta, J., Méndez-González, M., Caceres-Farfan, M. (2011). Ovicidal and larvicidal activity of the crude extracts from Phytolacca icosandra against *Haemonchus contortus*. *Vet Parasitol* 179(1-3), 100-106.

Hördegen, P., Cabaret, J., Hertzberg, H., Langhans, W., Maurer, V. (2006). In vitro screening of six anthelmintic plant products against larval *Haemonchus contortus* with a modified methyl-thiazolyl-tetrazolium reduction assay. *J Ethnopharmacol* 108(1), 85-89.

Hussien, J., Urgessa, K., Regassa, F., Jemal, A., Abajebel, S., Hussien, N. (2011). Anthelmentic effects of the essential oil extracts of selected medicinal plants against *Haemonchus contortus*. *Int J Agric Res* 6(3), 290-298.

Idris, U. E., Adam, S. E., Tartour, G. (1982). The anthelmintic efficacy of *Artemisia herba-alba* against *Haemonchus contortus* infection in goats. *Natl Inst Anim Health Q (Tokyo)* 22(3), 138-143.

Iqbal, Z., Lateef, M., Ashraf, M., Jabbar, A. (2004). Anthelmintic activity of *Artemisia brevifolia* in sheep. *J Ethnopharmacol* 93(2-3), 265-268.

Iqbal, Z., Lateef, M., Jabbar, A., Akhtar, M. S., Khan, M. N. (2006a). Anthelmintic Activity of *Vernonia anthelmintica*. Seeds Against *Trichostrongylid* Nematodes of Sheep. *Pharm Biol* 44(8), 563-567.

Iqbal, Z., Lateef, M., Jabbar, A., Ghayur, M. N., Gilani, A. H. (2006b). In vitro and in vivo anthelmintic activity of *Nicotiana tabacum* L. leaves against gastrointestinal nematodes of sheep. *Phytother Res* 20(1), 46-48.

Iqbal, Z., Lateef, M., Jabbar, A., Muhammad, G., Khan, M. N. (2005). Anthelmintic activity of *Calotropis procera* (Ait.) Ait. F. flowers in sheep. *J Ethnopharmacol* 102(2), 256-261.

Iqbal, Z., Lateef, M., Khan, M. N., Jabbar, A., Akhtar, M. S. (2006c). Anthelmintic activity of *Swertia chirata* against gastrointestinal nematodes of sheep. *Fitoterapia* 77(6), 463-465.

Iqbal, Z., Munir, M. A., Khan, M., Akhtar, M. S., Javed, I., Of, O. (2001a). In vitro inhibitory effects of *Sorghum bicolor* on hatching and moulting of *Haemonchus contortus* eggs. *Prospects* 3, 451-453.

Iqbal, Z., Nadeem, Q. K., Khan, M., Akhtar, M., Waraich, F. N. (2001b). In vitro anthelmintic activity of *Allium sativum, Zingiber officinale, Curcurbita mexicana* and *Ficus religiosa*. *Int J Agric Biol* 3(4), 454-457.

Irum, S., Ahmed, H., Mukhtar, M., Mushtaq, M., Mirza, B., Donskow-Łysoniewska, K., et al. (2015). Anthelmintic activity of *Artemisia vestita* Wall ex DC. and *Artemisia maritima* L. against *Haemonchus contortus* from sheep. *Vet Parasitol* 212(3-4), 451-455.

Jabbar, A., Zaman, M. A., Iqbal, Z., Yaseen, M., Shamim, A. (2007). Anthelmintic activity of *Chenopodium album* (L.) and *Caesalpinia crista* (L.) against trichostrongylid nematodes of sheep. *J Ethnopharmacol* 114(1), 86-91.

Jaheed, E., Mohamed, A. H., Hassan, N. M. F., Mahran, K. M. A., Nasr, S. M., Abou-Zeina, H. A. A. (2019). Evaluation of the curative effect of *Balanites aegyptiaca* fruits ethanolic extract on Haemonchosis experimentally induced in Egyptian Baladi goats: phytoanalytical, parasitological and hematological studies. *J Parasit Dis* 43(4), 638-650. doi: 10.1007/s12639-019-01143-1

Kamaraj, C., Rahuman, A. A. (2011). Efficacy of anthelmintic properties of medicinal plant extracts against *Haemonchus contortus*. *Res Vet Sci* 91(3), 400-404.

Kamaraj, C., Rahuman, A. A., Bagavan, A., Mohamed, M. J., Elango, G., Rajakumar, G., et al. (2010). Ovicidal and larvicidal activity of crude extracts of *Melia azedarach* against *Haemonchus contortus* (Strongylida). *Parasitol Res* 106(5), 1071-1077.

Kamaraj, C., Rahuman, A. A., Elango, G., Bagavan, A., Zahir, A. A. (2011). Anthelmintic activity of botanical extracts against sheep gastrointestinal nematodes, *Haemonchus contortus*. *Parasitol Res* 109(1), 37-45.

Karim, M. A., Islam, M. R., Lovelu, M. A., Nahar, S. F., Dutta, P. K., Talukder, M. H. (2019). In vitro evaluation of anthelmintic activity of tannin-containing plant Artemisia extracts against *Haemonchus contortus* from goat. *JBAU* 17(3), 363-368.

Katiki, L., Chagas, A., Takahira, R. K., Juliani, H., Ferreira, J., Amarante, A. F. T. d. (2012). Evaluation of *Cymbopogon schoenanthus* essential oil in lambs experimentally infected with *Haemonchus contortus*. *Vet Parasitol* 186(3-4), 312-318.

Katiki, L., Gomes, A., Barbieri, A., Pacheco, P., Rodrigues, L., Veríssimo, C., et al. (2017). *Terminalia catappa*: chemical composition, in vitro and in vivo effects on *Haemonchus contortus*. *Vet Parasitol* 246, 118-123.

Krstin, S., Sobeh, M., Braun, M. S., Wink, M. (2018). Anti-Parasitic Activities of *Allium sativum* and *Allium cepa* against *Trypanosoma b. brucei* and *Leishmania tarentolae*. *Medicines* 5(2), 37.

Lachenmeier, D. W. (2010). Wormwood (*Artemisia absinthium* L.)—A curious plant with both neurotoxic and neuroprotective properties? *J Ethnopharmacol* 131(1), 224-227.

Lara TF, M., ML Bevilaqua, C., MB de Oliveira, L., LF Camurca-Vasconcelos, A., da S Viera, L., R Oliveira, F., et al. (2009). In vitro ovicidal and larvicidal activity of *Eucalyptus globulus* essential oil on *Haemonchus contortus*. *Rev Bras Parasitol Vet* 18, 62-66.

Lone, B. A., Bandh, S. A., Chishti, M. Z., Bhat, F. A., Tak, H., Nisa, H. (2013). Anthelmintic and antimicrobial activity of methanolic and aqueous extracts of *Euphorbia helioscopia* L. *Trop Anim Health Prod* 45(3), 743-749.

Lone, B. A., Chishti, M., Bhat, F. A., Tak, H., Bandh, S. A. (2012). In vitro and in vivo anthelmintic activity of *Euphorbia helioscopia* L. *Vet Parasitol* 189(2-4), 317-321.

Lopes, L. G., Silva, M. H., Figueiredo, A., Canuto, K. M., Brito, E. S., Ribeiro, P. R. V., et al. (2018). The intake of dry cashew apple fiber reduced fecal egg counts in *Haemonchus contortus*-infected sheep. *Exp Parasitol* 195, 38-43. doi: 10.1016/j.exppara.2018.10.004

Macedo, I. T., Bevilaqua, C. M., de Oliveira, L. M., Camurça-Vasconcelos, A. L., Morais, S. M., Machado, L. K., et al. (2012). In vitro activity of *Lantana camara, Alpinia zerumbet, Mentha villosa* and *Tagetes minuta* decoctions on *Haemonchus contortus* eggs and larvae. *Vet Parasitol* 190(3-4), 504-509.

Macedo, I. T., Bevilaqua, C. M., de Oliveira, L. M., Camurça-Vasconcelos, A. L., Vieira, L. d. S., Oliveira, F. R., et al. (2010). Anthelmintic effect of *Eucalyptus staigeriana* essential oil against goat gastrointestinal nematodes. *Vet Parasitol* 173(1-2), 93-98.

Macedo, I. T. F., Oliveira, L. M. B., André, W. P. P., Araújo Filho, J. V., Santos, J., Rondon, F. C. M., et al. (2019). Anthelmintic effect of *Cymbopogon citratus* essential oil and its nanoemulsion on sheep gastrointestinal nematodes. *Rev Bras Parasitol Vet* 28(3), 522-527. doi: 10.1590/s1984-29612019065

Maciel, M., Morais, S. M., Bevilaqua, C., Camurça-Vasconcelos, A., Costa, C., Castro, C. (2006). Ovicidal and larvicidal activity of *Melia azedarach* extracts on *Haemonchus contortus*. *Vet Parasitol* 140(1-2), 98-104.

Mahmoud, O., Adam, S., Tartour, G. (1979). The effects of *Calotropis procera* on small ruminants: II. Effects of administration of the latex to sheep and goats. *J Comp Pathol* 89(2), 251-263.

Malik, S., de Mesquita, L. S. S., Silva, C. R., de Mesquita, J. W. C., de Sá Rocha, E., Bose, J., et al. (2019). Chemical Profile and Biological Activities of Essential Oil from *Artemisia vulgaris* L. Cultivated in Brazil. *Pharmaceuticals (Basel)* 12(2). doi: 10.3390/ph12020049

Maphosa, V., Masika, P. J. (2012). Anthelmintic screening of fractions of *Elephantorrhiza elephantina* root extract against *Haemonchus contortus*. *Trop Anim Health Prod* 44(1), 159-163.

Maphosa, V., Masika, P. J., Bizimenyera, E. S., Eloff, J. (2010). In-vitro anthelminthic activity of crude aqueous extracts of *Aloe ferox, Leonotis leonurus* and *Elephantorrhiza elephantina* against *Haemonchus contortus*. *Trop Anim Health Prod* 42(2), 301-307.

Marie-Magdeleine, C., Mahieu, M., D’alexis, S., Philibert, L., Archimede, H. (2010). In vitro effects of *Tabernaemontana citrifolia* extracts on *Haemonchus contortus*. *Res Vet Sci* 89(1), 88-92.

Marie-Magdeleine, C., Udino, L., Philibert, L., Bocage, B., Archimede, H. (2014). In vitro effects of *Musa x paradisiaca* extracts on four developmental stages of *Haemonchus contortus*. *Res Vet Sci* 96(1), 127-132.

Martínez-Ortiz-de-Montellano, C., de Jesús Torres-Acosta, J. F., Fourquaux, I., Sandoval-Castro, C. A., Hoste, H. (2019). Ultrastructural study of adult *Haemonchus contortus* exposed to polyphenol-rich materials under in vivo conditions in goats. *Parasite* 26.

Maurer, H. (2001). Bromelain: biochemistry, pharmacology and medical use. *Cell Mol Life Sci* 58(9), 1234-1245.

Minho, A., Bueno, I., Louvandini, H., Jackson, F., Gennari, S., Abdalla, A. (2008). Effect of *Acacia molissima* tannin extract on the control of gastrointestinal parasites in sheep. *Anim Feed Sci Technol* 147(1-3), 172-181.

Mondal, H., Hossain, H., Awang, K., Saha, S., Mamun-Ur-Rashid, S., Islam, M. K., et al. (2015). Anthelmintic activity of ellagic acid, a major constituent of *Alternanthera sessilis* against *Haemonchus contortus*. *Pak Vet J* 35(1).

Monglo, D., Njongmeta, L., Musongong, G., Ngassoum, M., Nukenine, E. (2006). Evaluation of anthelminthic potential of ethanolic plant extracts from Northern Cameroon against eggs and infective larvae of *Haemonchus contortus*. *J Biol Sci* 6(2), 426-433.

Monteiro, M. V. B., Bevilaqua, C. M., Morais, S. M., Machado, L. K. A., Camurca-Vasconcelos, A. L. F., Campello, C. C., et al. (2011). Anthelmintic activity of *Jatropha curcas* L. seeds on *Haemonchus contortus*. *Vet Parasitol* 182(2-4), 259-263.

Morais-Costa, F., Bastos, G., Soares, A., Costa, E., Vasconcelos, V., Oliveira, N., et al. (2016). In vitro and in vivo action of *Piptadenia viridiflora* (Kunth) Benth against *Haemonchus contortus* in sheep. *Vet Parasitol* 223, 43-49.

Nery, P., Nogueira, F., Martins, E., Duarte, E. (2010). Effects of *Anacardium humile* leaf extracts on the development of gastrointestinal nematode larvae of sheep. *Vet Parasitol* 171(3-4), 361-364.

Njoku, C. J., Asuzu, I. U. (1998). The anthelmintic effects of the leaf extract of *Ocimum gratissimum* (L.). *Phytomedicine* 5(6), 485-488. doi: 10.1016/s0944-7113(98)80047-0

Nsereko, G., Emudong, P., Omujal, J., Acai, J., Kungu, J. M., Kabi, F., et al. (2019). Comparison of the efficacy of crude methanolic extracts of *Cassia occidentalis* and *Euphorbia hirta* with levamisole-HCL against gastrointestinal nematodes of economic importance to goat production in Uganda. *Trop Anim Health Prod* 51(8), 2269-2278. doi: 10.1007/s11250-019-01939-6

Oliveira, G. L., Vieira, T. M., Nunes, V. F., de Oliveria Ruas, M., Duarte, E. R., de Lima Moreira, D., et al. (2014). Chemical composition and efficacy in the egg-hatching inhibition of essential oil of *Piper aduncum* against *Haemonchus contortus* from sheep. *Rev Bras Farmacogn* 24(3), 288-292.

Oliveira, L., Bevilaqua, C., Costa, C., Macedo, I., Barros, R., Rodrigues, A., et al. (2009). Anthelmintic activity of *Cocos nucifera* L. against sheep gastrointestinal nematodes. *Vet Parasitol* 159(1), 55-59.

Palacios-Landín, J., Mendoza-de Gives, P., Salinas-Sánchez, D. O., López-Arellano, M. E., Liébano-Hernández, E., Hernández-Velázquez, V. M., et al. (2015). In vitro and in vivo nematocidal activity of *Allium sativum* and *Tagetes erecta* extracts against *Haemonchus contortus*. *Turk Parazitol Derg* 39(4), 260.

Patel, D., Desai, S., Devkar, R., Ramachandran, A. (2012). Acute and sub-chronic toxicological evaluation of hydro-methanolic extract of *Coriandrum Sativum* L. seeds. *EXCLI J* 11, 566.

Pessoa, L., Morais, S., Bevilaqua, C., Luciano, J. (2002). Anthelmintic activity of essential oil of *Ocimum gratissimum* Linn. and eugenol against *Haemonchus contortus*. *Vet Parasitol* 109(1-2), 59-63.

Piza, M., Féboli, A., Augusto, J., Anjos, L., Laurentiz, A., Royo, V., et al. (2019). In vitro ovicidal and larvicidal activity of *Psidium cattleianum* Sabine leaves against gastrointestinal nematodes of naturally infected sheep. *Bol Ind Anim* 76, 1-8.

Qi, H., Wang, W., Dai, J., Zhu, L. (2015). In vitro anthelmintic activity of *Zanthoxylum simulans* essential oil against *Haemonchus contortus*. *Vet Parasitol* 211(3-4), 223-227.

Ribeiro, J., Ribeiro, W., Camurça-Vasconcelos, A., Macedo, I., Santos, J., Paula, H., et al. (2014). Efficacy of free and nanoencapsulated *Eucalyptus citriodora* essential oils on sheep gastrointestinal nematodes and toxicity for mice. *Vet Parasitol* 204(3-4), 243-248.

Ribeiro, W. L., Andre, W. P., Cavalcante, G. S., de Araújo-Filho, J. V., Santos, J. M., Macedo, I. T., et al. (2017). Effects of *Spigelia anthelmia* decoction on sheep gastrointestinal nematodes. *Small Rumin Res* 153, 146-152.

Saleem, U., Ahmad, B., Ahmad, M., Erum, A., Hussain, K., Irfan Bukhari, N. (2016). Is folklore use of *Euphorbia helioscopia* devoid of toxic effects? *Drug Chem Toxicol* 39(2), 233-237.

Santoro, G., Cardoso, M., Guimarães, L., Freire, J., Soares, M. (2007). Anti-proliferative effect of the essential oil of *Cymbopogon citratus* (DC) Stapf (lemongrass) on intracellular amastigotes, bloodstream trypomastigotes and culture epimastigotes of *Trypanosoma cruzi* (Protozoa: Kinetoplastida). *Parasitology* 134(11), 1649-1656.

Sirama, V., Kokwaro, J., Owuor, B., Yusuf, A., Kodhiambo, M. (2015). In-vitro anthelmintic activity of *Vernonia amygdalina* Del.(asteraceae) roots using adult *Haemonchus contortus* worms. *Int J Pharmacol Res* 5(1), 1-2.

Soares, A. M., Oliveira, J. T., Rocha, C. Q., Ferreira, A. T., Perales, J., Zanatta, A. C., et al. (2018). *Myracrodruon urundeuva* seed exudates proteome and anthelmintic activity against *Haemonchus contortus*. *PLoS One* 13(7), e0200848.

Squires, J. M., Ferreira, J. F., Lindsay, D. S., Zajac, A. M. (2011). Effects of artemisinin and Artemisia extracts on *Haemonchus contortus* in gerbils (*Meriones unguiculatus*). *Vet Parasitol* 175(1-2), 103-108.

Tadesse, D., Eguale, T., Giday, M., Mussa, A. (2009). Ovicidal and larvicidal activity of crude extracts of *Maesa lanceolata* and *Plectranthus punctatus* against *Haemonchus contortus*. *J Ethnopharmacol* 122(2), 240-244.

Tariq, K., Chishti, M., Ahmad, F., Shawl, A. (2008). Anthelmintic efficacy of *Achillea millifolium* against gastrointestinal nematodes of sheep: in vitro and in vivo studies. *J Helminthol* 82(3), 227-233.

Tariq, K., Chishti, M., Ahmad, F., Shawl, A. (2009). Anthelmintic activity of extracts of *Artemisia absinthium* against ovine nematodes. *Vet Parasitol* 160(1-2), 83-88.

Tayler, N. M., De Jesús, R., Spadafora, R., Coronado, L. M., Spadafora, C. (2020). Antiplasmodial activity of *Cocos nucifera* leaves in *Plasmodium berghei*-infected mice. *J Parasit Dis*, 1-9.

Váradyová, Z., Pisarčíková, J., Babják, M., Hodges, A., Mravčáková, D., Kišidayová, S., et al. (2018). Ovicidal and larvicidal activity of extracts from medicinal-plants against *Haemonchus contortus*. *Exp Parasitol* 195, 71-77.

Vargas-Magaña, J., Torres-Acosta, J., Aguilar-Caballero, A., Sandoval-Castro, C., Hoste, H., Chan-Pérez, J. (2014). Anthelmintic activity of acetone–water extracts against *Haemonchus contortus* eggs: interactions between tannins and other plant secondary compounds. *Vet Parasitol* 206(3-4), 322-327.

Veerakumari, L., Chitra, N. (2016). Effect of *Allium sativum* on the Motility and Acetylcholinesterase of *Haemonchus contortus*. *IJSR* 5(1), 883-887.

Zamilpa, A., García-Alanís, C., López-Arellano, M. E., Hernández-Velázquez, V. M., Valladares-Cisneros, M. G., Salinas-Sánchez, D. O., et al. (2019). In vitro nematicidal effect of *Chenopodium ambrosioides* and *Castela tortuosa* n-hexane extracts against *Haemonchus contortus* (Nematoda) and their anthelmintic effect in gerbils. *J Helminthol* 93(4), 434-439. doi: 10.1017/s0022149x18000433

Zhu, L., Dai, J., Yang, L., Qiu, J. (2013a). Anthelmintic activity of *Arisaema franchetianum* and *Arisaema lobatum* essential oils against *Haemonchus contortus*. *J Ethnopharmacol* 148(1), 311-316.

Zhu, L., Dai, J., Yang, L., Qiu, J. (2013b). In vitro ovicidal and larvicidal activity of the essential oil of *Artemisia lancea* against *Haemonchus contortus* (Strongylida). *Vet Parasitol* 195(1-2), 112-117.
